# A TREM2-activating antibody with a blood–brain barrier transport vehicle enhances microglial metabolism in Alzheimer’s disease models

**DOI:** 10.1038/s41593-022-01240-0

**Published:** 2023-01-12

**Authors:** Bettina van Lengerich, Lihong Zhan, Dan Xia, Darren Chan, David Joy, Joshua I. Park, David Tatarakis, Meredith Calvert, Selina Hummel, Steve Lianoglou, Michelle E. Pizzo, Rachel Prorok, Elliot Thomsen, Laura M. Bartos, Philipp Beumers, Anja Capell, Sonnet S. Davis, Lis de Weerd, Jason C. Dugas, Joseph Duque, Timothy Earr, Kapil Gadkar, Tina Giese, Audrey Gill, Johannes Gnörich, Connie Ha, Malavika Kannuswamy, Do Jin Kim, Sebastian T. Kunte, Lea H. Kunze, Diana Lac, Kendra Lechtenberg, Amy Wing-Sze Leung, Chun-Chi Liang, Isabel Lopez, Paul McQuade, Anuja Modi, Vanessa O. Torres, Hoang N. Nguyen, Ida Pesämaa, Nicholas Propson, Marvin Reich, Yaneth Robles-Colmenares, Kai Schlepckow, Luna Slemann, Hilda Solanoy, Jung H. Suh, Robert G. Thorne, Chandler Vieira, Karin Wind-Mark, Ken Xiong, Y. Joy Yu Zuchero, Dolo Diaz, Mark S. Dennis, Fen Huang, Kimberly Scearce-Levie, Ryan J. Watts, Christian Haass, Joseph W. Lewcock, Gilbert Di Paolo, Matthias Brendel, Pascal E. Sanchez, Kathryn M. Monroe

**Affiliations:** 1 Denali Therapeutics, Inc., South San Francisco, CA USA; 2 German Center for Neurodegenerative Diseases (DZNE), Munich, Germany; 3 Department of Nuclear Medicine, University Hospital of Munich, Ludwig Maximilians University, Munich, Germany; 4 Biomedical Center (BMC), Division of Metabolic Biochemistry, Faculty of Medicine, Ludwig Maximilians University, Munich, Germany; 5 Takeda Pharmaceutical Company, Cambridge, MA USA; 6 Graduate School of Systemic Neurosciences, Ludwig Maximilians University Munich, Planegg-Martinsried, Germany; 7 Munich Cluster for Systems Neurology (SyNergy), Munich, Germany

**Keywords:** Microglia, Blood-brain barrier, Alzheimer's disease, Neuroimmunology, Drug delivery

## Abstract

Loss-of-function variants of TREM2 are associated with increased risk of Alzheimer’s disease (AD), suggesting that activation of this innate immune receptor may be a useful therapeutic strategy. Here we describe a high-affinity human TREM2-activating antibody engineered with a monovalent transferrin receptor (TfR) binding site, termed antibody transport vehicle (ATV), to facilitate blood–brain barrier transcytosis. Upon peripheral delivery in mice, ATV:TREM2 showed improved brain biodistribution and enhanced signaling compared to a standard anti-TREM2 antibody. In human induced pluripotent stem cell (iPSC)-derived microglia, ATV:TREM2 induced proliferation and improved mitochondrial metabolism. Single-cell RNA sequencing and morphometry revealed that ATV:TREM2 shifted microglia to metabolically responsive states, which were distinct from those induced by amyloid pathology. In an AD mouse model, ATV:TREM2 boosted brain microglial activity and glucose metabolism. Thus, ATV:TREM2 represents a promising approach to improve microglial function and treat brain hypometabolism found in patients with AD.

## Abstract (web summary)

van Lengerich et al. developed a human TREM2 antibody with a transport vehicle (ATV) that improves brain exposure and biodistribution in mouse models. ATV:TREM2 promotes microglial energetic capacity and metabolism via mitochondrial pathways.

## Main

Alzheimer’s disease (AD) is the leading cause of dementia and represents a major global unmet medical need. Human genome-wide association studies identified numerous variants implicating microglia in modulating risk for late-onset AD (LOAD)^1,2^. Microglia are the brain’s resident innate immune cells that can resolve disturbances and help maintain brain homeostasis.

A key gene modulating LOAD risk is *TREM2* (triggering receptor expressed on myeloid cells 2), encoding a lipid receptor expressed in microglia in the central nervous system (CNS)^3,4^. TREM2 signals via DAP12, an immunoreceptor tyrosine-based activating motif (ITAM) domain-containing receptor, which phosphorylates Syk for pathway activation. TREM2 is regulated by intracellular trafficking and proteolytic cleavage of the ectodomain by ADAM10/17 (refs. 5–7). TREM2 controls key microglial functions, such as phagocytosis^7,8^, migration^9^, lipid processing^10^, proliferation, lysosomal degradation and metabolism^11^. TREM2 was shown to be required for amyloid plaque compaction in AD mouse models, myelin debris clearance in demyelinating models and neuronal health^10–14^.

TREM2 coding variants significantly increase AD risk^15,16^. R47H reduces lipid ligand affinity^3,10,17,18^, and H157Y abrogates signaling by increasing cell surface shedding of TREM2 (refs. 19–21). P522R is a hypermorphic variant in *PLCG2* (phospholipase C gamma 2), a downstream signaling mediator, that is protective in AD^22–25^. Finally, cerebrospinal fluid (CSF) biomarker studies indicate that higher soluble TREM2 is associated with slower AD progression^26,27^. Overall, these studies suggest that TREM2 loss of function (LOF) contributes to AD risk, and increased TREM2 function may be beneficial in AD.

Previous studies of pharmacological TREM2 activation largely report standard IgG antibodies that enter the brain via non-specific uptake due to the blood–brain barrier (BBB)^2,28–34^. Here we describe mechanisms of a novel therapeutic candidate, ATV:TREM2, a high-affinity human TREM2 antibody engineered with a monovalent transferrin receptor (TfR) binding site in the Fc domain to enable active transport into the CNS^35^. ATV:TREM2 demonstrated improved brain exposure and CNS biodistribution compared to anti-TREM2. ATV:TREM2 promoted mitochondrial metabolic pathways, such as lipid catabolism and glucose oxidation in microglia. In AD mouse models, ATV:TREM2 induced transcription of metabolic pathway genes and increased brain glucose uptake by fluorodeoxyglucose-positron emission tomography (FDG-PET). These studies provide new insights into the mechanisms by which ATV:TREM2, a therapeutic candidate, increases microglial functions.

## Results

### ATV enhances brain exposure and activity of a TREM2 antibody

**Fig. 1:**
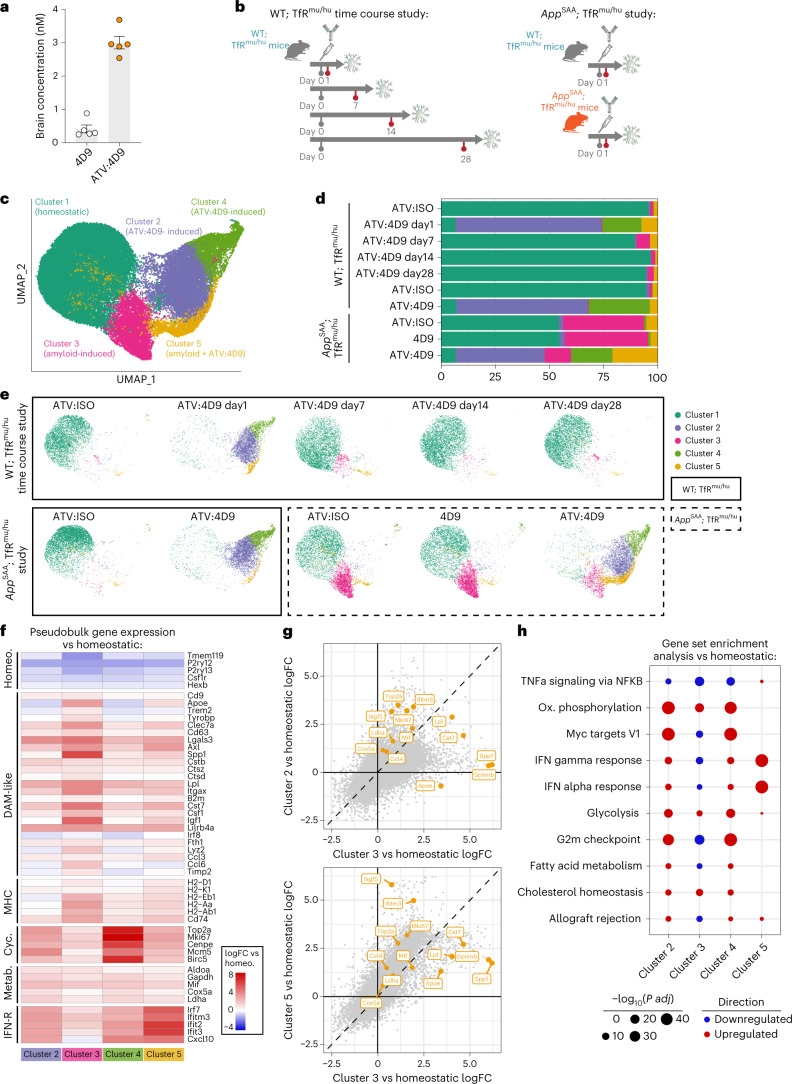
ATV:4D9 induces temporally dynamic microglial states distinct from amyloid pathology by single-cell analysis. **a**, Antibodies were detected by human IgG ELISA in whole brain lysates 1 day after IV dose of 10 mg kg^−1^ ATV:4D9 or 4D9 (*n* = 5 mice). **b**, Designs for WT;TfR^mu/hu^ and *App*^SAA^;TfR^mu/hu^ studies. Mice were injected with a single IV 10 mg kg^−1^ dose of ATV:4D9 or ATV:ISO and sacrificed at indicated timepoints (*n* = 3 WT; TfR^mu/hu^ mice and *n* = 4 *App*^SAA^;TfR^mu/hu^ mice). **c**, Integrated UMAP projection of 49,000 total cells from all mice in both studies. The WT;TfR^mu/hu^ dataset consisted of 102,043 cells, and the *App*^SAA^;TfR^mu/hu^ dataset consisted of 74,758 cells (Extended Data Fig. 1a,b). Five distinct clusters of microglia were identified. **d**, Stacked bar plots of clusters distributed per group for both studies. Clusters are shown as percentages of the whole microglial compartment averaged for each biological replicate. **e**, UMAP projection split by group for cluster distribution. Data from WT;TfR^mu/hu^ mice are boxed with solid lines, and data from *App*^SAA^;TfR^mu/hu^ mice are boxed with dashed lines. **f**, Heat map of average log_2_FC in each cluster compared to the ‘homeostatic’ cluster. Pseudobulk expression was generated by summing every counts per gene. Each mouse was treated as a biological replicate, and differential expression (DE) was performed for each cluster versus ‘homeostatic’ using limma. **g**, Scatter plots of the comparison of versus ‘homeostatic’ log_2_FC for every gene between clusters 3 and 2 (top) and clusters 3 and 5 (bottom). In the top-right quadrant, genes falling below the dashed line are more upregulated in cluster 3, whereas genes above the dashed line are further upregulated in cluster 2 (top) or cluster 5 (bottom). Canonical DAM genes and other genes of interest are highlighted in orange. **h**, Dot plot showing GSEA for each cluster using DE versus ‘homeostatic.’ Signatures were taken from the hallmark gene signatures collection. Dot size is inversely proportional to log_10_(corrected *P* value), and color indicates direction. No dot indicates a non-significant result. FC, fold change. Source data

A mouse TREM2-specific antibody, 4D9, was previously shown to enhance protective microglial functions and reduce amyloid pathology in an AD mouse model^31^. To evaluate ATV technology, we generated ATV:4D9 on a human IgG backbone with LALA (Leu234Ala) mutations to minimize FcR binding. For in vivo studies, we employed a mouse model with the apical domain of human TfR knocked-in (TfR^mu/hu^) to the mouse TfR locus, as ATV is not cross-reactive^35^. ATV:4D9 was detected at higher brain concentrations than 4D9 1 day after a 10 mg kg^−1^ intravenous (IV) dose (Fig. 1a). Capillary depletion^35–37^ was used to assess BBB transcytosis. ATV:4D9 was increased in vascular and parenchymal fraction lysates (Supplementary Fig. 1a–c), which were successfully separated based on absence of endothelial markers CD31 and CLDN5 in the parenchymal fraction (Supplementary Fig. 1d). ATV:4D9 showed higher microglial co-localization compared to 4D9 by immunohistochemistry (IHC) (Supplementary Fig. 1e–h). Thus, ATV:TREM2 shows improved parenchymal delivery and targeting to microglia.

To determine if TfR binding impacts TREM2 antibody activity independently of its ability to increase brain exposure, we tested microglial responses with similar brain concentrations of ATV:4D9 and 4D9 (Supplementary Fig. 1i) and found that only ATV:4D9 significantly increased IBA1 (Supplementary Fig. 1j,k). Therefore, ATV enhances microglia responses of a TREM2 antibody despite matching brain exposures for anti-TREM2.

### ATV:4D9 induces microglial states distinct from amyloid

**Extended Data Fig. 1:**
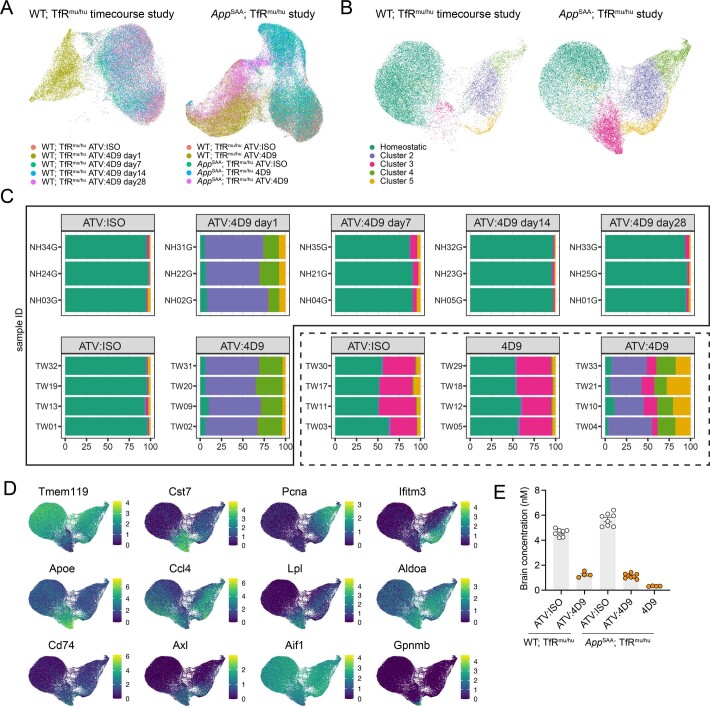
ATV:4D9 induces temporally dynamic microglial states distinct from amyloid pathology. **(a)** UMAP projections of individually processed data sets for WT; TfR^mu/hu^ timecourse and TfR^mu/hu^; *App*^SAA^ studies. Microglia are color coded according to their experimental group. **(b)** Combined UMAP of integrated data by study. Microglia are color coded by unbiased cluster assignment. **(c)** Stacked barplots showing the proportion of microglia per biological replicate by cluster. Plots are grouped by experimental group, and each bar represents a biological replicate within that group. Barplot color scheme is consistent with clusters in **b**. **(d)** Feature plots showing expression of selected individual genes. Microglia are color coded according to log normalized expression of each gene. **(e)** Antibody concentrations detected in whole brain lysate from either WT; TfR^mu/hu^ or TfR^mu/hu^; *App*^SAA^ mice dosed with 10 mg kg^−1^ ATV:ISO or ATV:4D9 (n = 8 mice/group, except for ATV:4D9 WT;TfR^mu/hu^ and 4D9 APP^SAA^; TfR^mu/hu^ n = 4 mice/group).

Other groups reported that standard IgG TREM2-activating antibodies shift microglia states in vivo^32,33^. We hypothesized that ATV:TREM2 may have a broader impact on microglia via increased brain exposure. We evaluated the transcriptional dynamics of ATV:4D9 and the duration of the microglial response using single-cell RNA sequencing (scRNA-seq) over a time course after a single dose (Extended Data Fig. 1a,b) and in the context of amyloid pathology^38^ (Fig. 1b and Extended Data Fig. 1a,b). Microglial state changes were highly consistent within groups (Extended Data Fig. 1c), and brain exposure was not affected by mouse genotype (Extended Data Fig. 1e).

Most microglia responded to ATV:4D9; five distinct clusters were identified after integration, uniform manifold approximation and projection (UMAP) and Louvain clustering (Fig. 1c). Most microglia were in the homeostatic cluster in isotype control-treated wild-type (WT) mice, whereas ATV:4D9 induced substantial shifts to clusters 2 and 4 after 1 day. Responsive microglial clusters largely returned to homeostasis by day 7; however, a small disease-associated microglia (DAM) cluster persisted (Fig. 1d).

ATV:4D9 increased microglial state diversity in *App*^SAA^;TfR^mu/hu^ mice (Fig. 1d,e). In all four microglial clusters, responsive genes were increased compared to cluster 1. The amyloid pathology-induced cluster 3 phenocopied DAM^11,39,40^ (Fig. 1f and Supplementary Table 5). The ATV:4D9-induced cluster 4 upregulated cell cycle genes and reduced expression of DAM genes compared to cluster 3 (Fig. 1f,g). Cluster 5 was induced by ATV:4D9 and amyloid pathology with upregulated IFN-stimulated genes (ISGs) with a modest upregulation of DAM genes (Fig. 1f,g).

**Extended Data Fig. 2:**
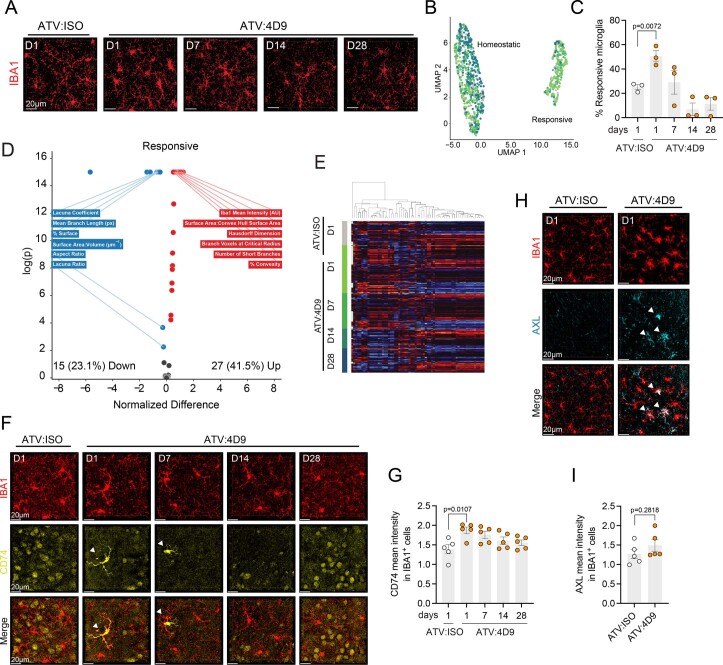
ATV:4D9 induces temporally dynamic microglial morphology and marker expression. **(a)** Representative morphometric images of microglia in the cortex 1 day post dose with ATV:ISO or ATV:4D9 (10 mg kg^−1^) at day 1-, 7-, 14-, and 28-days post dose. **(b)** UMAP plot of all microglia over time. **(c)** Percentage of microglia in the responsive cluster as a proportion of all segmented microglia over time (n = 3 male mice per group, two-tailed unpaired t-test between ATV:ISO and ATV:4D9 at day 1, mean ± SEM). **(d)** Volcano plot showing the top 6 differentially high and low normalized features comparing the responsive cluster to the homeostatic cluster. **(e)** Heatmap of normalized features for all segmented microglia (1,143 total cells) over time, measured across 65 morphometric and immunohistochemical features, grouped by treatment (rows) with features hierarchically clustered (columns). **(f)** Representative images of cortical brain sections co-stained with Iba1 and CD74 at 1-, 7-, 14-, 28-days days post 10 mg kg^−1^ dose of ATV:ISO or ATV:4D9. CD74^+^ microglia are noted with white arrows. (**g**) Mean intensity of CD74 staining within segmented IBA1^+^ microglia normalized to background CD74 intensity at each timepoint (n = 5 mice/group, two-tailed unpaired t-test between ATV:ISO and ATV:4D9 at day 1, mean ± SEM). **(h)** Representative images of cortical brain sections stained for IBA1 and AXL 1 day post dose of ATV:ISO or ATV:4D9. Double positive microglia are noted with white arrows. **(i)** Quantification of mean intensity of AXL staining within segmented IBA1^+^ microglia normalized to background (n = 5 mice/group, two-tailed unpaired t-test, mean ± SEM).

We then performed gene set enrichment analysis (GSEA) (Fig. 1h and Supplementary Table 5). Cluster 3 showed modest enrichment of glycolysis, oxidative phosphorylation and lipid metabolism signatures, suggesting that amyloid pathology imposes a metabolic demand on microglia. Metabolic signatures were further upregulated by ATV:4D9 in clusters 2 and 4. ATV:4D9 reduced amyloid-induced cluster 3 and expanded metabolic clusters 2 and 4 in *App*^SAA^;TfR^mu/hu^ mice (Fig. 1d,c). Therefore, ATV:4D9 promoted transcription of multiple metabolic pathways in microglia. We conducted IHC on brain sections and detected increased CD74 and AXL in a subset of IBA1^+^ microglia, consistent with the transcriptional profiling (Extended Data Figs. 1d and 2f–l).

We next observed profound morphological changes consistent with a responsive microglial state, including higher fractal dimension, convexity, solidity and a larger number of short branches 1 day and 7 days after ATV:4D9 dose. These responsive morphological features were reversible, as homeostatic features were observed 14 days and 28 days after dosing (Extended Data Fig. 2a,c). Thus, ATV:4D9 induced dynamic microglial states transcriptionally and morphologically.

### ATV:TREM2 is a human TREM2 stalk region binding antibody

**Fig. 2:**
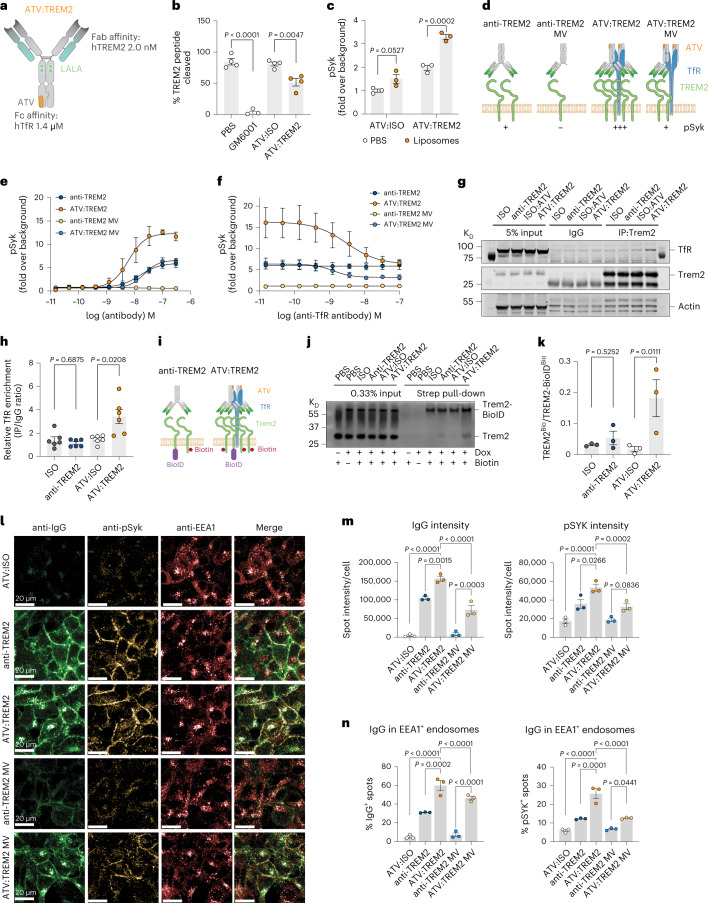
ATV:TREM2 is a novel activating antibody that potentiates receptor clustering and endocytosis to enhance TREM2 signaling. **a**, Antibody schematic of ATV:TREM2 with human TREM2 Fab affinity and ATV binding site in the Fc domain and effectorless Fc mutations. **b**, Fluorescence polarization (FP)-based detection of TREM2 stalk peptide cleavage by recombinant ADAM17 (*n* = 4 independent experiments; two-tailed unpaired *t*-test, mean ± s.e.m.). **c**, ATV:TREM2 and PS liposome co-treatment enhanced pSyk in WT iMG (*n* = 3 independent experiments; Tukey’s multiple comparisons test, mean ± s.e.m.). **d**, Schematic illustrating ATV and TREM2 Fab valency effects on pSyk signaling. Antibodies include anti-TREM2 and ATV:TREM2 with MV and BV Fabs. **e**, hTREM2-DAP12 HEK293 cells treated with a dose response of antibodies for 5 minutes, followed by pSyk detection (*n* = 3 independent experiments; mean ± s.e.m.). **f**, pSyk is blocked by co-treatment of ATV:TREM2 and anti-TfR. hTREM2-DAP12 HEK293 cells were dosed with 100 nM TREM2 antibodies and a titration anti-TfR. pSyk was detetced 5 minutes after treatment (*n* = 3 independent experiments; mean ± s.e.m.). **g**, TfR and TREM2 co-IP. hTREM2-DAP12 HEK293 cells were treated with 100 nM per antibody for 5 minutes, followed by IP on cell lysates with anti-TREM2. TfR was detected by western blot. **h**, Co-IP quantification of western blot data in **g** (*n* = 6 independent experiments; Wilcoxon test for ISO versus anti-TREM2, two-tailed paired *t*-test for ATV:ISO versus ATV:TREM2, mean ± s.e.m.). **i**, Schematic of BioID TREM2 receptor clustering assay strategy. **j**, Representative western blot detection of biotinylated TREM2 after streptavidin IP. TREM2-BioID expression was induced 24 hours before the assay with 2 ng ml^−1^ of Dox. Cells were treated with 100 nM antibody and 2 µM biotin. **k**, Quantification of western blot from **j** (*n* = 4 independent experiments; ratio of two-tailed paired *t*-test, mean ± s.e.m.). **l**, Representative immunofluorescence images of hTREM2-DAP12 HEK293 cells stained for IgG (green), pSyk (yellow) and EEA1 (red). Cells were treated with 10 nM per antibody for 10 min. **m**, Quantification of spot intensity for IgG and pSyk immunofluorescence per cell (*n* = 3 independent experiments with 3,000–5,000 cells per condition; Tukey’s multiple comparisons test, mean ± s.e.m.). **n**, Quantification of percent of IgG or pSyk spots localized within EEA1^+^ endosomes (*n* = 3 independent experiments with 3,000–5,000 cells per condition; Tukey’s multiple comparisons test, mean ± s.e.m.). Source data

**Extended Data Fig. 3:**
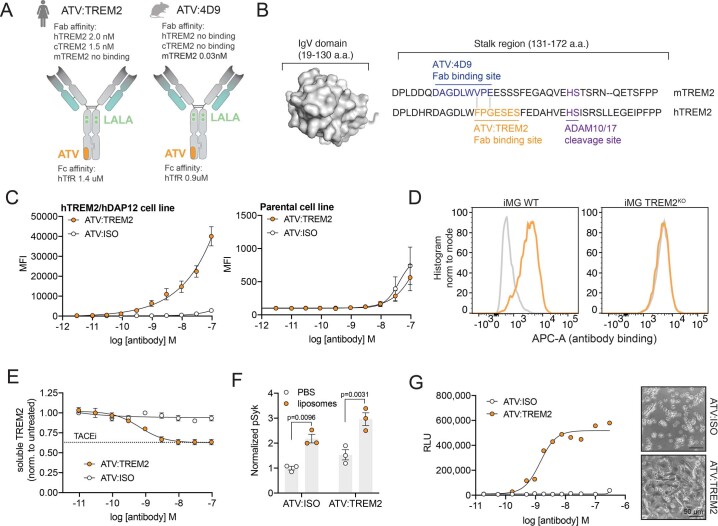
ATV:4D9 and ATV:TREM2 demonstrate similar mechanisms of action with high affinity stalk binding epitopes and cellular function. **(a)** Antibody schematic comparing human specific ATV:TREM2 and mouse specific ATV:4D9 with high affinity TREM2 binding. **(b)** Epitope map of overlapping stalk binding regions for ATV:TREM2 and ATV:4D9 Fabs (space filled model of TREM2 ECD^1^). The binding epitope of ATV:4D9 antibody is located 12-amino acids N-terminal of the ADAM cleavage site at His157. **(c)** FACS analysis of cell binding of ATV:TREM2 to hTREM2-DAP12 HEK293 or parental cells. Endogenous TfR expression on HEK293 cells drives weak binding observed for ATV:ISO and ATV:TREM2 (n = 3 independent experiment, mean ± SEM). (**d**) FACS detection of ATV:TREM2 (100 nM) binding to WT and TREM2 KO iMG with isotype control (ATV:ISO). **(e)** Soluble TREM2 measured in the supernatant of hTREM2-DAP12 HEK293 cells dosed with ATV:TREM2 for 24 h shows dose-dependent reduction of sTREM2 to levels similar to 1 uM GM6001 (n = 3 independent experiment, mean ± SEM). (**f**) ATV:TREM2 and lipid ligands induce pSyk signaling in iMG 24 h post antibody exposure (n = 3 independent experiments, Tukey’s multiple comparisons test, mean ± SEM). **(g)** Human monocytes cultured in limited M-CSF with plate coated ATV:TREM2 or ATV:ISO shows dose-dependent activity of ATV:TREM2 (EC50 0.95 +/− 0.45 nM). Representative data from one out of four human donors are shown.

Next, we sought to generate an antibody with similar properties to ATV:4D9 targeting human TREM2 for the clinic (Extended Data Fig. 3a). Rodents were immunized with the human TREM2 extracellular domain (amino acids 19–172) to enable discovery of a novel human TREM2-activating antibody. This antibody was engineered with an effectorless Fc^41^ (Supplementary Table 1) and ATV^35^ (Fig. 2). Binding affinity of ATV:TREM2 to human TREM2 was 2 nM, whereas binding to human TfR was 1.4 μM, by surface plasmon resonance (SPR) (Fig. 2a and Table 1). ATV:TREM2 binds the TREM2 stalk region, N-terminal to the ADAM17 cleavage site (residues 143–149, FPGESES) determined by hydrogen–deuterium exchange (Extended Data Fig. 3b). ATV:TREM2 has an apparent 4 nM cell binding potency (Extended Data Fig. 3c) and binds endogenous TREM2 on human induced pluripotent stem cell (iPSC)-derived WT microglia (iMG) by fluorescence-activated cell sorting (FACS), whereas *TREM2* knockout (KO) iMG showed no detectable difference compared to isotype control (Extended Data Fig. 3d). Therefore, the human-specific ATV:TREM2 demonstrated similar properties to ATV:4D9 based on high-affinity binding of a stalk region epitope, TREM2 signaling activation, and functional effects in relevant myeloid cells^31^ (Extended Data Fig. 3 and Table 1).

**Table 1:** Comparative TREM2 antibody binding affinity and cellular activity

Assay	Analyte	Anti-TREM2	ATV:TREM2	ATV:4D9
SPR binding K_D_ (M)	hTREM2	2.46 × 10^−9^	2.00 × 10^−9^	No binding
SPR binding K_D_ (M)	msTREM2	No binding	No binding	3.16 × 10^−10^
SPR binding K_D_ (M)	hTfR	No binding	1.43 × 10^−6^	9.09 × 10^−7^
Macrophage survival EC_50_ (M)		2.7 × 10^−9^	4.1 × 10^−9^	4.1 × 10^−9^

SPR binding and macrophage survival potency values for ATV:4D9, anti-TREM2 and ATV:TREM2. Macrophage survival was determined using plate-coated antibody with ATP quantification for cell viability. ATV:4D9-induced survival was tested on bone marrow-derived macrophages (BMDMs) from WT;TfR^mu/hu^ mice.

### ATV:TREM2 reduces shedding and activates TREM2 signaling

To elucidate ATV:TREM2 mechanisms of action, we examined shedding blocking as observed with 4D9 (ref. 31). ATV:TREM2 partially blocked stalk peptide cleavage by ADAM17, whereas the control inhibitor GM6001 completely blocked shedding (Fig. 2b). This biochemical activity is consistent with the ability of ATV:TREM2 to reduce soluble TREM2 in a cell-based assay (Extended Data Fig. 3e) and in the CSF of WT;TfR^mu/hu^ mice treated with ATV:4D9 (Supplementary Fig. 1l).

TREM2 signals upon binding phosphatidylserine (PS) and other anionic lipids^3,10^. Therefore, we determined if antibody binding affects lipid signaling. iMG co-treated with PS liposomes and ATV:TREM2 showed enhanced signaling (Fig. 2c and Extended Data Fig. 3f). To determine if ATV:TREM2 engages a functional cellular response, we examined rescue of survival defects in primary human macrophages cultured in low macrophage colony-stimulating factor (M-CSF) and found a potent survival rescue with a half-maximal effective concentration (EC_50_) of ~1 nM (Extended Data Fig. 3g). Together, these data show that ATV:TREM2 does not block lipid ligands and signals sufficiently to promote cellular function.

### TREM2 receptor clustering is enhanced by ATV TfR binding

Previous studies demonstrated that TREM2-activating antibodies require bivalent binding^31,33^. To test valency properties of ATV:TREM2, we compared bivalent (BV) and monovalent (MV) versions for the impact on pSyk signaling. Whereas anti-TREM2 MV showed no activity (Fig. 2d,e), ATV:TREM2 MV displayed similar activity as anti-TREM2 BV, and ATV:TREM2 BV increased maximal signaling and potency (Fig. 2d,e). Thus, ATV enhances TREM2 Fab activity. These effects required ATV-TfR binding as a competitive TfR antibody inhibited ATV:TREM2ʼs pSyk potentiation (Fig. 2f).

**Extended Data Fig. 4:**
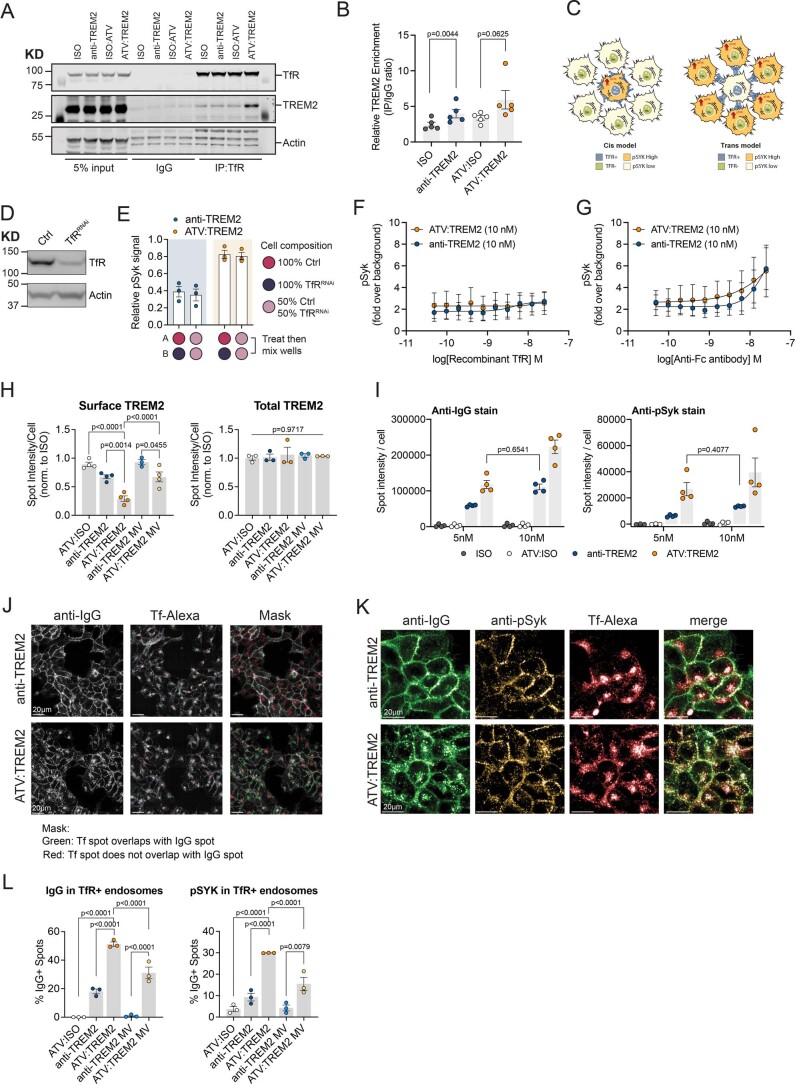
ATV promotes TfR-TREM2 receptor complex formation and internalization and endosomal TREM2 signaling. (**a**) Representative Western blot of co-IP of TREM2 with TfR. hTREM2-DAP12 HEK293 cells were treated with 100 nM ATV:TREM2, anti-TREM2, or isotype controls for 5 min at 37 °C. (**b**) Co-IP quantification of Western blot from (A) (n = 6 independent experiments; two-tailed paired t-test for ISO vs anti-TREM2; two tailed Wilcoxon test ATV:ISO vs ATV:TREM2, mean ± SEM). (**c**) Schematic illustration of *cis-* and *trans-*activation models that could mediate pSyk enhancement by ATV:TREM2. (**d**) Western blot validation of TfR knockdown in the TfR^RNAi^ cell line. (**e**) Cell based cis/trans assay indicates ATV:TREM2 enhances pSyk activity in cis. Relative pSYK signal is expressed as raw pSYK AlphaLisa value normalized to ATV:TREM2 treated control (n = 3 independent experiment, mean ± SEM). **(f)** Normalized pSyk signal measured by AlphaLisa assay. TfR^RNAi^ cells were treated with 10 nM anti-TREM2 or ATV:TREM2 pre-incubated with a dose titration of recombinant TfR protein for 5 min at 37 °C (n = 3 independent experiment, mean ± SEM). (**g**) Normalized pSyk signal detected by AlphaLisa. TfR^RNAi^ cells were treated with 10 nM anti-TREM2 or ATV:TREM2 pre-incubated with a dose titration of a secondary anti-human IgG Fc antibody for 5 min at 37 °C (n = 3 independent experiment, mean ± SEM) **(h)** Immunofluorescence microscopy of hTREM2-DAP12 HEK293 cells demonstrates reduction of surface TREM2 levels with ATV:TREM2 vs anti-TREM2, no changes in total TREM2 levels, consistent with re-distribution of the receptor from the plasma membrane to endosomes (n = 4 independent experiments except for anti-TREM2 MV (n = 3), Tukey’s multiple comparisons test, mean ± SEM). **(i)** hTREM2-DAP12 HEK293 cells dosed with antibody for 10 minutes shows that at similar amounts of bound antibody detected by anti-IgG (representing 5 nM of ATV:TREM2 and 10 nM of anti-TREM2, n = 4 independent experiments, Tukey’s multiple comparisons test, mean ± SEM**(j)** Images depicting masking algorithm used to identify whether TfR-Alexa-647 labeled recycling endosomes (rainbow spots in middle images) either contain (green spots in right-most images) or do not contain (red spots in right-most images) IgG spots (white spots in left-most image) upon dosing with anti-TREM2 (top row) or ATV:TREM2 (bottom row) for 10 minutes with 10 nM antibody. **(k)** Representative images for hTREM2-DAP HEK293 cells dosed with 10 nM antibody for 10 minutes including 20 ug mL^−1^ TfR-Alexa-647, fixed, permeabilized, and stained with anti-IgG and anti-pSyk. IF shows ATV increased colocalization of antibody with pSyk in early endosomes. **(l)** Quantification of percent of IgG or pSyk spots localized within Tf-positive endosomes (n = 3 independent experiments, Tukey’s multiple comparisons test, mean ± SEM).

To investigate receptor interactions, we performed co-immunoprecipitation (co-IP) with a TREM2 antibody and probed for TfR by western blot (Fig. 2g). We found a ~2-fold increase in TfR in the IP fraction isolated from ATV:TREM2 but not anti-TREM2-treated hTREM2-DAP12 HEK293 cells (Fig. 2g,h). A TfR–TREM2 complex was observed in the reciprocal co-IP (Extended Data Fig. 4a,b). Therefore, ATV:TREM2 mediates direct TREM2–TfR interactions. To test TREM2 receptor clustering, we used biotin proximity labeling (Fig. 2i), in which a doxycycline (Dox)-inducible TREM2 fusion protein was tagged intracellularly with BioID2, a mini biotin ligase^42^, and transfected this construct in hTREM2-DAP12 HEK293 cells to generate cells expressing both untagged and BioID-tagged TREM2. ATV:TREM2 drove significant TREM2 biotinylation detected by western blot, compared to anti-TREM2 or isotype (Fig. 2j,k). This provides biochemical evidence that ATV:TREM2 mediates receptor clustering (Fig. 2d,i).

### ATV:TREM2 promotes pSyk via TREM2 and TfR binding in *cis*

Next, we sought to elucidate whether ATV:TREM2 enhances pSyk in *cis* or *trans*. In *cis*, TfR and TREM2 are co-expressed on the same cell; in *trans*, receptor expression is on adjacent cells (Extended Data Fig. 4c). To distinguish between these possibilities, pSyk was detected upon antibody pre-treatment in a mix of TfR^WT^ and TfR^RNAi^ hTREM2-DAP12 HEK293 cells, in which the latter cellsʼ TfR is knocked down with RNAi (Extended Data Fig. 4d). Antibody-treated cells were plated either separately or pre-mixed and then plated. pSyk activity was similar in both conditions, supporting a *cis* mechanism (Extended Data Fig. 4e). Exogenous addition of ATV:TREM2–TfR immuno-complexes with TfR knockdown did not enhance pSyk activity (Extended Data Fig. 4f), in contrast to artificial clustering by a secondary anti-Fc antibody that elevated pSyk (Extended Data Fig. 4g). Taken together, these studies indicate that ATV:TREM2 mediates TfR–TREM2 binding in *cis* to enhance TREM2 signaling.

### ATV-driven internalization results in endosomal signaling

Because TfR undergoes constitutive internalization and endosomal recycling, we hypothesized that ATV could impact intracellular trafficking or subcellular localization of TREM2. We tracked TREM2 by immunofluorescence in ATV-treated hTREM2-DAP12 HEK293 cells and observed rapid surface downregulation (Extended Data Fig. 4h). Total cellular levels remained constant, suggesting that TREM2 is internalized but not degraded (Extended Data Fig. 4h). ATV:TREM2 was detected intracellularly, whereas anti-TREM2 showed surface localization (Fig. 2l). ATV:TREM2 signal intensity was higher than anti-TREM2 (Fig. 2m). ATV:TREM2 BV and MV increased pSyk compared to anti-TREM2 (Fig. 2l,m). With equivalent cellular IgG levels, ATV:TREM2 elicited slightly higher pSyk compared to anti-TREM2 (Extended Data Fig. 4l). These data suggest that ATV:TREM2 enhances TREM2 signaling not solely by increasing bound antibody but likely due to TfR-mediated intracellular trafficking.

To examine this hypothesis, we quantified antibody (IgG) and pSyk co-localization to EEA1^+^ endosomes and TfR^+^ recycling endosomes (Extended Data Fig. 4j). ATV:TREM2 and pSyk were detected in EEA1^+^ and TfR^+^ endosomes approximately two-fold higher than anti-TREM2 (Fig. 2l,n and Extended Data Fig. 4k,l). Therefore, ATV:TREM2 becomes localized to endosomes with pSyk. These studies demonstrate dual mechanisms of action in which ATV enhances TREM2 antibody function via TfR–TREM2 clustering and endosomal signaling. These studies elucidate mechanisms of action for ATV:TREM2 mediated by TfR engagement.

### mTOR and *PLCG2* are required for microglial proliferation

**Fig. 3:**
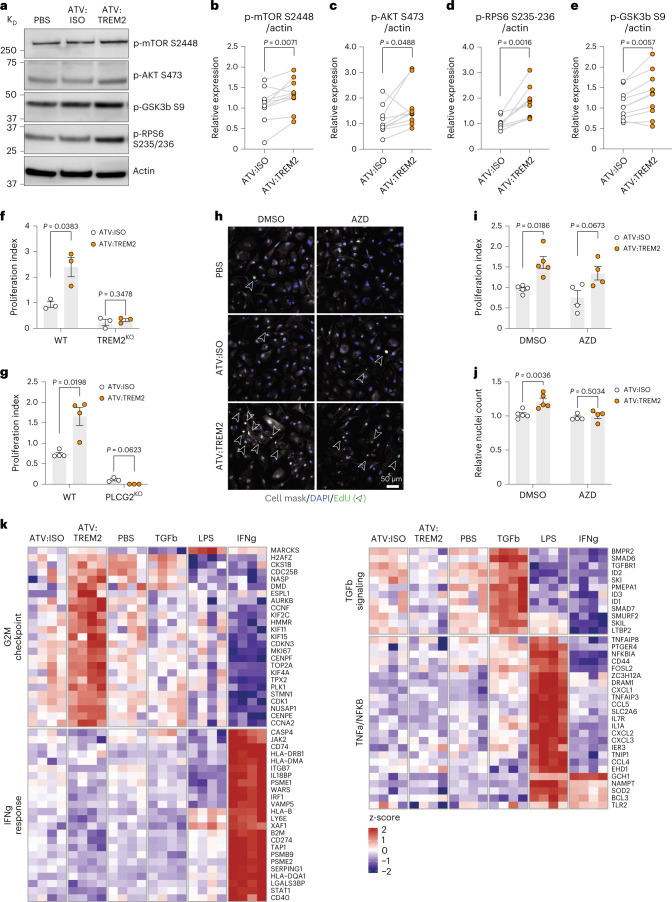
Microglial proliferation induced by ATV:TREM2 requires mTOR signaling and PLCG2. **a**, Representative western blot images for p-mTOR (S2448), pAKT (S473), pGSK3b (S9) and pRPS6 (S235/236) in WT iMG treated with 100 nM ATV:TREM2 or isotype control. **b**–**e**, Quantification of mTOR-S2448 (**b**), AKT-S473 (**c**), RPS6-S235/236 (**d**) and GSK3b-S9 (**e**) phosphorylation levels were normalized to actin. Relative expression was calculated by normalizing to vehicle control (PBS) for each experiment (*n* = 10 independent experiments (**b**–**d**) and *n* = 9 independent experiments (**e**); two-tailed paired *t*-test, mean ± s.e.m.). **f**, ATV:TREM2 induces proliferation in WT iMG but not TREM2 KO iMG. WT or TREM2 KO iMG were treated with 100 nM ATV:TREM2 or isotype control for 96 hours. Forty-eight hours after dose, 20 µM EdU was added to media. The proliferation index was calculated as percentage of EdU^+^ cells normalized to vehicle control (PBS) (*n* = 3 independent experiments; two-tailed multiple-paired *t*-test, mean ± s.e.m.). **g**, Quantification of WT and *PLCG2* KO iMG proliferation. iMG were treated with 100 nM ATV:TREM2 or ATV:ISO (*n* = 4 independent experiments (WT) and *n* = 3 independent experiments (*TREM2* KO); two-tailed multiple-paired *t*-test, mean ± s.e.m.). **h**, Representative images of WT iMG proliferation. Cells were treated with vehicle (DMSO) or mTOR inhibitor AZD8055 (AZD). EdU^+^ iMG are marked with open arrow. **i**, Quantification of WT iMG proliferation treated with mTOR inhibitor AZD8055. 20 nM AZD8055 was co-dosed with 100 nM ATV:TREM2 for 96 hours (*n* = 5 independent experiments (DMSO) and *n* = 4 independent experiments (AZD); two-tailed multiple-paired *t*-test, mean ± s.e.m.). **j**, Nuclei quantification of iMG co-treated with ATV:TREM2 and AZD. Relative nuclei count was normalized to vehicle control (PBS) for each experiment (*n* = 5 independent experiments (DMSO) and *n* = 4 independent experiments (AZD); two-tailed unpaired *t*-test, mean ± s.e.m.). **k**, RNA-seq of iMG treated for 4 days with PBS, 100 nM ATV:ISO or ATV:TREM2 or 10 ng ml^−1^ LPS, 20 ng ml^−1^ TGFβ or 20 ng ml^−1^ IFNγ. Relative expression (z-scores) of the top-most upregulated or downregulated genes selected from pathways of interest. Pathway definitions were taken from the hallmark MSigDB collection; genes shown are a subset of those found in the leading edge of the gene set for each category. Source data

**Extended Data Fig. 5:**
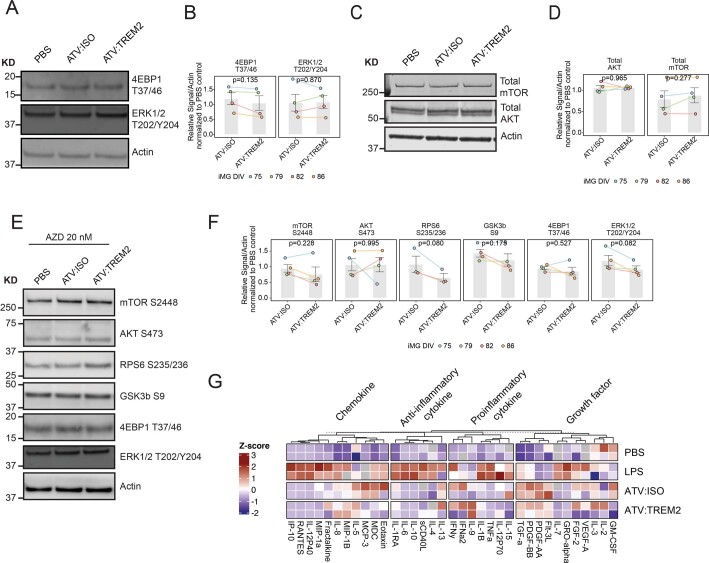
ATV:TREM2 does not promote ERK1/2 phosphorylation or a proinflammatory signature in microglia. (**a**) Representative Western blot images of phosphorylation of 4EBP1 (T37/46) and ERK1/2 (T202/Y204) in WT iMGs treated for 96 h with 100 nM ATV:TREM2 or an isotype control. (**b**) Quantification of p4EBP1 (T37/46) and pERK1/2 (T202/Y204) normalized to actin. Relative expression was calculated by normalizing to PBS vehicle control for each experiment (n = 4 independent experiments, two-tailed paired t-test, mean ± SEM). (**c**) Representative Western blot images of total protein levels of mTOR and AKT in WT iMG treated for 96 h with 100 nM ATV:TREM2 or an isotype control. (**d**) Quantification of total mTOR and AKT protein normalized to actin. Relative expression was calculated by normalizing to PBS control for each experiment (n = 4 independent experiments, two-tailed paired t-test, mean ± SEM). **e**) Representative Western blot images for p-mTOR (S2448), pAKT (S473), pGSK3b (S9) pRPS6 (S235/236), p4EBP1 (T37/46) and pERK1/2 (T202/Y204) showing inhibition of mTOR pathway activation in WT iMG co-treated with 20 nM AZD8055 and 100 nM ATV:TREM2 after 96 h. (**f**) Quantification of phosphorylation targets shown in (E). Phosphorylation signals were normalized to actin. Relative expression was calculated by normalizing to PBS vehicle control for each experiment (n = 4 independent experiments, two-tailed paired t-test, mean ± SEM)). (**g**) Heatmap of human cytokine profiling in supernatant from WT iMG treated with 100 nM ATV:TREM2 for 96 h. Media collected from iMG treated with 10 ng mL^−1^ LPS for 24 h was used for comparison.

Next, we sought to identify signaling pathways by which ATV:TREM2 instructs microglial physiological responses. In human iMG^25,43^, ATV:TREM2 induced phosphorylation of key modulators of the mTOR pathway, including mTOR, AKT, RPS6 and GSK3b, whereas changes in p4EBP1 or pERK1/2 were not detected (Fig. 3a–e and Extended Data Fig. 5a,b). Total protein levels of mTOR and AKT were unaltered (Extended Data Fig. 5c,d). Concurrently, ATV:TREM2 increased proliferation in WT but not *TREM2* KO or *PLCG2* KO iMG (Fig. 3f–h), showing this activity is specific and requires *PLCG2* (refs. 22, 24, 25). The mTOR inhibitor AZD8055 blocked ATV:TREM2-induced mTOR signaling (Extended Data Fig. 5e,f), reduced EdU labeling, and blocked increased iMG cell numbers (Fig. 3h–j). Therefore, ATV:TREM2-driven microglial proliferation requires mTOR signaling.

Next, we found that ATV:TREM2 induced a cell cycle G2M checkpoint gene signature in WT iMG by bulk RNA-seq, distinct pathway biology compared to other stimuli, such as TGFβ, lipopolysaccharide (LPS) or IFNγ (Fig. 3k and Supplementary Table 4). ATV:TREM2-treated WT iMG showed nominal cytokine release compared to LPS (Extended Data Fig. 5g). These data show that ATV:TREM2 promotes microglia proliferation via mTOR and PLCG2 and is biologically distinct from other innate immune stimuli.

### ATV:TREM2 improved pharmacodynamic responses in vivo

**Fig. 4:**
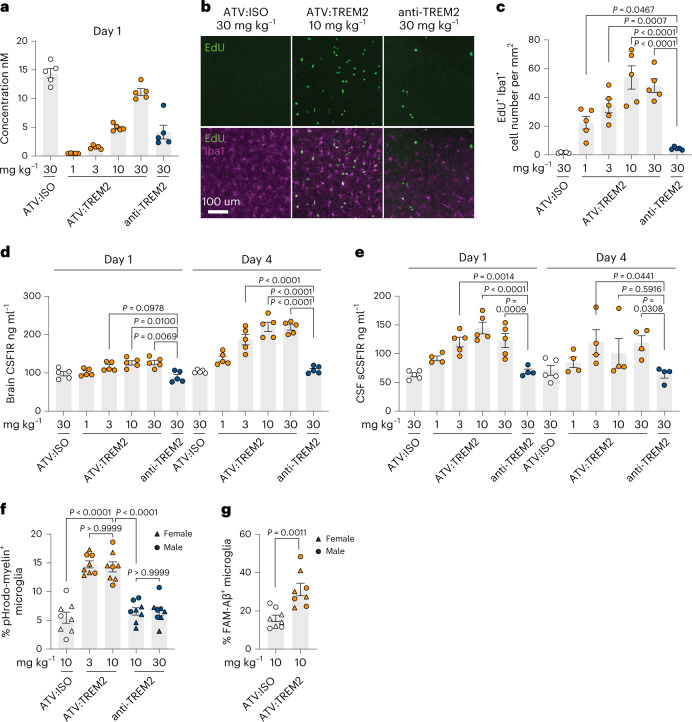
ATV:TREM2 demonstrates improved brain exposure and pharmacodynamic microglial responses compared to anti-TREM2. **a**–**e**, Four-day single-dose study. **a**, ELISA detection of antibodies in whole brain lysates for mice dosed with ATV:TREM2 (1, 3, 10 or 30 mg kg^−1^) and anti-TREM2 (30 mg kg^−1^) 1 day after dose (*n* = 5 mice per group). **b**, Microglia detected by IBA1 staining (purple), and proliferative cells were detected by EdU labeling (green) 4 days after dose. **c**, Quantification of IBA1/EdU staining (*n* = 5 mice per group; Dunnett’s multiple comparisons test, mean ± s.e.m.). **d**, CSF1R detected by ELISA in whole brain lysate (*n* = 5 mice per group; Kruskal–Wallis test for day 1 and Dunnett’s multiple comparisons test for day 4). **e**, CSF1R detected by ELISA in CSF (*n* = 5 mice per group for day 1 (30 mg kg^−1^ ATV:ISO); day 1 (3 mg kg^−1^ ATV:TREM2); day 1 (10 mg kg^−1^ ATV:TREM2); day 1 (30 mg kg^−1^ ATV:TREM2); day 4 (30 mg kg^−1^ ATV:ISO). *n* = 4 mice per group for rest and Dunnett’s multiple comparisons test for day 1; Kruskal–Wallis test for day 4, mean ± s.e.m.). **f**,**g**, Ex vivo microglial phagocytosis for different substrates. Myelin debris (**f**) (*n* = 8 mice per group; Dunnett’s multiple comparisons test) and Aβ (**g**) (*n* = 8 mice per group; two-tailed unpaired *t*-test, mean ± s.e.m.). **a**–**g**, Circle represents male mice, and triangle represents female mice.

**Extended Data Fig. 6:**
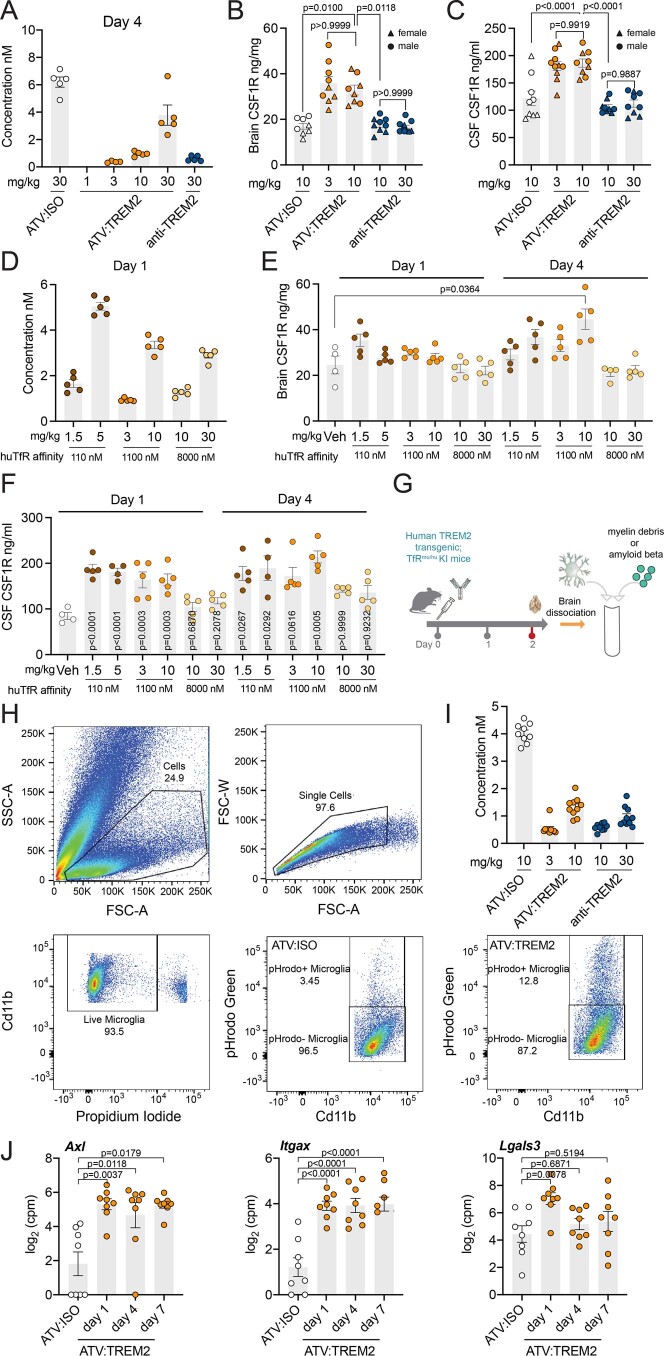
ATV:TREM2 increases phagocytosis and CSF1R in human TREM2 tg;TfR^mu/hu^ mice. **(a)** Antibody levels detected in brain lysates by ELISA shows increased brain exposure of ATV:TREM2 (30 mg kg^−1^) compared anti-TREM2 (30 mg kg^−1^) and comparable brain exposure for ATV:TREM2 at 10 mg kg^-1^ and anti-TREM2 at 30 mg kg^−1^ at day 4 post-dose. (n = 5 mice/group except for ATV:TREM2 3 mg kg^−1^ (n = 4)). **(b)** ATV:TREM2 increases CSF1R in the brain compared to brain exposure matched anti-TREM2 day 2 post dose (n = [8, 9, 8, 9, 10]mice/group, Kruskal-Wallis test, mean ± SEM). (**c**) CSF1R analysis in CSF same as in B. (n = [8, 9, 8, 9, 10]mice/group, Tukey’s multiple comparisons test, mean ± SEM) (**b**-**c**) Circles represents male mice and triangle represents female mice. **(d)** Detection of antibody concentrations show matched brain exposure of three ATV:TREM2 molecules with different ATV affinities 1-day post-dose (n = 5 mice/group). **(e-f)** High and mid-affinity ATV:TREM2 molecules induce comparable increase of CSF1R in the brain (**e)** n = 5 mice/group except for Vehicle (Veh) and day 4 10 mg kg^−1^ 8,000 nM (n = 4, Kruskal-Wallis test, compared to vehicle group**)** and CSF **(f)** (n = 5 mice/group, except for Veh and day 4 5 mg kg^−1^ 110 nM n = 4), Dunnett’s multiple comparisons test for day 1, Kruskal-Wallis test for day 4) day 1 and 4 post dose whereas low-affinity ATV:TREM2 induces a weak elevation of CSF1R in CSF at day 4 (n = 4-5 mice/group, mean ± SEM). **(g)** Schematic of experimental approach to evaluate in vivo dosed antibody impact to microglial phagocytosis ex vivo. A single dose of ATV:TREM2, anti-TREM2, or ATV:ISO was administrated to human TREM2 tg; TfR^mu/hu^ KI mice and brain microglia were isolated 2 days post dose for ex vivo myelin phagocytosis analysis. The same method was used to assess amyloid phagocytosis. **(h)** FACS gating strategy to quantify pHrodo-myelin positive microglia. After treatment with pHrodo-green myelin and staining, cell suspensions containing microglia were analyzed using BD FACS Aria III. Single cells were separated from debris by FSC and SSC characteristics. Live microglia were identified as a population of CD11b^+^ and propidium iodide^negative^ cells. pHrodo-myelin uptake was then quantified in 20,000 microglia recorded from each sample. **(i)** Antibody concentrations were detected in brain lysates by ELISA which shows comparable levels of ATV:TREM2 and anti-TREM2 day 2 post dose (n = [9, 10, 10, 9, 10] mice/group). **(j)** ATV:TREM2 induces transcription of genes associated with phagocytosis*. Axl, Itgax, Lgals3* mRNA levels were detected in isolated brain microglia after peripheral administration of 10 mg kg^−1^ ATV:TREM2 1-, 4-, and 7-days post dose. Graphs represent bulk mRNA measured by RNA-seq. Data shown as log_2_ counts per million (with a pseudocount of 1 added) in each biological replicate (n = 8 mice/group, each group compared to ATV:ISO group, Kruskal-Wallis test for Axl, Dunnett’s multiple comparisons for *Itgax* and *Lgals3*, mean ± SEM).

To explore if our in vitro iMG findings translated in vivo, we generated a *TREM2* BAC transgenic (tg) mouse model expressing human TREM2 (Supplementary Fig. 3a–c). This model was bred to WT;TfR^mu/hu^ mice for pharmacokinetic and pharmacodynamic studies. Brain concentrations of ATV:TREM2 increased dose dependently and were higher than anti-TREM2 (Fig. 4a and Extended Data Fig. 6a), demonstrating that ATV:TREM2 has improved brain exposure after a single IV dose. Using IBA1 immunostainings coupled with EdU labeling to detect proliferating microglia, we found that ATV:TREM2 induces a dose-dependent increase in IBA1^+^ and IBA1^+^ EdU^+^ microglia, whereas anti-TREM2 showed no effect (Fig. 4b,c). These results indicate that ATV:TREM2 enhances microglia proliferation in vivo compared to anti-TREM2 at similar brain exposures.

CSF1R is expressed on microglia and is essential for survival^44^. A TREM2 antibody elevated soluble CSF1R (sCSF1R) in CSF of healthy volunteers^32^; therefore, it represents a potential pharmacodynamic biomarker. ATV:TREM2, but not anti-TREM2, increased brain and CSF CSF1R levels in a dose-dependent manner, with maximal response at 10 mg kg^−1^ (Fig. 4d,e). As TREM2 can affect microglia in a sex-dependent manner^45^, we confirmed that ATV:TREM2 increased CSF1R in males and females (Extended Data Fig. 6b,c and Supplementary Table 3), showing no significant sex differences.

To elucidate effects of ATV affinity, two additional variants were engineered: one with higher TfR affinity (110 nM) and one with lower affinity (8,000 nM), compared to the 1,100 nM TfR affinity used in all other studies. WT;human TREM2 tg;TfR^mu/hu^ mice were treated with variable doses to achieve similar brain exposure (Extended Data Fig. 6d). ATV^8000*n*M^:TREM2 reached similar brain concentrations as ATV^1100*n*M^:TREM2 but did not induce CSF1R (Extended Data Fig. 6e,f), whereas ATV^110*n*M^:TREM2 induced similar CSF1R levels as ATV^1100*n*M^:TREM2, suggesting that ATV requires an affinity threshold to enhance TREM2 Fab activity.

### ATV:TREM2 enhances microglial phagocytosis ex vivo

Previous studies demonstrated that TREM2 antibodies enhance microglial phagocytosis in vitro^31,34^. To determine the impact of ATV:TREM2 on this function, we administered antibodies in vivo and then evaluated microglial uptake of myelin or Aβ ex vivo. WT;human TREM2 tg;TfR^mu/hu^ mice were IV dosed with isotype control, ATV:TREM2 or anti-TREM2; brain microglia were harvested 2 days later, incubated with fluorescently labeled substrates (Extended Data Fig. 6g); and phagocytosis was quantified by FACS (Extended Data Fig. 6h). ATV:TREM2 increased phagocytosis of pHrodo-green^+^ myelin and FAM-labeled Aβ compared to isotype control (Fig. 4f,g). In contrast, anti-TREM2 at similar brain concentrations (Extended Data Fig. 6l) did not alter microglial myelin uptake (Fig. 4f). Moreover, ATV:TREM2 upregulated genes associated with phagocytosis, such as *Axl*, *Itgax* and *Lgals3* (Extended Data Fig. 6j).

### ATV:TREM2 demonstrates enhanced brain uptake and catabolism

**Extended Data Fig. 7:**
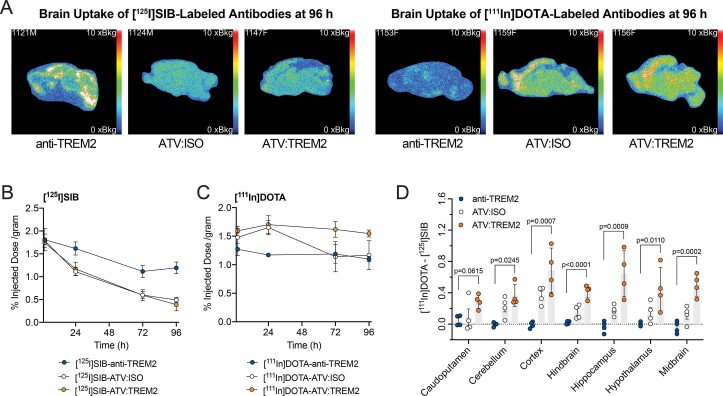
Single photon emission computed tomography imaging demonstrates increased ATV:TREM2 biodistribution and catabolism in brain. **(a)** Representative autoradiography images of sagittal brain sections from WT; hTREM2 tg; TfR^mu/hu^ mice 96 h after administration of ATV:TREM2, anti-TREM2, and ATV:ISO radiolabeled with [^111^In]DOTA or [^125^I]SIB. **(b,c)** Longitudinal SPECT/CT imaging quantification of whole-brain uptake of single dose (100 µCi, 200 µL, 1.5 mg kg^−1^, IV) ATV:TREM2, anti-TREM2, and ATV:ISO, radiolabeled with [^125^I]SIB **(b)** or [^111^In]DOTA **(c)** in WT; hTREM2 tg; TfR^mu/hu^ mice. Whole brain %ID/g was corrected for contribution from cerebral blood volume. Data are represented as mean +/− SEM (n = 3 mice/group). **(d)** Percent of catabolized ATV:TREM2 in several brain regions 96 h after single dose exceeds that of ATV:ISO control. [^111^In]DOTA and [^125^I]SIB signal was quantified by *ex vivo* gamma counting in resected brain regions, and percent of catabolized antibody was estimated by subtracting %ID/g for [^125^I]SIB signal from that of [^111^In]DOTA signal (n = 4 mice/group, one-way ANOVA with Dunnett’s multiple comparison test for each region, except for cerebellum which applied Brown-Forsythe and Welch ANOVA tests, mean ± SEM).

We next determined the kinetics of uptake and catabolism of ATV:TREM2 using longitudinal single-photon emission computed tomography (SPECT) imaging with radiolabeled antibodies. Radiometabolites generated from [^125^I]SIB-labeled antibodies are rapidly effluxed, whereas those from [^111^In]DOTA labeling have residualizing properties with cumulative uptake due to cellular retention^46^. [^125^I]SIB-labeled ATV:TREM2 and ATV:ISO showed substantial clearance from brain (Extended Data Fig. 7a,b). In contrast, [^111^In]DOTA-labeled ATV:TREM2 showed a persistent elevated signal over the 96-hour time course (Extended Data Fig. 7a,c).

ATV:TREM2 showed a high catabolic rate in all brain regions examined (Extended Data Fig. 7d), providing a mechanistic explanation for lower brain concentrations of ATV:TREM2 compared to ATV:ISO control observed in multiple studies (Fig. 1k and Extended Data Fig. 6a,l). Notably, SPECT imaging showed that ATV:TREM2 significantly improved brain biodistribution compared to anti-TREM2. Taken together, these results demonstrate that ATV:TREM2 is broadly distributed throughout the brain, and acute TREM2 activation is sufficient to mediate sustained microglial responses, as effects persist after antibody clearance.

We assessed peripheral biodistribution and systemic safety in WT;TfR^mu/hu^ mice because TfR is broadly expressed, and TREM2 is present on myeloid cells. We observed higher concentrations of ATV:ISO and ATV:4D9 relative to 4D9 in tissues with high TfR, such as brain and bone marrow (Supplementary Fig. 2a,b). Similar biodistribution was observed across all three treatment groups in spleen, plasma and lung (Supplementary Fig. 2c–e). Overall, these data suggest that ATV:4D9 biodistribution is largely driven by TfR rather than TREM2 binding. No histopathological findings, including no changes to immune cells, were observed in the bone, bone marrow, brain, heart, kidney, liver, lung, lymph node, spleen, intestine, thymus and sciatic nerve when compared to vehicle control in a 12-week study of ATV:4D9 in WT;TfR^mu/hu^ mice dosed weekly at 10 mg kg^−1^.

### ATV:TREM2 enhances mitochondrial metabolism in microglia

**Fig. 5:**
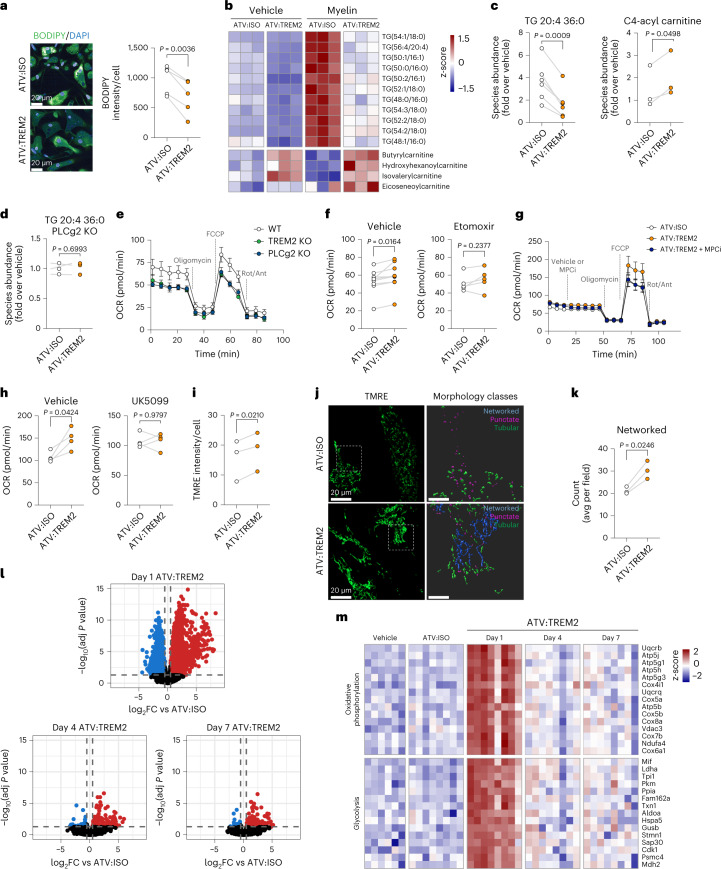
ATV:TREM2 drives metabolism through mitochondrial fatty acid oxidation and *PLCG2*-dependent respiration in microglia. **a**, Representative microscopy images from iMG treated with 10 µM oleic acid and then 100 nM ATV:ISO or ATV:TREM2 for 48 hours. BODIPY fluorescence (shown in green) was quantified (*n* = 5 independent experiments; two-tailed paired *t*-test, mean ± s.e.m.). **b**, Heat map of LC–MS analysis for TG species and acyl carnitines modulated by ATV:TREM2 in iMG treated with myelin. Plotted values are log_2_-transformed raw counts and scaled by row. **c**,**d**, ATV:TREM2 reduces TGs and increases acyl carnitines in WT iMG treated with myelin (**c**) (*n* = 6 independent experiments (TG) and *n* = 3 independent experiments (acyl carnitines); two-tailed paired *t*-test, mean ± s.e.m.) but not *PLCG2* KO iMG treated with myelin (**d**) (*n* = 3 independent experiments; two-tailed paired *t*-test, mean ± s.e.m.). **e**, Seahorse fatty acid oxidation OCR respiration measurements in *TREM2* KO and *PLCG2* KO iMG (*n* = 3 independent experiments; mean ± s.e.m.). **f**, ATV:TREM2 increases maximal respiration in WT iMG detected with Seahorse fatty acid oxidation OCR measurements (*n* = 7 independent experiments; two-tailed paired *t*-test, mean ± s.e.m.). The CPT-1 inhibitor etomoxir blocks the effect of ATV:TREM2 on respiration (*n* = 5 independent experiments; two-tailed paired *t*-test, mean ± s.e.m.). **g**,**h**, Seahorse analysis for glucose oxidation. ATV:TREM2 was treated at 100 nM for 3 days. The ATV:TREM2 effect is blocked by an MPC inhibitor, UK5099 (*n* = 4 independent experiments; two-tailed paired *t*-test, mean ± s.e.m.). **i**, ATV:TREM2 increases average TMRE intensity in iMG after 3 days with 100 nM antibody (*n* = 6 independent experiments; two-tailed paired *t*-test, mean ± s.e.m.). **j**, Representative images of super-resolution microscopy of TMRE staining in iMG. Mitochondria were segmented into networked and punctate morphologies. **k**, Morphometric analysis of the prevalence of networked mitochondria (*n* = 3 independent experiments, two-tailed paired *t*-test). **l**, Volcano plots of RNA-seq analysis of microglia isolated from mice dosed with 10 mg kg^−1^ of ATV:ISO or ATV:TREM2 for 1 day, 4 days or 7 days. Red or blue indicate significantly upregulated or downregulated genes, respectively. The *x* axis represents log_2_ fold change in expression compared to vehicle-treated mice, and the y axis represents –log_10_ adjusted *P* value. **m**, Relative expression (z-scores) of the top-most upregulated or downregulated genes at day 1 after dose selected from oxidative phosphorylation and glycolysis pathways. OCR, oxygen consumption rate.

**Extended Data Fig. 8:**
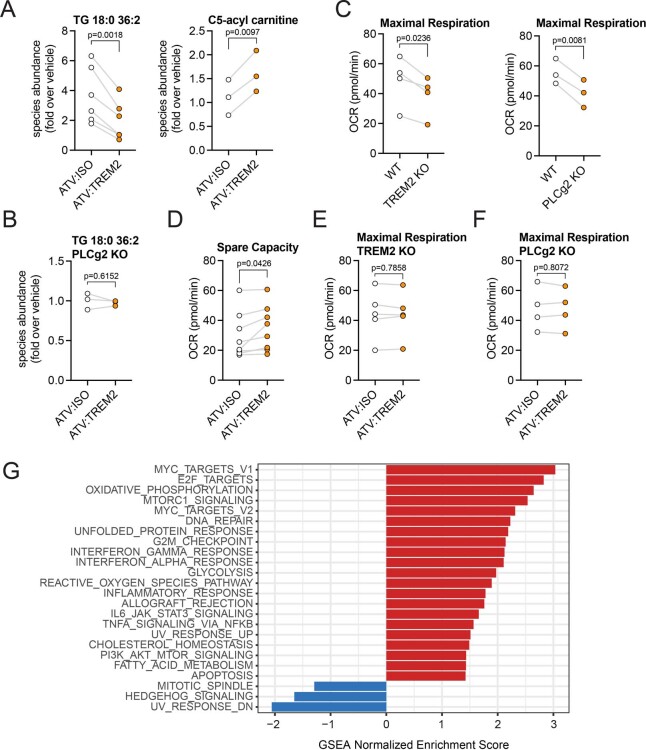
ATV:TREM2 increases microglial metabolism in a *TREM2* and *PLCG2*-dependent manner. **(a)** Additional species of triglycerides (TG) and short chain carnitines modulated in iMG with ATV:TREM2 treatment (n = 3-5 independent experiment, two-tailed paired t-test, mean ± SEM). **(b)** ATV:TREM2 does not modulate TG in *PLCG2* KO iMG (n = 3 independent experiment, two-tailed paired t-test, mean ± SEM). **(c)** Maximal respiration measured by Seahorse fatty acid oxidation kit is reduced in both *TREM2* KO and *PLCG2* KO iMG (n = 3-4 independent experiment, two-tailed paired t-test, mean ± SEM). **(d)** ATV:TREM2 increased spare capacity measured by Seahorse fatty acid oxidation kit (n = 8 independent experiment, two-tailed paired t-test, mean ± SEM). **(e)** ATV:TREM2 does not modulate maximal respiration or spare capacity in *TREM2* KO iMG (n = 4 independent experiment, two-tailed paired t-test, mean ± SEM). **(f)** ATV:TREM2 does not modulate maximal respiration and spare capacity *PLCG2* KO iMG (n = 4 independent experiment, two-tailed paired t-test, mean ± SEM). **(g)** RNAseq analysis of brain microglia isolated from hTREM2 tg; TfR^mu/hu^ mice dosed with 10 mg kg^−1^ ATV:TREM2. GSEA for top pathways based on a p-value cutoff of 0.05 for up- or downregulated gene sets 1 day post ATV:TREM2 dose.

We then sought to elucidate the ability of ATV:TREM2 to modulate disease-relevant microglial functions. Previous studies demonstrated that *TREM2* KO microglia are defective in clearing lipid-rich myelin debris^10,25,47^; therefore, we quantified lipid droplets by BODIPY staining in WT iMG treated with purified myelin followed by ATV:TREM2 and found reduced BODIPY staining compared to an isotype control (Fig. 5a). We next used liquid chromatography–mass spectrometry (LC–MS) to resolve lipid species and observed significant reductions in several triglycerides (TGs) (Fig. 5b,c and Extended Data Fig. 8a), a known component of lipid droplets^48,49^. Additionally, numerous acyl carnitine species were increased (Fig. 5b,c and Extended Data Fig. 8a). ATV:TREM2-mediated lipid catabolism required PLCγ2, as no effects on TGs were observed in *PLCG2* KO iMG (Fig. 5d and Extended Data Fig. 8b), further indicating that PLCγ2 acts downstream of TREM2 for lipid metabolism^25^.

Because ATV:TREM2 concomitantly reduced TGs and increased acyl carnitines, a byproduct of mitochondrial fatty acid metabolism^50^, ATV:TREM2 may promote mitochondrial function. Notably, both *TREM2* KO and *PLCG2* KO iMG showed impaired mitochondrial respiration under substrate-limiting conditions to isolate fatty acid metabolism (Fig. 5e and Extended Data Fig. 8c). Conversely, ATV:TREM2 increased maximal respiration and spare capacity (Fig. 5f and Extended Data Fig. 8d), demonstrating that ATV:TREM2 imparts metabolic flexibility and increased energetic capability in iMG. TREM2 and PLCγ2 were required for this activity (Extended Data Fig. 8e,f). The carnitine palmitoyltransferase 1 (CPT-1) inhibitor etomoxir blocked ATV:TREM2-driven increased respiration (Fig. 5f), suggesting that CPT-1, an enzyme that mediates acylation of free fatty acids and translocation into the mitochondria, is also required. ATV:TREM2 thus improves mitochondrial metabolism via fatty acid oxidation, consistent with its ability to clear TGs stored in lipid droplets. ATV:TREM2 also strongly increased mitochondrial oxidation of glucose, which was blocked by the mitochondrial pyruvate carrier (MPC) inhibitor UK5099 (Fig. 5g,h). Together, these results indicate that ATV:TREM2 increases the energetic capacity of microglia by promoting fatty acid oxidation and aerobic respiration via glucose catabolism.

To further investigate the effects of ATV:TREM2 on mitochondria, we assessed membrane potential with tetramethylrhodamine ethyl ester (TMRE) staining and found that ATV:TREM2 increased TMRE intensity by high-content imaging in iMG (Fig. 5i,j). To determine whether mitochondrial structure is impacted by ATV:TREM2, we performed super-resolution microscopy, which revealed increased networked mitochondria morphology (Fig. 5j,k). Importantly, networked mitochondria have been shown to correlate with increased oxidative phosphorylation and catabolism^51,52^, consistent with ATV:TREM2ʼs ability to boost microglial respiration.

We next examined whether this metabolic activity could be detected in vivo, so we performed RNA-seq on microglia isolated from WT;hTREM2 tg;TfR^mu/hu^ mice dosed with 10 mg kg^−1^ of ATV:TREM2, ATV:ISO or vehicle control. ATV:TREM2 upregulated a significant transcriptional response at 1 day compared to isotype or vehicle, which was attenuated by day 4 (Fig. 5l). Strikingly, ATV:TREM2 increased oxidative phosphorylation and glycolysis pathway genes (Fig. 5m), consistent with increased metabolic functions in iMG. The top pathways regulated by ATV:TREM2 are shown in Extended Data Fig. 8g, and a complete gene list is provided in Supplementary Table 4.

### ATV:TREM2 increases brain microglia activity in an AD model

To investigate the effects of ATV:TREM2 using brain imaging, we crossed the hTREM2 tg;TfR^mu/hu^ mice to the 5×FAD model^53^ and monitored brain microglial activity by [^18^F]GE-180 18-kDa translocator protein positron emission tomography (TSPO-PET). Increased mitochondrial TSPO expression is associated with responsive microglia^54^, and TREM2 LOF brains exhibit lower TSPO-PET signal compared to WT controls^55,56^. Therefore, TSPO-PET imaging could be an indicator of ATV:TREM2 function in vivo.

**Fig. 6:**
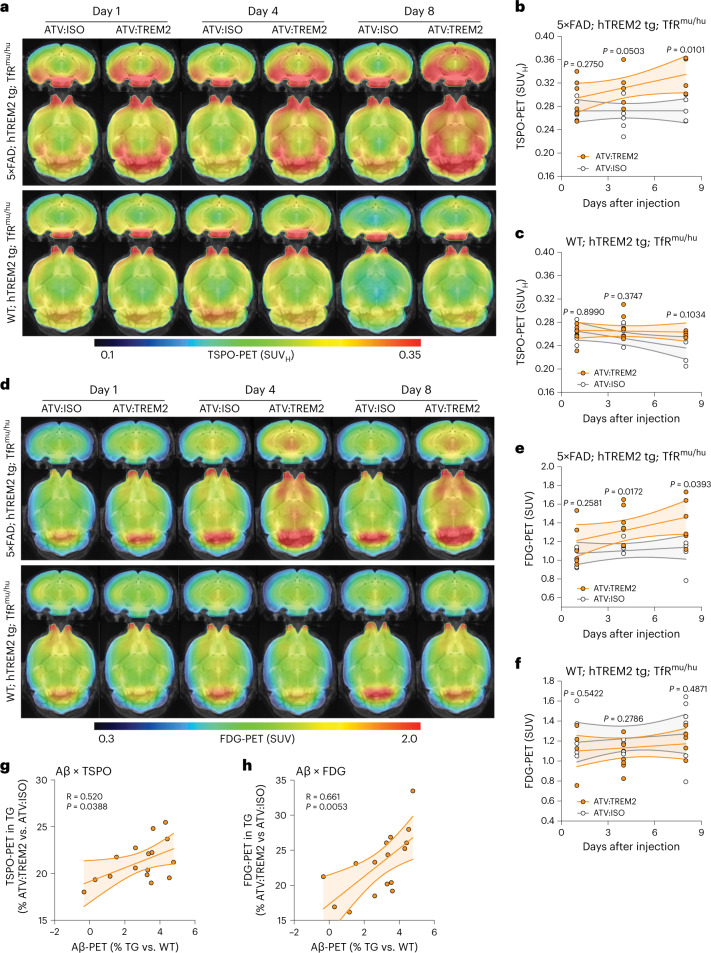
ATV:TREM2 increases brain microglial activity and glucose metabolism in an AD model. **a**, Coronal and axial slices show cold scaled group average images of TSPO-PET (SUV_H_) projected upon a standard MRI T1-weighted atlas from 5×FAD;hTREM2 tg;TfR^mu/hu^ mice (top row) or WT;hTREM2 tg;TfR^mu/hu^ mice (bottom row) mice dosed with 10 mg kg^−1^ of antibody. **b**,**c**, Quantification of TSPO-PET 1, 4, and 8 days after dose in 5×FAD;hTREM2 tg;TfR^mu/hu^ mice (**b**) and WT;hTREM2 tg;TfR^mu/hu^ mice (**c**). Scatter plot of individual TSPO-PET (SUV_H_) values. Dotted lines represent linear associations between interval after antibody dosing and TSPO-PET quantification per group and with a 95% confidence interval (*n* = 6 mice per group; two-tailed unpaired *t*-test for each timepoint, except for day 8, which used the two-tailed unpaired *t*-test with Welch’s correction). **d**, Coronal and axial slices show cold scaled group average images of FDG (SUV) projected upon a standard MRI T1-weighted atlas from 5×FAD;hTREM2 tg;TfR^mu/hu^ mice (top row) or WT;hTREM2 tg;TfR^mu/hu^ mice (bottom row) after 10 mg kg^−1^ of antibody. **e**,**f**, Quantification of cortical glucose uptake measured by FDG-PET 1, 4, and 8 days after dose of ATV:ISO or ATV:TREM2 for 5×FAD;hTREM2 tg;TfR^mu/hu^ mice (**e**) and WT;hTREM2 tg;TfR^mu/hu^ mice (**f**). Scatter plot of individual FDG (SUV) values. Dotted lines represent linear associations between interval after antibody dosing and FDG-PET quantification per group with a 95% confidence interval (*n* = 6 mice per group; two-tailed unpaired *t*-test for each timepoint). **g**,**h**, Regional correlation of biomarker alterations (5×FAD;hTREM2 tg;TfR^mu/hu^ versus WT;hTREM2 tg;TfR^mu/hu^ mice) between FBB-PET at 5 months and TSPO-PET (SUV_H_) (**g**) and FDG-PET (SUV) (**h**) at the group level.

TSPO-PET was assessed in 5×FAD;hTREM2 tg;TfR^mu/hu^ and WT;hTREM2 tg;TfR^mu/hu^ mice dosed with ATV:TREM2 and an isotype control at 1, 4, and 8 days after dose. ATV:TREM2 increased cortical TSPO-PET signal in 5×FAD;hTREM2 tg;TfR^mu/hu^ with significant differences at day 8 compared to an isotype control (Fig. 6a,b). TSPO signal in WT;hTREM2 tg;TfR^mu/hu^ mice was detected at a lower baseline compared to 5×FAD. In WT mice, ATV:TREM2 treatment resulted in a small but statistically significant increase in TSPO signal intensity at day 8 compared to the isotype control (Fig. 6a,c). Therefore, ATV:TREM2 increased mitochondrial TSPO, indicating elevated microglial activity in vivo.

### ATV:TREM2 increases brain glucose metabolism in an AD model

Brain glucose hypometabolism is an early feature of AD^57^ and is associated with more rapid cognitive decline^58^. Microglial activation state can contribute to glucose metabolism in the brain detected by FDG-PET imaging^54^. Additionally, TREM2 LOF reduced brain glucose metabolism in aged and amyloid mouse models^54–56^, implicating TREM2 in regulating brain metabolic activity. Therefore, we hypothesized that ATV:TREM2 could impact brain glucose metabolism. Cortical FDG-PET imaging showed that ATV:TREM2 treatment increased FDG-PET signal compared to isotype control in 5×FAD;hTREM2 tg;TfR^mu/hu^ mice (day 1: +15%, day 4: +19%, day 8: +27%), whereas no effect was observed in WT;hTREM2 tg;TfR^mu/hu^ mice (day 1: −5%, day 4: −3%, day 8: −3%) (Fig. 6d,f). Notably, TfR protein levels were similar in WT and AD mouse models (Supplementary Fig. 4a), suggesting that differences in TfR expression do not contribute PET imaging findings. Human TREM2 levels were elevated in sorted microglia from 5×FAD mice compared to WT, as expected^11,59^ (Supplementary Fig. 4b).

We performed [^18^F]florbetaben beta-amyloid-PET in 5×FAD;hTREM2 tg;TfR^mu/hu^ mice to examine if regional amyloid load correlated with ATV:TREM2 effects on TSPO-PET and FDG-PET. Given the short duration of these studies, we did not anticipate changes in amyloid plaque load; however, there was significant regional association for TSPO-PET and FDG-PET at day 8 with frequency band broadening (FBB) signal, suggesting that ATV:TREM2 impacts brain regions based on amyloid load (Fig. 6g,h). These results suggest that ATV:TREM2 could rescue deficits in brain glucose metabolism, potentially ameliorating this metabolic deficit found in AD.

## Discussion

In this study, we describe the mechanisms of action and cellular functions of ATV-enabled anti-TREM2 biologics, including mouse-specific ATV:4D9 and human-specific ATV:TREM2. Our findings demonstrate that ATV enables improved biodistribution and activity of TREM2 antibodies. The functional genetics demonstrate that LOF TREM2 LOAD risk variants disable protective microglial responses. Therefore, promoting TREM2 to improve microglia functions is a compelling therapeutic approach. Surprisingly, ATV not only improved brain uptake and biodistribution of our TREM2 antibody; it also enhanced TREM2 signaling at the cellular level through co-engagement of TREM2 and TfR. We show that a key physiological effect of ATV:TREM2 is to improve the energetic capacity of microglia through enhanced fatty acid and glucose oxidation in mitochondria. We also demonstrate that ATV:TREM2 increased microglial activity and glucose metabolism in an amyloid mouse model via TSPO-PET and FDG-PET imaging, respectively.

The concept and generation of ATV:TREM2 was driven by the need to improve target engagement in the brain. Although the transport vehicle (TV) platform improved brain uptake for ATV:TREM2 as observed with other large molecules^35,36,60^, the impact of ATV on the cellular activity of a TREM2 antibody remained an open question. We found that ATV altered TREM2 Fab function through two distinct mechanisms: (1) enhanced receptor clustering and (2) increased endosomal signaling. Because ATV:TREM2 resulted in enhanced microglia responses compared to anti-TREM2 with equivalent brain IgG concentrations, the molecular and cellular mechanisms identified in vitro are likely engaged in vivo. These observations indicate that ATV:TREM2 has superior activity in addition to enhanced brain delivery and is, therefore, a highly differentiated candidate biotherapeutic.

Previous work showed that a TREM2 antibody can activate AKT^34^; however, the cellular functions it mediates had not been demonstrated. We found that ATV:TREM2 activates mTOR signaling, which is required for microglia proliferation. Furthermore, we demonstrate that PLCγ2 is required for proliferation and metabolic pathways downstream of TREM2 in iMG. *PLCG2* KO phenocopies *TREM2* KO in iMG, showing deficient proliferation and mitochondrial respiration. These findings support a genetically validated TREM2 pathway in which PLCγ2 acts downstream and is required for diverse microglial functions induced by ATV:TREM2. Our studies provide further support for the direction of therapeutic modulation of TREM2, because an AD-protective *PLCG2* variant has been shown to be mildly hypermorphic, indicating that increased PLCγ2 activity is beneficial for disease^23,25^. Our findings indicate microglia functions with disease-modifying potential based on the link to additional AD-relevant genes.

The functional implications of human AD genetics that point to TREM2 in microglia as a modulator of disease risk remain a critical question in the field. Previous studies conducted 48 hours after TREM2 antibody dosing^32,33^ show that pharmacological activation elicited transcriptional responses. However, these data had not shown specific functional effects on microglia. We show direct evidence of microglia proliferation induced by ATV:TREM2, which could help resolve pathology or serve as a renewal process to reset microglial state. The proliferative response appears limited to a subset of microglia, and factors regulating it are not yet understood. ATV:TREM2 also improved microglial phagocytosis, suggesting that ATV:TREM2 imparts multiple functions that could ameliorate disease.

A key advancement of our studies is the temporal and dose–response effects of ATV:TREM2 in vivo. Whereas previous studies evaluated TREM2 antibodies at one timepoint with a single dose^32,33^, here we provide a more comprehensive view. ATV:4D9 induced durable yet reversible microglial responses by single-cell transcriptional profiling and morphometry. The ability of microglia to reverse states has recently been observed where TREM2 antagonistic antibodies reduce *Grn* LOF hyperactivation^61^, indicating that microglial states are indeed tunable.

Known features of TREM2 LOF microglia include survival defects and arrest in a homeostatic low metabolic state^56,62,63^ and lipid accumulation due to myelin debris^10,25^. Therefore, we sought to determine whether TREM2-related metabolic deficiencies were a secondary consequence of survival deficits or if cellular metabolism could be specifically activated by ATV:TREM2. We discovered that ATV:TREM2 promotes energetic capacity via specific metabolic pathways. Namely, ATV:TREM2 promoted mitochondrial fatty acid oxidization, in which TGs stored in lipid droplets are catabolized to increase microglial respiration. ATV:TREM2 also promoted glucose oxidation, demonstrating flexibility in substrate utilization. We hypothesized that this in vitro activity could connect to disease efficacy rationale as patients with AD and TREM2 LOF mice display hypometabolism with low FDG-PET signal^54–56,58^. ATV:TREM2 increased FDG-PET in an amyloid mouse model, thereby improving brain metabolism in a disease context. Whether ATV:TREM2 induces glucose uptake cell autonomously in microglia or influences other CNS cell types remains an open question. Taken together, our results demonstrating improved brain exposure and biodistribution of a TREM2-activating antibody capable of enhancing microglial functions and improving brain metabolism suggest that ATV:TREM2 is a differentiated therapeutic candidate for the treatment of AD.

## Methods

### Mouse lines and animal care

The genotype, age and sex information of mouse models used in this study are summarized in Supplementary Table 2. TfR^mu/hu^ mouse line was developed in-house by humanizing the apical domain of mouse TfR^35,36^. The chimeric allele is referred to as TfR^mu/hu^. *App*^*SAA*^ mouse model^38^ was crossed to TfR^mu/hu^ mice to generate *App*^SAA^;TfR^mu/hu^ mice (homozygous;homozygous). The human Trem2 BAC tg mouse model was generated at Denali Therapeutics by the introduction of engineered BAC DNA CTD-2210D2 into the mouse genome (see the detailed description in the Supplementary Methods). 5×FAD mice (B6SJLTg(APPSwFlLon,PSEN1*M146L*L286V)6799Vas/Mmjax) were purchased from the Jackson Laboratory (MMRRC strain no. 034840-JAX)^53^. 5×FAD mice, human TREM2 BAC tg mice and TfR^mu/hu^ mice were crossed to generate 5×FAD;hTREM2 tg;TfR^mu/hu^ (hemizygous;hemizygous;homozygous) and WT;hTREM2 tg;TfR^mu/hu^ mice (non-carrier;hemizygous;homozygous). Mice are maintained on a C57BL/6J genetic background. Mouse husbandry and experimental procedures were approved by the Denali Institutional Animal Care and Use Committee. All animal experiments at the German Center for Neurodegenerative Diseases (DZNE) were performed in accordance with animal handling laws of the state of Bavaria (Germany). Housing conditions included standard pellet food and water provided ad libitum, a 12-hour light/dark cycle at a temperature of 22 °C with maximal five mice per cage and cage replacement once per week with regular health monitoring.

### RNA-seq

iMG RNA was extracted with the RNeasy Plus Micro Kit (Qiagen, 74034) to be used as input for bulk RNA-seq library generation using the QuantSeq 3′ mRNA-seq Library Prep Kit FWD for Illumina (Lexogen, A01173) with the unique molecular identifier (UMI) second-strand synthesis add-on module. Libraries were pooled and shipped to SeqMatic for sequencing on an Illumina sequencer.

scRNA-seq for WT mice was prepared with the Miltenyi Adult Brain Dissociation Kit (Miltenyi Biotec, 130-107-677), followed by enrichment with CD45 micro beads (Miltenyi Biotec, 130-107-677). 10x Genomics Chromium Single Cell 3′ GEM, Library & Gel Bead Kit version 3, was used for single-cell capture and library generation per the user guide. Samples for the AD mouse model were prepared with the same Miltenyi dissociation kit, but enrichment was performed via FACS sorting of live CD11b^+^ cells. Samples were labeled with CellPlex reagents for multiplexing purposes, and single-cell capture was performed using the Chromium Next GEM Single Cell 3′ Kit version 3.1 per the user guide. Detailed information for bulk and scRNA-seq experimental procedures can be found in the Supplementary Methods section.

Raw reads were aligned to the mouse genome (mm10) using STAR aligner^64^. For scRNA-seq, reads were mapped using the standard Cell Ranger pipeline (version 6.1.1/version 7.0.0). Downstream analysis of bulk RNA-seq data was carried out in R. Differential expression was performed using the limma/voom pipelines^65^. GSEA was performed with the fgsea^66^ and sparrow (https://github.com/lianos/sparrow) packages using signatures from the hallmark Molecular Signatures Database (MSigDB)^67,68^. For scRNA-seq, analyses were performed in R using the standard Seurat version 4 pipeline^69^. Pseudobulk differential expression analyses were carried out using limma/voom. Detailed information about processing and analysis steps can be found in the Supplementary Methods; for analysis code, see the ‘Code availability’ section.

### IHC for mouse brains

After cardiac perfusion with ice-cold PBS, right hemibrains were fixed by immersion in 4% paraformaldehyde (PFA) at 4 °C for 24 hours and then transferred to 30% sucrose solution for 2 days before sectioning coronally on a freezing microtome at a thickness of 40 μm. Free-floating sections were permeabilized with 1× Tris-buffered saline solution containing 0.05% Tween (TBST), blocked with 5% donkey serum and incubated in primary antibodies overnight at 4 °C. Sections were then washed in TBST, and a solution of secondary antibodies was then applied for 1 hour at room temperature. Sections were washed in TBST before mounting and cover-slipping with ProLong Glass Antifade Mountant solution (Thermo Fisher Scientific, P36984). Immunofluorescence was performed using the following primary antibodies: goat anti-Iba1 (Novus, NB100-1028, 1:500); guinea pig anti-Iba1 (Synpatic Systems, HS-234 308, 1:500); rabbit anti-CD74 (Abcam, ab245692, 1:500); and goat anti-Axl (R&D Systems, AF854, 1:25) and the following secondary antibodies: Alexa Fluor 488 donkey anti-Rabbit IgG (Jackson ImmunoResearch, 711-545-152, 1:500); Alexa Fluor 555 donkey anti-rabbit IgG (Thermo Fisher Scientific, A32794, 1:500); Alexa Fluor 647 donkey anti-goat IgG (Thermo Fisher Scientific, A-21447, 1:500); and Alexa Fluor 647 donkey anti-guinea pig IgG (Jackson ImmunoResearch, 706-605-148, 1:500). To quantify microglia coverage in the brains, immunohistochemical staining by DAB precipitation coloring was also performed after goat anti-Iba1 primary incubation. The sections with immunofluorescence staining were imaged and analyzed for microglia morphology. See Supplementary Methods for details on microglia morphology quantification.

### Detection of antibody concentrations

Antibody concentrations were quantified using a generic anti-human IgG sandwich-format ELISA. Plates were coated overnight at 4 °C with donkey anti-human IgG (Jackson ImmunoResearch, 709-006-098) at 1 µg ml^−1^ in sodium bicarbonate solution (Sigma-Aldrich, C3041-50CAP) with gentle agitation. Plates were washed 3× with PBS + 0.05% Tween 20. Assay standards and samples were diluted in PBS + 0.05% Tween 20 + 1% BSA (10 mg ml^−1^). A standard curve ranging from 0.41 ng ml^−1^ to 1,500 ng ml^−1^ (0.003–10 nM) was fitted using a four-parameter logistic regression. Standards and diluted samples were incubated with agitation for 2 hours at room temperature. Plates were washed 3×. Detection antibody, HRP-conjugated goat anti-human IgG (Jackson ImmunoResearch, 109-036-098), was diluted in blocking buffer (PBS + 0.05% Tween 20 + 5% BSA (50 mg ml^−1^)) to a final concentration of 0.02 µg ml^−1^, and plates were incubated with agitation for 1 hour at room temperature. After a final 3× wash, plates were developed by adding TMB substrate for 5–10 minutes. Reaction was quenched by adding 4N H_2_SO_4_. Absorbance at 450 nm was measured.

### In vivo detection of mouse microglial proliferation

Animals were dosed with EdU solution (20 mg ml^−1^, Santa Cruz Biotechnology, sc-284628) via intraperitoneal (IP) injection at 80 mg kg^−1^ at day 0 (30 minutes after antibody treatment), then at  1, 2, and 3 days. Four days after EdU injection, animals were deeply anesthetized via IP injection of 2.5% Avertin. The mice were then perfused intracardially with cold PBS. Left hemibrain was snap-frozen on dry ice for biochemical analysis. Right hemibrain was fixed in 4% PFA at 4 °C for 24 hours for sectioning and immunostaining. For detecting EdU^+^ microglia in brain sections, Click-iT EdU imaging kits (Thermo Fisher Scientific, C10637) were used following the manufacturer’s instructions before the Iba1 immunofluorescence staining procedures.

### Generation of TREM2-DAP12 overexpression HEK293 stable cell lines

Human TREM2/DAP12 is overexpressed in HEK293 cell line (RRID: CVCL_0045, ATCC: PTA-4488) by transfection of a dual promoter pBudCE4.1 vector driving expression of TREM2 under the CMV promoter and DAP12 the under the EF1a promoter.

Stable clones were isolated after zeocin selection (800 µg ml^−1^ for 10 days), and TREM2 was detected by flow cytometry (APC-conjugated rat anti-human/mouse-TREM2 monoclonal antibody, R&D Systems, MAB17291). The clone showing the highest TREM2 expression level was selected.

### pSyk signaling

Phospho-Syk was measured by the AlphaLISA assay following the manufacturer’s protocol (PerkinElmer, ALSU-PSYK-A10K). In brief, hTREM2-DAP12 HEK293 cells were plated at 50,000 cells per well the day before the assay. Cells are stimulated with TREM2 antibodies for 5 minutes at 37 °C using Bravo liquid handler. For the TfR blocking experiment, TREM2 antibody was mixed with a custom-made monoclonal TfR antibody that binds the ATV epitope in the apical domain (K_D_ ~0.9 nM) at 37 °C for 30 minutes before adding to the cells. Cells are lysed with lysis buffering containing 1 µM PMSF. Data are collected from PerkinElmer EnVision plate reader.

### IP assay

hTREM2-DAP12 HEK293 cells were plated on 10-cm dishes coated with PDL 24 hours before antibody treatment. Cell lysate was collected with 1-ml ice-cold IP buffer (Thermo Fisher Scientific, 87787) containing 1× protease inhibitor (Cell Signaling Technology, 5872) using a cell scraper. Cell lysate was normalized based on BCA measurement. Then, 200–400 µg of total lysate was used for each IP and was mixed with 2 µg of biotinylated capturing antibody at 4 °C overnight. Normal IgG from the same species as the IP capturing antibody was used as binding control. Antibody biotinylation was performed using an EZ-Link Sulfo-NHS-LC-Biotin Kit, following the manufacturer’s instructions (Thermo Fisher Scientific, A39257). For TREM2 IP, goat anti-TREM2 antibody (R&D Systems, AF1828) and normal goat IgG (R&D Systems, AB-108-C) was used as capturing antibody. For the reciprocal TfR IP, a human monoclonal clonal antibody against TfR and an isotype IgG isotype antibody were made in-house for the study. The immune complex was then precipitated using 20 µl of streptavidin-conjugated magnetic beads (Resyn Biosciences, MR-STV005) and washed 4× with ice-cold 1× PBS buffer containing 0.05% Tween 20. For protein elution, 25 µl of LDS containing 1× sample buffer (Thermo Fisher Scientific, NP0007) and 1× reducing agent (Thermo Fisher Scientific, NP0009) was added directly to the washed beads and incubated at 75 °C for 10 minutes. Samples were then analyzed following standard western blot protocol. The following antibodies were used for western detection: anti-TREM2 (R&D Systems, AF1828), anti-TfR (Thermo Fisher Scientific, 13-6800) and anti-actin (Cell Signaling Technology, 58169S).

### BioID-based antibody clustering assay

TREM2 fusion protein tagged with BioID2, a mini biotin ligase^42^, was cloned into the pLVX-TRE3G vector (Takara, 631187) using In-Fusion reagent (Takara, 638943). To further optimize the catalytic activity of BioID2, four additional point mutations (E62R, q68g, p70g and S112A) were added to the sequence as previously described^70^. The pLVX-TRE3G-TREM2-BioID2 plasmid and the transponder pLVX-EF1a-Tet3G plasmid (Takara, 631359) were introduced into the parental hTREM2-DAP12 HEK293 cells as stable cell line. Cells were maintained in biotin-free media containing DMEM (Gibco, 11965092), 10% dialyzed FBS (R&D Systems, S12850), 1× NEAA (Gibco, 11140-050), 1× sodium pyruvate (Gibco, 11360-070) and 1× P/S (Gibco, 15140-122). Twenty-four hours before the assay, 2–4 ng ml^−1^ of Dox was added to initiate TREM2-BioID fusion protein expression. Cells were treated with antibody solution containing 100 nM TREM2 antibody and 2 µM biotin prepared in 1× PBS for 10 minutes at 37 °C. Both no-Dox and no-biotin samples were included as negative control. Cell lysates were prepared with RIPA buffer (Teknova, R3792) containing 1× phosphatase/protease inhibitor (Cell Signaling Technology, 5872). Normalized protein lysate containing 0.5–1 mg of protein was incubated with 20 µl of magnetic streptavidin beads (Resyn Biosciences, MR-STV005) at 4 °C overnight. Beads were then washed 4× with ice-cold RIPA buffer. Captured protein was eluted with 2× NuPAGE LDS sample buffer (Thermo Fisher Scientific, NP0007) supplied with additional 1% SDS, 20 mM biotin and 1× reducing agent (Thermo Fisher Scientific, NP0009) and heated at 95 °C for 10 minutes. Samples were analyzed via western blot. Goat anti-TREM2 (R&D Systems, AF1828) and anti-goat HRP secondary antibody (Thermo Fisher Scientific, 81-1620) were used for detection with ECL reagent (Bio-Rad, 1705060). For quantification, TREM2 biotinylation near 28 K_D_ was normalized to the auto-biotinylation of TREM2-BioID band above 55 K_D_.

### TREM2 receptor and ATV:TREM2 trafficking endosomal signaling by immunofluorescence microscopy

Human hTREM2-DAP12 HEK293 cells were plated at 35,000 cells per well 1 day before the experiment. Cells were starved in serum-free DMEM for 1 hour before antibody stimulation at 10 nM and 37 °C for 10 minutes. Transferrin is labeled with Alexa Fluor 647 transferrin conjugate (Invitrogen, T23366) at 20 µg ml^−1^ during antibody stimulation. Cells were fixed in 4% PFA for 10 minutes and permeabilized/blocked in PBS containing 0.3% Triton and 5% BSA. Primary antibodies were diluted (1:250) in PBST buffer (0.06% Triton and 1% BSA in PBS) and used at 4 °C for 24 hours with anti-pSyk Tyr525/526 (Cell Signaling, 2711) and anti-EEA1 (BD Biosciences, 610456). Secondary antibodies were diluted (1:500) in PBST buffer and used at room temperature for 45 minutes with Alexa Fluor 488 anti-human IgG (Jackson ImmunoResearch, 109-545-003); Alexa Fluor 568 anti-rabbit (Thermo Fisher Scientific, A10042); and Alexa Fluor 647 anti-mouse (Thermo Fisher Scientific, A31572). Cells were washed with PBS 3× between each stain.

Data were acquired with Opera Phoenix High-Content Imager (PerkinElmer) at ×63 magnification. Image quantification was performed using Harmony software (PerkinElmer, version 5.1) with spot identification algorithm to quantify the fluorescence spot intensity by channel. A masking algorithm was implemented in Harmony software to quantify the percent of IgG or pSyk spots co-localized within EEA1 or TfR.

### Western blot detection of signaling pathway activation in iMG

Cell lysates were prepared in high-salt lysis buffer (Cell Signaling Technology, 9803) with protease and phosphatase inhibitors (Cell Signaling Technology, 5872). BCA assay (Thermo Fisher Scientific, 23225) was used to measure total protein. Normalized lysates were mixed with loading buffer (Thermo Fisher Scientific, NP0007) and reducing agent (Thermo Fisher Scientific, NP0004) and then resolved by electrophoresis using 4–12% NuPAGE (Thermo Fisher Scientific, NP0335BOX). Semi-dry transfer was performed using the Trans-Blot Turbo System (Bio-Rad, 1704150) with 0.2-µm PVDF transfer packs (Bio-Rad, 17001917). Transferred blots were blocked with Intercept Blocking Buffer (LI-COR, 927-60001) and incubated with primary antibody overnight. HRP-conjugated secondary antibodies were used for detection. Washes were performed using TBST buffer containing 0.05% Tween 20. Blots were developed using ECL reagent (Bio-Rad, 1705062) and imaged by the Li-COR Odyssey Fc imaging system. Band signal intensity was quantified using Image Studio Lite software (LI-COR, version 5.2.5).

Table of Western blot antibody reagents.

**Table:** 

Antibody	Source	Cat.	Dilution
mTOR (7C10)	CST	2983T	1:500
Phospho-mTOR (Ser2448)	CST	5536T	1:500
AKT (C67E7)	CST	4691T	1:500
Phospho-Akt (Ser473)	CST	9271T	1:250
Phospho-RPS6 (Ser235/236)	CST	4858T	1:500
Phospho-GSK-3β (Ser9)	CST	5558T	1:500
β-actin (E4D9Z)	CST	58169S	1:1,000
Phospho-4E-BP1 (Thr37/46)	CST	2855T	1:250
Phospho-Erk1/2 (Thr202/Tyr204)	CST	4370S	1:1,000
Anti-rabbit HRP 2nd antibody	CST	7074S	1:2,000
Anti-mouse HRP 2nd antibody	CST	7076P2	1:2,000

### iMG proliferation assay

iMG were plated in cell carrier ultra 96-well plate (PerkinElmer, 6055302) at a density of 25,000 cells per well and equilibrated for 72 hours in IMDM media (Thermo Fisher Scientific, 12440061) containing 10% FBS (Hyclone, SH30071.03), 20 ng ml^−1^ of granulocyte-macrophage colony-stimulating factor (GM-CSF), 20 ng ml^−1^ of IL-3 and 20 ng ml^−1^ of M-CSF. Cells were dosed with 100 nM antibody prepared in reduced cytokine media containing 10% FBS (Cytiva, SH30071.03), 5 ng ml^−1^ of GM-CSF, 5 ng ml^−1^ of IL-34 and 5 ng ml^−1^ of CSF1. Twenty nM mTOR kinase inhibitor AZD8055 (AZD, Selleck Chemicals, S1555) was dosed with antibody. Forty-eight hours after treatment, 20 µM EdU was added, and cells were treated for 96 hours before being fixed by 4% PFA at room temperature for 10 minutes and stained with EdU labeling kit (Thermo Fisher Scientific, C10637), DAPI (Thermo Fisher Scientific, 62248) and cell mask (Thermo Fisher Scientific, H32721). Fluorescent images were collected on Opera Phenix High-Content Imaging system equipped with a ×20 water lens (NA = 1.0). A total of nine fields per well were collected using three-step z-planes separated by 2 μm. Total cell count was measured by counting DAPI^+^ nuclei using the ‘find nuclei’ function of Harmony software (PerkinElmer, version 4.9). EdU^+^ nuclei were quantified from the 488 channel, and frequency was calculated by normalizing to total DAPI^+^ nuclei.

### CSF1R detection assay

CSF1R levels in brain homogenates and CSF were quantified using a commercial ELISA assay (Abcam, ab240681). Brain homogenates were diluted 40-fold, and CSF was diluted 50-fold in NS diluent buffer supplied from the kit. Samples were assessed in duplicate wells, and the average reading was taken as the CSF1R protein level for that sample.

### Ex vivo phagocytosis assay

Mice were taken down 2 days after antibody dosing, and brains were dissected after PBS perfusion and dissociated with the Adult Brain Dissociation Kit (Miltenyi Biotec, 130-107-677), according to the manufacturer’s protocol. Microglia number was measured by FACS using CountBright Absolute Counting Beads (Invitrogen, C36950) and diluted to 500 microglia per µl in DPBS + 0.5% BSA. The resulting cell suspension was mixed with pHrodo-green labeled myelin (50 µg ml^−1^ in DPBS + 0.5% BSA) or FAM-Aβ (200 nM in DPBS + 0.5% BSA) and incubated at 37 °C for 45 minutes with gentle mixing. Cell suspensions were washed and stained with the following antibodies in FACS buffer (1% fatty acid-free BSA and 1 mM EDTA in PBS) for 25 minutes on ice: CD11b-BV421 (BioLegend, 101251) and mouse Fc blocker (anti-mouse CD16/32, BioLegend, 101320). Cells were washed with FACS buffer, resuspended in FACS buffer with propidium iodide (Miltenyi Biotec, 130-93-233), strained through a 100-μm filter and then analyzed on a flow cytometer (BD FACSAria III). FCS files were then imported and analyzed in FlowJo software (version 10).The percentage of myelin^+^ microglia (pHrodo-green^+^, CD11b^+^) and Aβ^+^ microglia (FAM^+^, CD11b^+^) in the total CD11b^+^ microglial population was calculated. See Supplementary Methods for details on myelin debris and Aβ fibril preparation and fluorescent labeling.

### In vitro iMG lipid storage assay

iMG (30,000 cells per well) were plated on PDL-coated 96-well plates in microglia differentiation media. After 24 hours at 37 °C, media containing 15 µg ml^−1^ of purified unlabeled myelin^71^ was added (see Supplementary Methods for details). After 24 hours at 37 °C, media was exchanged for media containing 100 nM ATV:TREM2 or ATV:ISO antibody and incubated for another 48 hours at 37 °C before imaging BODIPY staining or lipidomics extractions. For BODIPY imaging, cells were incubated at 37 °C for 30 minutes in live cell imaging buffer (Life Technologies, A14291DJ) containing 1 µM BODIPY 493/503 (Thermo Fisher Scientific, D3922) and 1 drop per milliliter of NucBlue (Thermo Fisher Scientific, R37605). Cells were fixed in 4% PFA and imaged using Alexa Fluor 488 and DAPI on an Opera Phenix High-Content confocal imager. Lipid spots were analyzed using a spot-finding algorithm on Harmony software.

For lipidomic analysis, cells were washed once with PBS on ice. Then, 100 µl of a 9:1 methanol:water solution containing 2 µl of internal standard mixture was added to the cells. The plate was agitated on a shaker at 4 °C and 1,200 r.p.m. for 20 minutes and centrifuged for 5 minutes at 300*g*. A sample of supernatant was transferred to LC–MS vials and kept at −80 °C. See Supplementary Methods for details LC–MS analysis.

### Seahorse detection of cellular respiration

iMG (20,000 cells per well) were seeded on a PDL-coated 96-well Agilent Seahorse XF Cell Culture microplate in media. For the fatty acid oxidation studies, 3 days before assay, media replaced with substrate-limited media comprised of XF DMEM, 1% FBS, 0.5 mM glucose, 1 mM glutamine and 0.5 mM carnitine. Antibodies were added to cells for a final concentration of 100 nM and incubated for 3 days. Cells were washed twice, and antibody was re-added to the washed cells to a final concentration of 100 nM. Cells were imaged using bright-field microscopy to obtain cell counts for normalization. Cells were then incubated for 1 hour in a non-CO_2_ incubator. Ports on the sensor plate were filled according to the XF Long Chain Fatty Acid Oxidation Stress Kit or XF Glucose Oxidation Kit, and cells were subjected to sequential injections of oligomycin (final concentration 1.5 µM), FCCP (2-[2-[4-(trifluoromethoxy)phenyl]hydrazinylidene]-propanedinitrile) (2 µM for fatty acid oxidation and 1.5 µM for glucose oxidation) and rotenone/antimycin A (0.5 µM each). In experiments using inhibitors, etomoxir or UK5099 were added in port A at 4 µM or 3 µM, respectively. Data were analyzed using the Agilent Seahorse Analytics online software to generate kinetic curves and calculate maximal respiration and spare capacity.

### PET imaging study

On day 0, 5–6-month-old WT;hTREM2 tg;TfR^mu/hu^ mice and 4.5-months-old 5×FAD;hTREM2 tg;TfR^mu/hu^ mice (*n* = 6 each) were IP injected with ATV:TREM2 or an isotype control antibody at 14 mg kg^−1^ and 10 mg kg^−1^, respectively. Mice were subjected to either TSPO-PET or FDG-PET imaging 24 hours after dose. Microglia activation and brain glucose metabolism were followed longitudinally with further PET scans on days 4 and 8 after antibody administration. Male and female mice were distributed evenly among both antibody treatment and PET imaging groups. See Supplementary Methods for details on PET imaging procedures and quantification.

### Statistics and reproducibility

All statistical analyses were run in GraphPad Prism 9 or R Studio (version 1.4.1717). Data are presented as mean ± s.e.m. with raw dot plots. The number of animals, cells, imaging fields, experiment replicates and statistical tests are indicated in the figure legends. All in vitro data are from at least three independent experiments. The samples were not blinded during initial study planning to ensure that the number of groups of mice were randomized and balanced, and age and sex were matched. RNA-seq datasets were not blinded for analysis. Image analysis and ex vivo assays were performed in a blinded fashion. In vitro studies were performed unblinded. The PET imaging analysis was not blinded; however, we used an automated pipeline^72,73^ such that the operator cannot influence/bias the results because the co-registration and the voi extraction is a defined procedure.

All statistical analyses performed were two-tailed. Data normality was examined by the Shapiro–Wilk test. *F*-test or Brown–Forsythe test was used to assess data homoscedasticity. If data meet statistical assumptions, unpaired *t*-test, paired *t*-test, Dunnett’s multiple comparisons test, Tukey’s multiple comparisons test or multiple *t*-test was used depending on data groups. For non-parametric statistics, unpaired *t-*test with Welch’s correction test or Kruskal–Wallis test was used. Data with normal distribution but unequal variance were assessed with Mann–Whitney test or Brown–Forsythe and Welch’s ANOVA test. Statistical *P* values are shown on graphs denoted with ‘*P* = ’.

### Reporting summary

Further information on research design is available in the Nature Portfolio Reporting Summary linked to this article.

## Online content

Any methods, additional references, Nature Portfolio reporting summaries, source data, extended data, supplementary information, acknowledgements, peer review information; details of author contributions and competing interests; and statements of data and code availability are available at 10.1038/s41593-022-01240-0.

## Supplementary information

Supplementary Information Supplementary Tables 1–3; Extended Data Figs. 1–8; Supplementary Figs. 1–4; Supplementary Materials and Methods; and References

Reporting Summary

Supplementary Table 1 Supplementary Tables 4–6

## Data Availability

The raw and processed bulk/scRNA-seq data have been deposited in the National Center for Biotechnology Information’s Gene Expression Omnibus (GEO) and are accessible through GEO SuperSeries accession numberGSE200276. This SuperSeries is composed of three individual series: GSE199154, GSE198987 and GSE200275. Raw and processed files for the AD model scRNA-seq study are accessible through GEO accession number GSE209912. The metabolomics data were uploaded to the MetaboLights repository with study ID MTBLS6543. Source data are provided with this paper.

## Source data

Source Data Fig. 1 Unprocessed western blots
Source Data Fig. 2 Unprocessed western blots
Source Data Fig. 3 Unprocessed western blots

## Code availability

All R and Python code used to produce transcriptomics analyses and imaging morphometric analyses are available through GitHub at the following URL: https://github.com/denalitherapeutics/Lengerich_natneuro_2022.
