# Supplementary material for: A TREM2-activating antibody with a blood–brain barrier transport vehicle enhances microglial metabolism in Alzheimer’s disease models
Source: Nat Neurosci. 2023 Jan 12;26(3):416–29. doi: 10.1038/s41593-022-01240-0 (PMC9991924; doi:10.1038/s41593-022-01240-0)
Supplement: Supplementary file 1 — Supplementary Tables 1–3; Extended Data Figs. 1–8; Supplementary Figs. 1–4; Supplementary Materials and Methods; and References [file 41593_2022_1240_MOESM1_ESM.pdf]

nature neuroscience

Article

https://doi.org/10.1038/s41593-022-01240-0

A TREM2-activating antibody with a blood–brain barrier transport vehicle enhances microglial metabolism in Alzheimer's disease models

In the format provided by the authors and unedited

SUPPLEMENTARY INFORMATION

SUPPLEMENTARY TABLES

Supplementary Table 1. ATV:TREM2 demonstrates reduced or undetectable FcR binding. Octet affinity measurements show ATV:TREM2 and ATV:ISO, which contain LALA mutations in the Fc domain, display reduced or no binding to Fc gamma receptors compared to effector positive hIgG1 positive control antibody.

Fc gamma receptor	ATV:TREM2	ATV:ISO	hIgG1 control
Fc gamma RI	380 nM	400 nM	21 nM
Fc gamma RIIA	No binding	No binding	1.3 uM
Fc gamma RIIB	No binding	No binding	2.9 uM
Fc gamma RIIIA	> 5 uM	> 9 uM	1.3 uM

Supplementary Table 2. Animal information for *in vivo* studies.

Institution where study was conducted	Mouse genotype/age/sex	Corresponding figure
Denali	WT; $TfR^{mu/hu}$ /2 months/male only	Fig. 1A
Denali	WT; $TfR^{mu/hu}$ /3 months/male only	Fig. 1B, left<br>Fig. 1C-H<br>Extended Data 1A-D<br>Extended Data 2
Denali	WT; $TfR^{mu/hu}$ /8 months/only 1 female in ATV:ISO group, the other are all male<br>*App*^{SAA}; $TfR^{mu/hu}$ mice /8 months/male only	Fig. 1B, right<br>Fig. 1C-H<br>Extended Data 1A-E
Denali	WT; $TfR^{mu/hu}$ /3 months/male only	Suppl. Fig 1L
Denali	WT; $TfR^{mu/hu}$ /4 months/male only	Suppl. Fig 1I-K

Denali	hTREM2 tg/3 months/mixed sex	Suppl. Fig 3B
Denali	WT; hTREM2 tg; $TfR^{mu/hu}$/4.5 months/mixed sex (male mice only in Day 1 groups, female only in Day 4 groups)	Fig. 4A-E<br>Extended Data 6A
Denali	WT; hTREM2 tg; $TfR^{mu/hu}$/3.5 months/mixed sex in all groups	Fig. 4F,<br>Extended Data 6B, C,G-I
Denali	WT; hTREM2 tg; $TfR^{mu/hu}$/3.5 months/mixed sex in all groups	Fig. 4G
Denali	WT; hTREM2 tg; $TfR^{mu/hu}$/3 months/mixed sex in all groups	Fig. 5L-M,<br>Extended Data 8G
Denali	WT; hTREM2 tg; $TfR^{mu/hu}$/3 months/mixed sex in all groups	Extended Data 6J
Denali	WT; hTREM2 tg; $TfR^{mu/hu}$/3 months/mixed sex (male mice only in Day 1 groups, female only in Day 4 groups)	Extended Data 6D-F
Invicro	WT; hTREM2 tg; $TfR^{mu/hu}$/4-5 months/mixed sex in all groups	Extended Data 7
DZNE	WT; hTREM2 tg; $TfR^{mu/hu}$/5-6 months/mixed sex<br>5xFAD; hTREM2 tg; $TfR^{mu/hu}$/4.5 months/mixed sex	Fig. 6A-H
Denali	WT; $TfR^{mu/hu}$ /3 months/male only	Suppl. Fig 1A-H and Suppl. Fig 2
Denali	WT; hTREM2 tg; $TfR^{mu/hu}$/5-6 months/mixed sex<br>5xFAD; hTREM2 tg; $TfR^{mu/hu}$/4.5 months/mixed sex	Suppl. Fig 4

Supplementary Table 3. Statistical analysis of sex effect of ATV:TREM2 on microglia function and biomarker response. A 2-way main effects ANOVA of sex and treatment group was used to test for sex differences in response to ATV:TREM2 for microglial phagocytic activity and CSF1R levels in brain and CSF. No significant sex differences were identified.

Endpoint	Corresponding figure panels	Animal N and sex	Treatment:<br>ATV:TREM2 3, 10mg/kg, or ATV:ISO 10mg/kg
%pHrodo$^+$ phagocytic microglia	Fig. 4F	3 Male / 5 Female in each ATV:TREM2 group	ns, p-value = 0.4922
Brain CSF1R	Extended Data Fig 6B	3-5 Male / 4-5 Female in each ATV:TREM2 group	ns, p-value = 0.4391
CSF CSF1R	Extended Data Fig 6C	5 Male / 4-5 Female in each ATV:TREM2 group	ns, p-value = 0.0996

EXTENDED DATA FIGURES

Extended Data Figure 1. ATV:4D9 induces temporally dynamic microglial states distinct from amyloid pathology. (A) UMAP projections of individually processed data sets for WT; TfR$^{mu/hu}$ timecourse and TfR$^{mu/hu}$; *App*^{SAA} studies. Microglia are color coded according to their experimental group. **(B)** Combined UMAP of integrated data by study. Microglia are color coded by unbiased cluster assignment. **(C)** Stacked barplots showing the proportion of microglia per biological replicate by cluster. Plots are grouped by experimental group, and each bar represents a biological replicate within that group. Barplot color scheme is consistent with clusters in **B**. **(D)** Feature plots showing expression of selected individual genes. Microglia are color coded according to log normalized expression of each gene. **(E)** Antibody concentrations detected in whole brain lysate from either WT; TfR$^{mu/hu}$ or TfR$^{mu/hu}$; *App*^{SAA} mice dosed with 10 mg/kg ATV:ISO or ATV:4D9 (n=8 mice/group, except for ATV:4D9 WT;TfR$^{mu/hu}$ and 4D9 APP^{SAA}; TfR$^{mu/hu}$ n=4 mice/group)

Extended Data Figure 2. ATV:4D9 induces temporally dynamic microglial morphology and marker expression. (A) Representative morphometric images of microglia in the cortex 1 day post dose with ATV:ISO (10 mg/kg) or ATV:4D9 (10 mg/kg) at day 1-, 7-, 14-, and 28-days post dose. **(B)** UMAP plot of all microglia over time. **(C)** Percentage of microglia in the responsive cluster as a proportion of all segmented microglia over time (n=3 male mice per group, two-tailed unpaired t-test between ATV:ISO and ATV:4D9 at day 1, mean±SEM). **(D)** Volcano plot showing the top 6 differentially high and low normalized features comparing the responsive cluster to the

homeostatic cluster. **(E)** Heatmap of normalized features for all segmented microglia (1,143 total cells) over time, measured across 65 morphometric and immunohistochemical features, grouped by treatment (rows) with features hierarchically clustered (columns). **(F)** Representative images of cortical brain sections co-stained with Iba1 and CD74 at 1-, 7-, 14-, 28-days days post 10mg/kg dose of ATV:ISO or ATV:4D9. CD74$^+$ microglia are noted with white arrows. (**G**) Mean intensity of CD74 staining within segmented Iba1$^+$ microglia normalized to background CD74 intensity at each timepoint (n=5 mice/group, two-tailed unpaired t-test between ATV:ISO and ATV:4D9 at day 1, mean±SEM). **(H)** Representative images of cortical brain sections stained for Iba1 and AXL 1 day post dose of ATV:ISO or ATV:4D9. Double positive microglia are noted with white arrows. **(I)** Quantification of mean intensity of AXL staining within segmented Iba1$^+$ microglia normalized to background (n=5 mice/group, two-tailed unpaired t-test, mean±SEM).

Extended Data Figure 3. ATV:4D9 and ATV:TREM2 demonstrate similar mechanisms of action with high affinity stalk binding epitopes and cellular function. (A) Antibody schematic comparing human specific ATV:TREM2 and mouse specific ATV:4D9 with high affinity TREM2 binding. **(B)** Epitope map of overlapping stalk binding regions for ATV:TREM2 and ATV:4D9 Fabs (space filled model of TREM2 ECD[1]). The binding epitope of ATV:4D9 antibody is located 12-amino acids N-terminal of the ADAM cleavage site at His157. **(C)** FACS analysis of cell binding of ATV:TREM2 to hTREM2-DAP12 HEK293 or parental cells. Endogenous TfR expression on HEK293 cells drives weak binding observed for ATV:ISO and ATV:TREM2 (n=3 independent

experiment, mean±SEM). (**D**) FACS detection of ATV:TREM2 (100 nM) binding to WT and TREM2 KO iMG with isotype control (ATV:ISO). **(E)** Soluble TREM2 measured in the supernatant of hTREM2-DAP12 HEK293 cells dosed with ATV:TREM2 for 24h shows dose-dependent reduction of sTREM2 to levels similar to 1uM GM6001 (n=3 independent experiment, mean±SEM). (**F**) ATV:TREM2 and lipid ligands induce pSyk signaling in iMG 24h post antibody exposure (n=3 independent experiments, Tukey's multiple comparisons test, mean±SEM). **(G)** Human monocytes cultured in limited M-CSF with plate coated ATV:TREM2 or ATV:ISO shows dose-dependent activity of ATV:TREM2 (EC50 0.95 +/- 0.45 nM). Representative data from one out of four human donors are shown.

Extended Data Figure 4. **ATV promotes TfR-TREM2 receptor complex formation and internalization and endosomal TREM2 signaling.** (**A**) Representative Western blot of co-IP of TREM2 with TfR. hTREM2-DAP12 HEK293 cells were treated with 100 nM ATV:TREM2, anti-TREM2, or isotype controls for 5 min at 37°C. (**B**) Co-IP quantification of Western blot from (A) (n=6 independent experiments; two-tailed paired t-test for ISO vs anti-TREM2; two tailed Wilcoxon test ATV:ISO vs ATV:TREM2, mean±SEM). (**C**) Schematic illustration of *cis*- and *trans*-activation models that could mediate pSyk enhancement by ATV:TREM2. (**D**) Western blot validation of TfR knockdown in the TfR^{RNAi} cell line. (**E**) Cell based cis/trans assay indicates ATV:TREM2 enhances pSyk activity in cis. Relative pSYK signal is expressed as raw pSYK AlphaLisa value normalized to ATV:TREM2 treated control (n=3 independent experiment, mean±SEM). **(F)** Normalized pSyk signal measured by AlphaLisa assay. TfR^{RNAi} cells

were treated with 10 nM anti-TREM2 or ATV:TREM2 pre-incubated with a dose titration of recombinant TfR protein for 5 min at 37°C (n=3 independent experiment, mean±SEM). (**G**) Normalized pSyk signal detected by AlphaLisa. TfR^{RNAi} cells were treated with 10 nM anti-TREM2 or ATV:TREM2 pre-incubated with a dose titration of a secondary anti-human IgG Fc antibody for 5 min at 37°C (n=3 independent experiment, mean±SEM) **(H)** Immunofluorescence microscopy of hTREM2-DAP12 HEK293 cells demonstrates reduction of surface TREM2 levels with ATV:TREM2 vs anti-TREM2, no changes in total TREM2 levels, consistent with re-distribution of the receptor from the plasma membrane to endosomes (n=4 independent experiments except for anti-TREM2 MV (n=3), Tukey's multiple comparisons test, mean±SEM). **(I)** hTREM2-DAP12 HEK293 cells dosed with antibody for 10 minutes shows that at similar amounts of bound antibody detected by anti-IgG (representing 5 nM of ATV:TREM2 and 10 nM of anti-TREM2, n=4 independent experiments, Tukey's multiple comparisons test, mean±SEM**(J)** Images depicting masking algorithm used to identify whether TfR-Alexa-647 labeled recycling endosomes (rainbow spots in middle images) either contain (green spots in right-most images) or do not contain (red spots in right-most images) IgG spots (white spots in left-most image) upon dosing with anti-TREM2 (top row) or ATV:TREM2 (bottom row) for 10 minutes with 10 nM antibody. **(K)** Representative images for hTREM2-DAP HEK293 cells dosed with 10 nM antibody for 10 minutes including 20 ug/mL TfR-Alexa-647, fixed, permeabilized, and stained with anti-IgG and anti-pSyk. IF shows ATV increased colocalization of antibody with pSyk in early endosomes. **(L)** Quantification of percent of IgG or pSyk spots localized within Tf-positive endosomes (n=3 independent experiments, Tukey's multiple comparisons test, mean±SEM).

Extended Data Figure 5. ATV:TREM2 does not promote ERK1/2 phosphorylation or a proinflammatory signature in microglia. (**A**) Representative Western blot images of phosphorylation of 4EBP1 (T37/46) and ERK1/2 (T202/Y204) in WT iMGs treated for 96h with 100 nM ATV:TREM2 or an isotype control. (**B**) Quantification of p4EBP1 (T37/46) and pERK1/2 (T202/Y204) normalized to actin. Relative expression was calculated by normalizing to PBS vehicle control for each experiment (n=4 independent experiments, two-tailed paired t-test, mean±SEM). (**C**) Representative Western blot images of total protein levels of mTOR and AKT in WT iMG treated for 96 h with 100 nM ATV:TREM2 or an isotype control. (**D**) Quantification of total mTOR and AKT protein normalized to actin. Relative expression was calculated by normalizing to PBS control for each experiment (n=4 independent experiments, two-tailed paired t-test, mean±SEM). **E**) Representative Western blot images for p-mTOR (S2448), pAKT (S473), pGSK3b (S9) pRPS6 (S235/236), p4EBP1 (T37/46) and pERK1/2 (T202/Y204) showing inhibition of mTOR pathway activation in WT iMG co-treated with 20 nM AZD8055 and 100 nM ATV:TREM2 after 96h. (**F**) Quantification of phosphorylation targets shown in (E). Phosphorylation signals were normalized to actin. Relative expression was calculated by normalizing to PBS vehicle control for each experiment (n=4 independent experiments, two-tailed paired t-test, mean±SEM)). (**G**) Heatmap of human cytokine profiling in supernatant from WT iMG treated with 100 nM ATV:TREM2 for 96h. Media collected from iMG treated with 10 ng/mL LPS for 24h was used for comparison.

Extended Data Figure 6. ATV:TREM2 increases phagocytosis and CSF1R in TfR$^{mu/hu}$ human TREM2 transgenic mice (A) Antibody levels detected in brain lysates

by ELISA shows increased brain exposure of ATV:TREM2 (30 mg/kg) compared anti-TREM2 (30 mg/kg) and comparable brain exposure for ATV:TREM2 at 10 mg/kg and anti-TREM2 at 30 mg/kg at day 4 post-dose. (n=5 mice/group except for ATV:TREM2 3mpk (n=4)). **(B)** ATV:TREM2 increases CSF1R in the brain compared to brain exposure matched anti-TREM2 day 2 post dose (n=[8, 9, 8, 9, 10]mice/group, Kruskal-Wallis test, mean±SEM). (**C**) CSF1R analysis in CSF same as in B. (n=[8, 9, 8, 9, 10]mice/group, Tukey's multiple comparisons test, mean±SEM) (B-C) Circles represents male mice and triangle represents female mice. **(D)** Detection of antibody concentrations show matched brain exposure of three ATV:TREM2 molecules with different ATV affinities 1-day post-dose (n=5 mice/group). **(E-F)** High and mid-affinity ATV:TREM2 molecules induce comparable increase of CSF1R in the brain (E, n=5 mice/group except for Veh and Day4-10mpk-8000nM (n=4), Kruskal-Wallis test, compared to vehicle group**)** and CSF **(F)** (n=5 mice/group, except for Veh and day 4 5mpk 110nM n=4), Dunnett's multiple comparisons test for day 1, Kruskal-Wallis test for day 4) day 1 and 4 post dose whereas low-affinity ATV:TREM2 induces a weak elevation of CSF1R in CSF at day 4 (n=4-5 mice/group, mean±SEM). **(G)** Schematic of experimental approach to evaluate *in vivo* dosed antibody impact to microglial phagocytosis *ex vivo*. A single dose of ATV:TREM2, anti-TREM2, or ATV:ISO was administrated to human TREM2 tg; TfR$^{mu/hu}$ KI mice and brain microglia were isolated 2 days post dose for *ex vivo* myelin phagocytosis analysis. The same method was used to assess amyloid phagocytosis. **(H)** FACS gating strategy to quantify pHrodo-myelin positive microglia. After treatment with pHrodo-green myelin and staining, cell suspensions containing microglia were analyzed using BD FACS Aria **III**. Single cells were separated from debris by FSC and SSC characteristics. Live microglia were

identified as a population of CD11b+ and Propidium Iodide^negative cells. pHrodo-myelin uptake was then quantified in 20,000 microglia recorded from each sample. **(I)** Antibody concentrations were detected in brain lysates by ELISA which shows comparable levels of ATV:TREM2 and anti-TREM2 day 2 post dose (n=[9, 10, 10, 9, 10] mice/group). **(J)** ATV:TREM2 induces transcription of genes associated with phagocytosis*. Axl, Itgax, Lgals3* mRNA levels were detected in isolated brain microglia after peripheral administration of 10mg/kg ATV:TREM2 1-, 4-, and 7-days post dose. Graphs represent bulk mRNA measured by RNA-seq. Data shown as $\log_2$ counts per million (with a pseudocount of 1 added) in each biological replicate (n=8 mice/group, each group compared to ATV:ISO group, Kruskal-Wallis test for Axl, Dunnett's multiple comparisons for Itgax and Lgals3, mean±SEM) .

Extended Data Figure 7: Single photon emission computed tomography imaging demonstrates increased ATV:TREM2 biodistribution and catabolism in brain

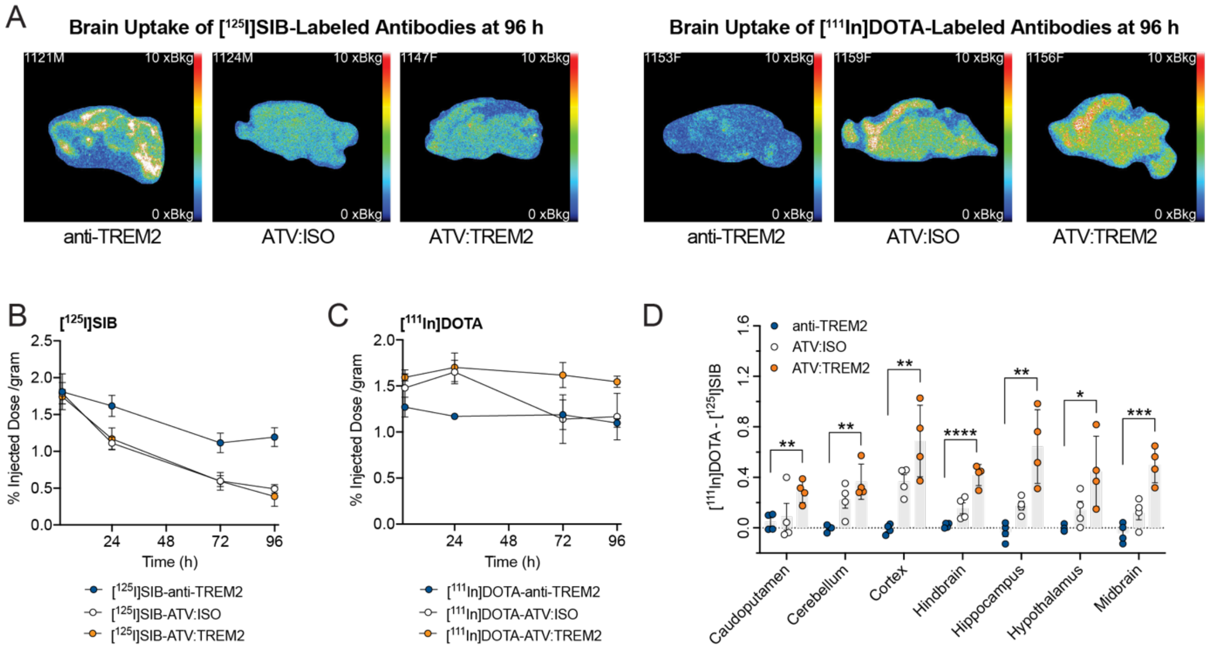

Extended Data Figure 7. Single photon emission computed tomography imaging demonstrates increased ATV:TREM2 biodistribution and catabolism in brain. (A)

Representative autoradiography images of sagittal brain sections from WT; hTREM2 tg; TfR$^{mu/hu}$ mice 96h after administration of ATV:TREM2, anti-TREM2, and ATV:ISO radiolabeled with [^{111}In]DOTA or [^{125}I]SIB. **(B,C)** Longitudinal SPECT/CT imaging quantification of whole-brain uptake of single dose (100 μCi, 200μL, 1.5 mg/kg, IV) ATV:TREM2, anti-TREM2, and ATV:ISO, radiolabeled with [^{125}I]SIB **(B)** or [^{111}In]DOTA **(C)** in WT; hTREM2 tg; TfR$^{mu/hu}$ mice. Whole brain %ID/g was corrected for contribution from cerebral blood volume. Data are represented as mean +/- SEM (n=3 mice/group). **(D)** Percent of catabolized ATV:TREM2 in several brain regions 96h after single dose exceeds that of ATV:ISO control. [^{111}In]DOTA and [^{125}I]SIB signal was quantified by *ex vivo* gamma counting in resected brain regions, and percent of catabolized antibody was estimated by subtracting %ID/g for [^{125}I]SIB signal from that of [^{111}In]DOTA signal (n=4 mice/group, one-way ANOVA with Dunnett's multiple comparison test for each region, except for Cerebellum which applied Brown-Forsythe and Welch ANOVA tests, mean±SEM).

Extended Data Figure 8. ATV:TREM2 increases microglial metabolism in a TREM2 and PLCG2-dependent manner. (A) Additional species of triglycerides (TG) and short chain carnitines modulated in iMG with ATV:TREM2 treatment (n=3-5 independent experiment, two-tailed paired t-test, mean±SEM). **(B)** ATV:TREM2 does not modulate TG in *PLCG2* KO iMG (n=3 independent experiment, two-tailed paired t-test, mean±SEM). **(C)** Maximal respiration measured by Seahorse fatty acid oxidation kit is reduced in both *TREM2* KO and *PLCG2* KO iMG (n=3-4 independent experiment, two-tailed paired t-test, mean±SEM). **(D)** ATV:TREM2 increased spare capacity measured by Seahorse fatty acid

oxidation kit (n=8 independent experiment, two-tailed paired t-test, mean±SEM). **(E)** ATV:TREM2 does not modulate maximal respiration or spare capacity in *TREM2* KO iMG (n=4 independent experiment, two-tailed paired t-test, mean±SEM). **(F)** ATV:TREM2 does not modulate maximal respiration and spare capacity *PLCG2* KO iMG (n=4 independent experiment, two-tailed paired t-test, mean±SEM). **(G)** RNAseq analysis of brain microglia isolated from hTREM2 tg; $TfR^{mu/hu}$ mice dosed with 10 mg/kg ATV:TREM2. GSEA for top pathways based on a p-value cutoff of 0.05 for up- or downregulated gene sets 1 day post ATV:TREM2 dose.

SUPPLEMENTARY FIGURES

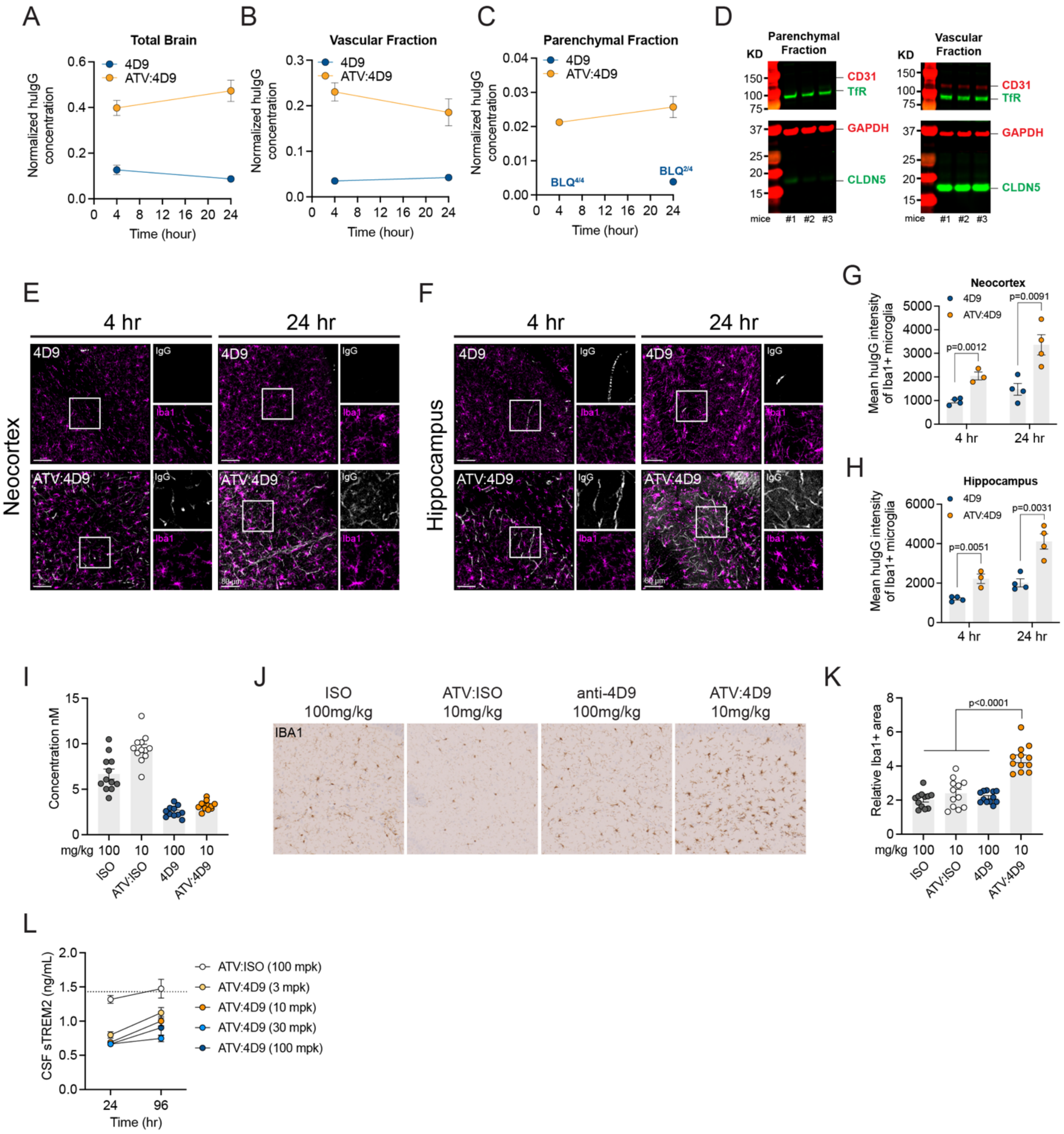

Supplementary Figure 1. ATV improves delivery of a TREM2 antibody to the brain parenchyma, microglial co-localization and Iba1 response. **(A-C)** 50 mg/kg of 4D9 and ATV:4D9 were administered IV TfR$^{mu/hu}$ mice and antibody concentrations were measured 4 and 24h post-dose by huIgG ELISA assay in **(A)** total brain lysate, **(B)** brain

vasculature fraction, and **(C)** brain parenchymal cell fraction. Data were normalized to total protein used for each measurement. Samples with IgG levels below the limit of quantification (BLQ) are noted on the graph. All groups included n=4 mice, except for the 4h 4D9 group in the vascular fraction (B, n=3). **(D)** Western blot demonstrating capillary depletion separated parenchymal and vascular cells. Endothelial cell markers CD31 and CLDN5 are shown with corresponding loading control TfR and GAPDH, respectively (1 mouse per lane, n=3 are shown, uncropped blot is shown in Supplementary Figure 5) . (**E, F**) Representative confocal images showing co-localization of ATV:TREM2 in brain tissue sections immunostained for Iba1 and a secondary antibody against human IgG. Images were taken from equivalent fields within the (**E**) neocortex and (**F**) hippocampus. (**G**, **H**) Quantification of human IgG (huIgG) signal localized within Iba1^{+} microglia. Individual microglia were segmented from confocal Z-stacks using Iba1 as a mask. The mean voxel intensity of huIgG was calculated by normalizing the sum intensity to the microglial volume from two image fields per sample. (n=4 mice/group except ATV-4D9-4hr (n=3), multiple unpaired-test, mean±SEM) **(I-K)** 6-week multi-dose study with equivalent ATV:4D9 and 4D9 brain exposure. **(I)** Antibody levels in brain are detected at comparable concentrations of ATV:4D9 (10 mg/kg) and anti-4D9 (100 mg/kg) 1 day post final dose (n=12 mice/group). **(J)** Representative images for Iba1 detected by IHC in brain after multi-dose of ATV:4D9, 4D9, or isotype controls. **(K)** ATV:4D9 but not 4D9 increases Iba1 area (n=12 mice/group, Brown-Forsythe and Welch ANOVA test compared to ATV:4D9 group, mean±SEM). **(L)** Soluble TREM2 is reduced in CSF (n=5 mice/group).

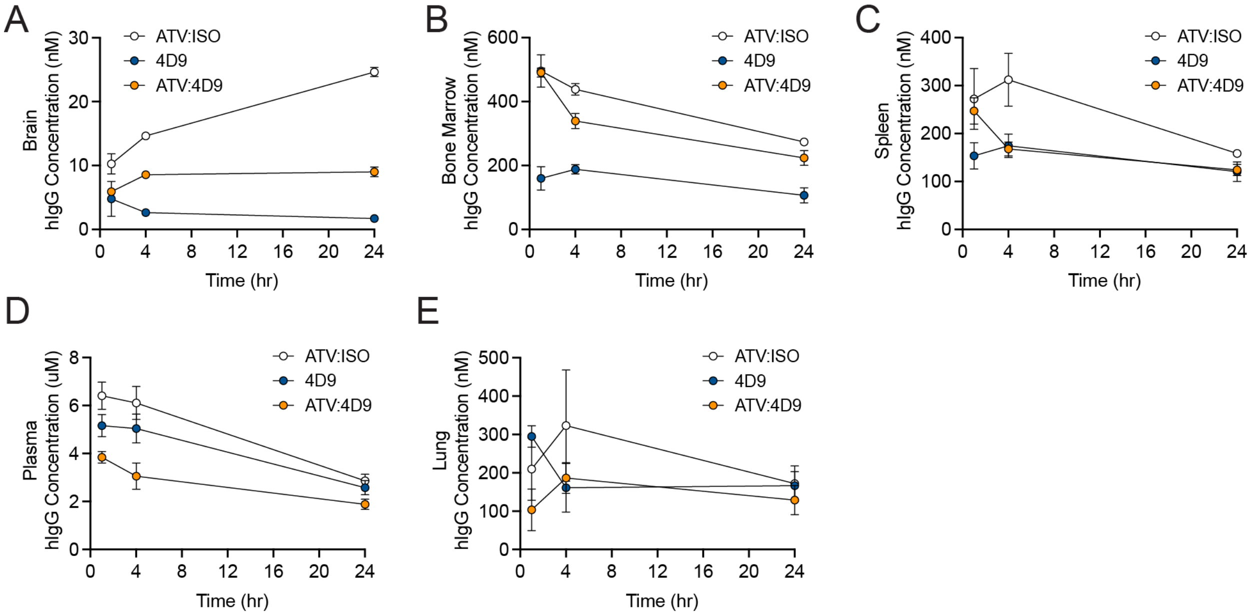

Supplementary Figure 2. Peripheral biodistribution of ATV:4D9 in WT; $TfR^{mu/hu}$ mice. 50mg/kg of ATV:4D9, ATV:ISO, and 4D9 were IV dosed in mice and tissues were collected and lysed at 1, 4, and 24h post dose. Antibody concentrations were quantified using an Fc:Fc sandwich ELISA (n=3-4 mice/group A-E). **(A)** ATV:4D9 and ATV:ISO are increased in the brain compared to 4D9. **(B)** Biodistribution to bone marrow was higher for ATV:4D9 and ATV:ISO relative to 4D9. **(C D, E)** Spleen, plasma, and lung concentrations were comparable across the three antibodies.

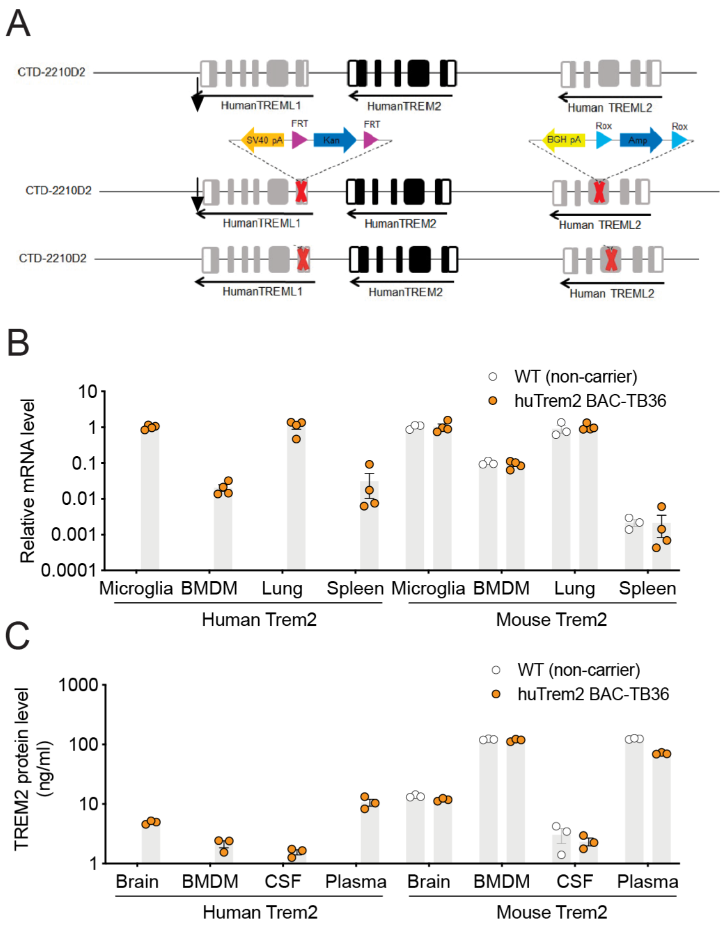

Supplementary Figure 3: Generation of a human TREM2 BAC transgenic mouse model for *in vivo* characterization of ATV:TREM2. (A) Diagram of genetic modifications to BAC CTD-2210D2. Human TREML1 exon 1 and TREML 2 exon 3 were replaced via homologous recombination by a SV40 pA Kan cassette and the BGH pA Amp cassette. TREML1 and TREML2 knockout was mediated by Kan cassette and the BGH pA Amp cassette deletion via FLP- and Dre-mediated recombination. The modified BAC CTD-2210D2 was used to generate the human TREM2 transgenic mouse model. **(B)** RT-qPCR analysis of human and mouse *Trem2* mRNA in microglia, bone marrow derived macrophages (BMDM), lung and spleen in human TREM2 tg mice and wild-type mice (n=3 miceWT/group, n=4 miceTREM2-BAC/group). (C) Human and mouse TREM2

proteins detected by MSD in brain, BMDM, CSF, and plasma in human TREM2 tg mice and wild-type mice (n=3 mice/group).

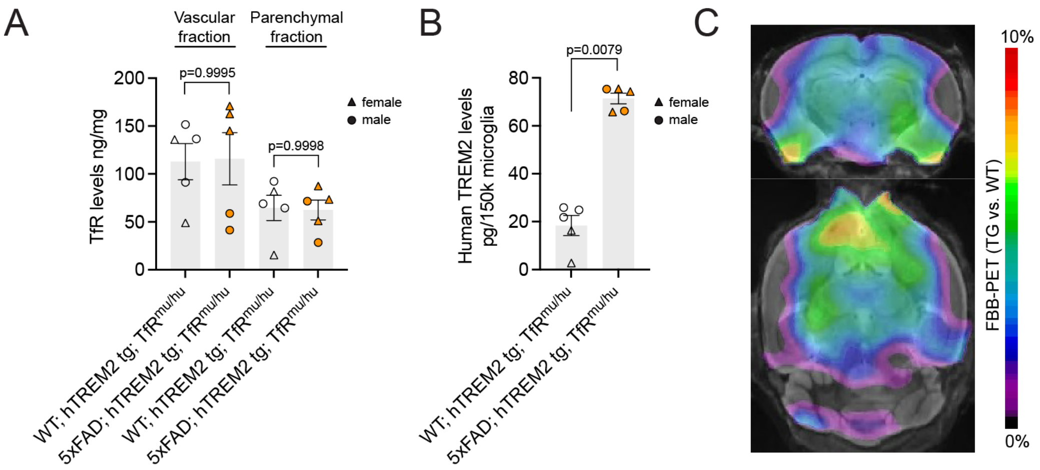

Supplementary Figure 4: Characterization of novel mouse models used in PET imaging studies. **(A)** TfR protein levels were measured by MSD in vascular and parenchymal brain fractions from WT; hTREM2 tg; $TfR^{mu/hu}$ or 5xFAD; hTREM2 tg; $TfR^{mu/hu}$ mice (n=5 mice/group, Tukey's multiple comparisons test, mean±SEM). (**B**) Human TREM2 protein levels were detected by MSD in isolated brain microglia from WT; hTREM2 tg; $TfR^{mu/hu}$ or 5xFAD; hTREM2 tg; $TfR^{mu/hu}$ mice. A total of 150,000 microglia were enriched by FACS (n=5 mice/group, Mann Whitney test, mean±SEM). (**C**) Regional alterations of [^{18}F]florbetaben (FBB)-PET (cerebellar scaling, 30-60 min p.i.) in an independent cohort of 5xFAD; hTREM2 tg; $TfR^{mu/hu}$ mice (n=4, 5 months old) compared to an age-matched wild type cohort (n=27, C57BL/6, 5 months old).

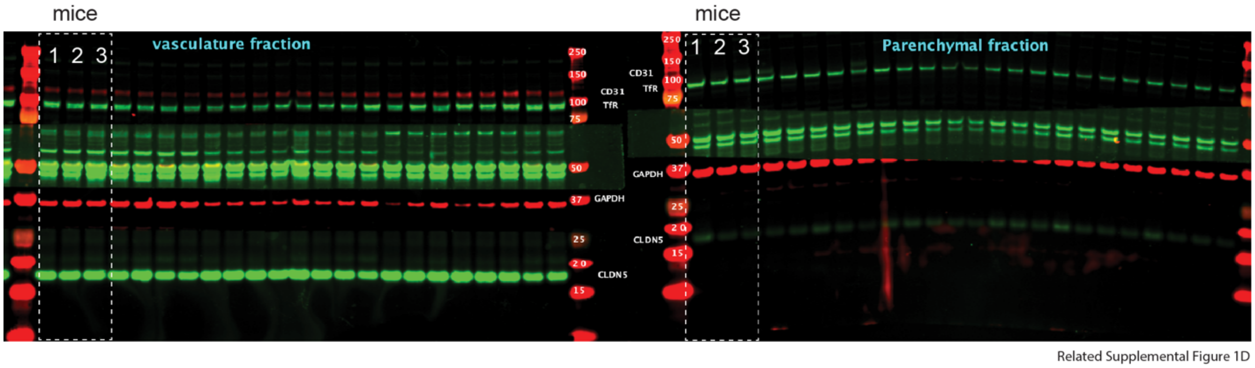

Supplementary Figure 5: Uncropped western blot image of capillary fractionation.

Dotted box represents the cropped area used for Supplementary Figure 1D.

SUPPLEMENTAL MATERIALS AND METHODS

Microglia imaging analysis

Fluorescent stained sections were captured using a Zeiss Axioscan.Z1 slide scanner with a 10x/0.45 NA objective. A custom macro in Zeiss Zen software was used to quantify signal for EdU and Iba1 following median smoothing, channel extraction, and local background subtraction to create binary masks of fluorescent signal for each channel. Objects smaller than 10 pixels were excluded as staining artifacts/debris. The whole brain region was automatically outlined for each section and analyzed using the macro to measure the number and area of total microglia coverage ($Iba1^+$) and newborn microglia (EdU^+ and $Iba1^+$) The percentage $Iba1^+$ in the areas of interest were calculated by normalizing the positive pixel area to the quantified tissue area and averaged from 3 sections/animal.

To assess microglia morphology changes during ATV:4D9 treatment, confocal images of cortical regions of Iba1 stained mouse brain sections were acquired with a spinning disk confocal microscope (Zeiss AxioObserver Z1, Carl Zeiss Microscopy). A minimum of 3 regions per animal were imaged in 3 animals per treatment using a 40x/1.3 NA oil immersion objective at a pixel size of 333 nm x 333 nm. Confocal z-stacks of 15-30 μm with a z-step size of 250 nm were acquired for each channel.

Microglia were initially segmented using a voxel U-net neural network pipeline[2] which was trained to simultaneously predict the location of the microglia cell body and soma from the Iba1 signal, down-sampled to approximately 1.0 x 1.0 x 1.0 μm isotropic volume images. The microglia cell mask was accepted at a 90% confidence threshold, while the soma was accepted at 50% confidence. The cell mask was then separated into individual microglia by first splitting the mask into 4-connected components, then splitting each of

those components by distance to the nearest soma using a watershed segmentation[3]. The final cell masks were then up-sampled to full image resolution and refined using a restricted segmentation approach as follows. First, a core mask was calculated by eroding the initial cell mask twice, and a shell mask calculated by dilating the initial cell mask once. Next, the core voxels were assigned to the final mask, regardless of their Iba1 intensity values. Finally, any voxels outside the core, but inside the shell inclusive were assigned to the final mask if they were above a manually determined Iba1 intensity threshold (4,000 AU for all images). Microglial morphology was analyzed using custom python code built on the tools in the scikit-image package[3] and the skan skeletal analysis package[4] (see "Code Availability" for detail). Specifically, geometric features such as volume, surface area, moments of inertia, and dimensionless ratios of these were calculated for the microglial volume, the convex hull of the microglial volume, and the axis-aligned bounding box of the microglial volume. Skeletal features were calculated by calculating the one-voxel thick morphological skeleton of the microglial volume, then using graphical analysis to calculate properties such as total number of branchpoints, branch length distributions, and numbers of total, short (less than 5 μm long) and long branches (longer than 20 μm). Fractal features were calculated using a box-counting approach using 12 octaves of boxes that began at the resolution limit of the microglial surface and doubled in size with each octave, where several measures of each fractal feature were calculated using both log-linear regression coefficients and the average of the fractal value measured at each octave[5]. Sholl-like features were calculated using the soma segmentation where available, or a surface touching sphere centered on the center of mass of the microglia for microglia without a predicted soma but were extended to

calculate all features using concentric spherical shells around each soma as opposed to concentric circles, with all subsequent features measured in three dimensions as opposed to the typical two. Immunohistochemistry features were calculated by measuring the mean, median, standard deviation, and inner-quartile range of the signal within the total microglial volume, the microglial core, and the microglial surface. A full list of features measured for each microglia is given in Supplementary Table 6.

The 65 features for all microglia across all treatment conditions were concatenated into a single matrix, and each column was standardized using the QuantileTransformer in scikit-learn [6]. Microglia with volumes less than 500 μm^3 or more than 5,000 μm^3 were excluded as these were typically fragmented and multi-cell segmentations respectively. The feature matrix was projected onto 8 principal components which collectively explained >90% of the total variance. Uniform manifold approximation and projection (UMAP) was used to visualize the distribution of cells in PCA space, revealing two visible groupings of cells. K-means clustering was performed with between 2 and 10 clusters, where the peak silhouette score (0.8180) occurred at 2 clusters. To assign phenotypes to clusters, the fold change difference between the two clusters was calculated for each standardized feature, and all features with an FDR-corrected p value < 0.05 and an effect size (Cohen's d) > 0.2 were ranked in order of difference between the means. Top features representative of clusters 1 and 2 were examined by experts on glial biology and labels for the clusters assigned. Cells were then grouped by treatment, and the average percentage of each cell in each cluster for each treatment were compared to the control using a 1-way ANOVA followed by FDR corrected post-hoc tests.

Myelin debris and Aβ fibrils preparation, fluorescent labeling for ex-vivo phagocytosis assay

To isolate myelin debris, fresh adult mouse brain was chopped and homogenized with Lysis Buffer containing 10 mM HEPES, 5 mM EDTA, 0.32 M sucrose and protease inhibitors. Brain homogenate was transferred to ultracentrifuge tube (Beckman Coulter, 344060) and sequentially underlaid with equal amount of 0.32 M sucrose (10 mM HEPES and 5mM EDTA) and 0.85 M sucrose (10 mM HEPES and 5 mM EDTA) and then centrifuged at 75,000 x g for 30 min at 4°C with low deceleration and acceleration. Crude myelin was enriched from 0.32 M and 0.85 M sucrose interface and placed in a clean ultracentrifuge tube and washed twice with ice cold water and centrifuged at 75,000 x g for 15 min and 12,000 x g for 10 min at 4°C sequentially with max acceleration and deceleration. The water wash step was then repeated. Afterward the pellet was resuspended in lysis buffer and the whole process was repeated from the first underlay step. The final myelin pellet was resuspended in 1XPBS and tested for protein and cholesterol content. Myelin was diluted to 1 mg/ml and aliquoted and stored at -80°C before labeling.

Myelin was further labeled with pHrodo-green using the pHrodo iFL Green Microscale Protein Labeling Kit (Invitrogen, P36015). 100 ug of myelin was diluted in 100 uL of 1XPBS per labeling reaction. 10 uL of 1 M sodium bicarbonate and 2.5 uL of pHrodo iFL Green STP ester were added to each labeling reaction and incubated at RT for 30min. After 3 times wash with 1xPBS, the pHrodo-green labeled myelin was resuspended in 100ul 1XPBS and stored at -80°C for phagocytosis assay.

FAM-labelled Aβ (1-42) peptides were purchased from Anaspec (AS23525-05). For preparation of aggregated fibrils, 0.5 mg of FAM-Aβ was reconstituted in 100 uL of DMSO then diluted by adding 1 mL of 1XPBS and incubated at 37°C for 1 day at 700rpm in an orbital shaker. The solution was then transferred to an ultracentrifuge tube (Beckman 357488) and spun at 100,000 x g for 30 min at 4°C. The supernatant was discarded and the pellet was resuspended and washed with 1ml of 1XPBS 3 times. The final pellet was resuspended in 100 uL of 1XPBS and stored at -80° C.

Human TREM2 antibody discovery

Lewis Rats (Charles River Laboratories) were immunized with the recombinant TREM2 protein (19-172aa) with a c-terminal His (x6)-tag bi-weekly over 5 weeks. Lymph nodes (brachial, axillary, inguinal, and popliteal) were collected from rats with significant TREM2 titers. Cells from these tissues were isolated and enriched for plasma cells using flow cytometry. Enriched plasma cell suspension was injected into AbCellera Biologics Inc.'s microfluidic screening devices with over 90,000 individual nanoliter-volume reaction chambers (1-9). Single cells secreting TREM2-specific antibodies were identified and isolated using a cell-binding assay followed sequentially by a bead-binding assay (10-11). The cell-binding assay comprised HEK293 readout cells expressing human TREM2, HEK293F cells expressing mouse TREM2, and a HEK293 control cell line. The bead-binding assay comprised beads conjugated with human TREM2 recombinant protein. The readout cells and beads were flowed sequentially into microfluidic screening devices, incubated with single antibody-secreting cells, and mAb binding to cognate antigens was detected via a fluorescently labeled anti-rat IgG secondary antibody. Positive hits were identified using machine vision and recovered using automated robotics-based protocols.

Single-B-cell antibody sequencing and bioinformatic analysis

Single cell polymerase chain reaction (PCR) and custom molecular biology protocols generated NGS sequencing libraries (MiSeq, Illumina) using automated workstations (Bravo, Agilent). Sequencing data were analyzed using a custom bioinformatics pipeline to yield paired heavy and light chain sequences for each recovered antibody-secreting cell (12). Each sequence was annotated with the closest germline (V(D)J) genes and degree of somatic hypermutation. Antibodies were considered members of the same clonal family if they shared the same inferred heavy and light V and J genes and had the same CDR3 length.

Recombinant antibody generation

The standard IgG anti-TREM2 antibody containing the effector knock-out substitutions was generated by cloning the variable domains of the TREM2 antibody into the human IgG1 L234A/L235A (LALA), and human kappa chain expression vectors. To generate ATV:TREM2 in either bivalent or monovalent Fab format, the Fc domain was replaced by the engineered Fc sequence[7] with the LALA effector knock-out substitutions.

Antibody expression and purification

Anti-TREM2 antibody, ATV:TREM2 and isotype controls were expressed via transient transfection of Expi293F cell line (RRID:CVCL_D615, Thermo Fisher Scientific A14527) adapted to BalanCD HEK293 media (Irvine Scientific) according to manufacturer's instructions. Cultures were co-transfected with plasmids encoding for standard

monoclonal antibody (2-chain): 1:1 Heavy Chain (HC):Light Chain (LC) and antibody transport vehicle (ATV) molecule: 1:1:2 Knob:Hole:Light Chain (LC).

Anti-TREM2 antibody, ATV:TREM2 and isotype controls were purified to homogeneity from serum-free BCD293 cultures by a series of chromatographic steps. Supernatants were loaded onto a 1x PBS equilibrated HiTrap MabSelect PrismA affinity column (Cytiva using an Akta Pure System), the column was then washed with 5 column volumes (CVs) of 1x PBS and 0.1% Triton X-100, followed by 10 CV of 1x PBS wash. Bound proteins were eluted using 0.1 M sodium citrate pH3.6 and 150 mM sodium chloride. Immediately after elution, Protein A eluate was neutralized to pH6.5 with 1M Tris pH8. Neutralized Protein A eluate was conditioned with 50mM sodium acetate pH6.5 prior cation-exchange chromatography. Use linear gradient with, 0.5M sodium chloride and 50mM sodium acetate pH6.5 to elute proteins from SP HP resin (Cytiva). Final fractions with a high degree of purity, as assessed by analytical size-exclusion chromatography (SEC) and/or microcapillary electrophoresis (Caliper), were pooled, concentrated and dialyzed into formulation buffer of 10mM sodium acetate pH5.5, 6% sucrose and/or 1x PBS for cell-based functional assay. Preparations were stored at $4^{o}C$ or $-80^{o}C$ prior to use and routinely analyzed by SEC, SPR and for endotoxin content.

Affinity and binding kinetics

Human TREM2 binding affinities of anti-TREM2 antibodies were determined by surface plasmon resonance using a BIAcore 8K instrument (GE Healthcare). Biacore Series S CM5 sensor chip was immobilized with a mixture of two monoclonal mouse anti-Fab antibodies (Human Fab capture kit from GE Healthcare) to capture antibodies for binding measurements. To measure TREM2 binding affinities of anti-TREM2 antibodies, serial 3-

fold dilutions of recombinant human TREM2 protein were injected at a flow rate of 30 μL/min for 300 seconds followed by 600 second dissociation in HBS-EP+ running buffer (GE, #BR100669). For human TfR binding affinity measurements, serial 3-fold dilutions of recombinant human TfR apical domain were injected for 60 seconds followed by 60 seconds of dissociation. After each injection, the chip was regenerated using a 50mM glycine pH2.0 regeneration buffer. A 1:1 Languir model of simultaneous fitting of k_{on} and k_{off} was used for kinetics analysis.

***In vitro* biochemical shedding blocking**

Full length -peptide "biotin-DPLDHRDAGDLWFPGESESFEDAHVEHSISRSLLEGEIPFPPC-FAM" was custom synthesized by Elim Biopharm, Inc. Lyophilized peptides were resuspended in DMSO as 1 mM stock solution and further diluted to 1 μM working solution in FP assay buffer containing 25 mM Tris (Neknova, T5080), 2.5 μM ZnCl2 (Acros organics, 196840010), 0.005% Brij-35 (Thermofisher Scientific, 20150) and adjusted to pH 7.5 with hydrochloric acid (Fisher chemical, SA431-500). A reaction mixture containing FL-peptide, streptavidin, and testing antibodies were pre-mixed in FP assay buffer and incubated at room-temperature for 30 min. Reconstituted ADAM17/TACE enzyme solution (R&D systems, catalog #930-ADB-010) was then added to the mixture that has a final concentration of 20 nM FL-peptide, 0.1 mg/mL Streptavidin (Thermofisher Scientific, 21125), 200 nM testing antibodies, and 25 ug/mL ADAM17/TACE enzyme in 20 μL reaction volume. The reaction mixture was incubated at 25°C for 24 h. The pan matrix metalloproteinases inhibitor GM6001 (Adooq Bioscience, A13320-50) was used at 50 μM in the assay reaction. No enzyme control representing theoretical 0% cleavage was setup

the same way as assay reaction without the addition of ADAM17/TACE. Short-peptide "SISRSLLEGEIPFPPC-FAM" which corresponds to the cleaved fragment was used as control for 100% cleavage. Short-peptide control was diluted in FP assay buffer to have 20 nM final concentration. 10 μL reaction product was diluted with 190 μL FP assay buffer. 30 μL of the diluted reaction was transferred to a 384-well assay plate (Corning, catalog #3575) and measured by a plater reader equipped with FP Fluorescein Dual detector (Perkin Elmer, EnVision, serial # 1041585). The mP value was computed by the EnVision Workstation software (Version #1.14.3049.528) and further analyzed in R Studio and GraphPad Prism. Raw FP signal (mP value) with technical repeats was averaged for each experiment. A total of 4 independent experiments were performed. Percent peptide cleaved was calculated based on **Equation 1**:

$$\%\,peptide\ cleaved\ =\ 100\% - \frac{X - FP_{min}}{FP_{max} - FP_{min}} \qquad \text{[Equation 1]}$$

X, FP_{min}, FP_{max} represents FP value measured from experimental group, short-peptide control, and no enzyme control, respectively. Unpaired t-test were used to compare statistical differences between groups.

RNA sequencing in isolated mouse brain microglia

Sample Preparation

Mice were perfused with cold PBS and cortical and hippocampal tissues were dissected and dissociated into a single cell suspension using the Adult Brain Dissociation Kit (Miltenyi Biotec 130-107-677), according to the manufacturer's protocol.

scRNA-seq library preparation in WT mice

Single cell suspensions were enriched for immune cells via MACS sorting with Miltenyi CD45 microbeads, mouse (Miltenyi Biotec, 130-052-301). Single cell suspensions were prepared in 3 batches, each batch containing one sample of each experimental group. Sorted cells were subjected to droplet-based single-cell RNA sequencing using the Chromium Single Cell 3' GEM, Library & Gel Bead Kit v3 per the manufacturer's instructions (10x Genomics, CG000183 Rev B) targeting a recovery of 10,000 cells per sample. Next-generation sequencing was performed by SeqMatic (Fremont, CA) on an Illumina NovaSeq instrument with a S2 flow cell generating paired-end reads (28x8x90 bases).

scRNA-seq library preparation in AD mouse model

Four batches of single cell suspensions, with one replicate of each condition, were labeled with CD11b (BioLegend 101251) and CD45 antibody (BD Biosciences 559864) and cell multiplex oligos per user guide (10X Genomics, CG000391 Rev A). After 3-4 washes at 500 x g for 5 minutes in 4C, cells were filtered through a 30 µm strainer and counted manually on a hemocytometer. Equal proportions of cells from each sample were combined into one pool per batch and 100,000 live, CD11b+ cells were FACS sorted on FACS Aria III (BD Biosciences with a 100 µm nozzle) into 1% BSA in PBS. The multiplexed pool was counted on a hemocytometer after centrifugation at 200 x g for 10 minutes in 4C to pellet cells and remove excess liquid leaving behind enough volume for a loading concentration of 1000-2000 cells/µL. Single cell capture and library preparation was performed using Chromium Next GEM Single Cell 3' Kit v3.1 per user guide (10X genomics, CG000388 Rev A). After cDNA amplification of RT materials from captured

cells, transcriptional cDNA was separated from cell multiplex oligo cDNA via NucleoMag bead purification (Takara, 7444970.50), proceeded by library preparation of both sets of cDNA. Transcriptional libraries were pooled in equimolar for a sequencing run on an Illumina MiSeq instrument to estimate the number of cells captured in each library. This MiSeq capture information was used to pool libraries in equal proportions for similar sequencing coverage of 50,000 reads per cell across libraries and 5000 reads per Cell multiplex oligo. Sequencing on the Illumina NovaSeq 6000 instrument generating paired-end reads (28x10x10x90 bases) was performed by SeqMatic (Fremont, CA).

Sample-level data processing, QC, and filtering

Sample demultiplexing, alignment, and per-barcode expression quantification, and empty droplet detection was performed against *mus musculus* genome mm10 using Cell Ranger (v7.0.0 for WT study; v6.1.1 for AD study) using default parameters. For the WT study, the “cellranger count” pipeline was used. For the AD study, the “cellranger multi” pipeline was used to perform cell multiplex hash demultiplexing. Low quality droplets were further filtered based on 3 criteria: total number of genes detected (nFeature), total numbers of UMIs, and percentage of counts mapping to mitochondrial genes. For WT study, cutoffs of ≥500 nFeature, ≤25000 UMIs, and ≤10% mitochondrial reads were used. For the AD study, cutoffs of ≤60000 UMIs and ≤7.5% mitochondrial reads were used. Individual objects were generated for each study by combining all samples within each sequencing batch and then using the integration pipeline in the Seurat v4[8]. Batches were integrated to remove batch effects using the “FindAnchors” and “IntegrateData” functions. Following integration, data normalization, data scaling, PCA, UMAP projection, and unbiased clustering were performed to identify and exclude non-microglial cell types based on

marker gene expression. These steps were carried out using the “NormalizeData”, “ScaleData”, “FindVariableFeatures”, “ScaleData”, “RunPCA”, “RunUMAP”, “FindClusters”, and “FindAllMarkers” functions in the “Seurat” package, respectively[9-13].

Bioinformatic Analysis

Combined analysis was performed using the standard Seurat v4 workflow. To reduce the total size of the data object, a random sample of 5000 cells was taken from each experimental group in Study 1 for use in the combined analysis. The two studies were then combined using the “FindAnchors” and “IntegrateData” functions in the “Seurat” package to remove study-specific effects on dimensionality reduction and clustering. Data were then normalized using “NormalizeData.” The top 2000 variable genes excluding those commonly thought of as being associated with dissociation stress[14] were used to perform PCA using “RunPCA.” The top 9 principal components were used to perform UMAP dimensionality reduction, shared neighest neighbor graph construction, and Louvain clustering (“RunUMAP”, “FindNeighbors”, “FindClusters”). For differential expression analysis, pseudobulk libraries were generated by summing counts across samples:clusters using the “AggregateAcrossCells” function in the “scuttle” package[15]. Differential expression was then performed using the limma/voom workflow[16]. A linear model was fit for each gene to find genes that differed between clusters with the “study” added to the model as a fixed effect. GSEA for each cluster was performed using the “fgsea” and “sparrow” packages[17] (https://github.com/lianos/sparrow) with the moderated t-statistic from differential expression testing vs the “Homeostatic” cluster used as the gene ranking statistic. Gene sets for GSEA were taken from the hallmark gene sets in the Molecular Signatures Database (MSigDB)[18].

RNAseq of iMG dosed with ATV:TREM2 or other stimuli

iMG were plated at 30,000 cells/well in microglia differentiation media (see below) on 96-well Cell Carrier Ultra plates and allowed to adhere for 3 days. Subsequently, media was exchanged for microglia differentiation media diluted 1:3 with microglia differentiation media without growth factors and treated with 100nM ATV:ISO or ATV:TREM2, or 10 ng/mL LPS (Sigma L2880), 20 ng/mL TGFb (RnD 7754-BH), or 20 ng/mL IFNg (RnD 285-IF/CF). After 4 days of treatment, RNA was extracted using the RNeasy Plus Micro Kit (Qiagen, 74034). RNA quality was assessed with a RNA 6000 Pico chip (Agilent 5067-1513) on a 2100 Bioanalyzer (Agilent) and quantified with Qubit RNA HS Assay Kits (Life Technologies, Q32855).

Bulk RNA-seq library preparation of iMG

Bulk RNA-seq libraries were generated using QuantSeq 3' mRNA-seq Library Prep Kit FWD for Illumina (Lexogen A01173) with the UMI second strand synthesis module in order to identify and remove PCR duplicates, following the protocol defined by the manufacturer. Library quantity and quality were assessed with Qubit™ 1X dsDNA HS Assay Kits (Invitrogen Q33231) and Bioanalyzer High Sense DNA chip (Agilent 5067-4626). Libraries were combined in equimolar ratios into one sequencing pool. Next-generation sequencing of 75 bp, single end reads were generated on an Illumina NovaSeq instrument with a SP flow cell at SeqMatic (Fremont, CA).

Bulk RNA-seq data processing and analysis

Raw FASTQ files were aligned to the genome using the STAR aligner [19] and summarized into gene-level counts using *featureCounts* from the *subread* package[20] as previously described[21]. Lowly expressed and non protein-coding genes were removed, and

differential expression analysis was performed using the limma/voom pipeline[22]. Linear models were constructed to identify genes differentially expressed between the groups of interest. “Takedown day” was encoded as a fixed effect to account for potential technical artifacts induced by processing animals on different days. We used gene set enrichment analysis (GSEA) to summarize individual differential gene expression results at the pathway/signature level[17]. Gene sets were taken from the hallmark molecular signature database[18]. GSEA statistics were generated using the fgseaMultiLevel function in the fgsea R package using the moderated t-statistic as the gene ranking statistic[17]. Gene set definitions and GSEA statistics for all comparisons are provided in Supplementary Table 4. All software versions for the RNA-seq analysis correspond to Bioconductor release version 3.13[23]. Individual genes were selected to show pathway activity in the Fig. 3K and Fig. 5M heatmaps by extracting the top 10-20 genes found in the “leading edge” of each pathway when ranked by their individual t-statistics.

Generation and cell culture of human iPSC-derived microglia

Hematopoietic Differentiation

Human iPSCs (RRID:CVCL_D086, Gibco A18945) were maintained in mTESR-Plus (Stemcell Technologies #100-0276) until seeding for differentiation. At ~80% confluence, cells were singularized with TrypLE Express (ThermoFisher #12604013) for five minutes and mechanically dissociated with a P1000 tip using mTeSR-Plus and transferred to a 15 mL conical tube, and pelleted at 300 x g for five minutes. Cells were resuspended in mTeSR-Plus and counted using a Nexcelom cellometer and seeded into mTeSR-Plus + 10uM y-27632 (Tocris, 1254) at 13,200 cells/cm^2 (approximately 50,000 cells per well of a 12-well tissue culture plate). All media used for hematopoietic differentiation was from

Stemcell Technologies STEMdiff Hematopoietic Kit (#05310). On day 0 mTeSR-Plus+y-27632 was aspirated and replaced with 1.0 mL medium A. On day 2 0.5 mL medium A was removed from each well and 0.5 mL fresh medium A was added. On day 3 all medium A was removed from each well and fresh medium B was added. On day 3 primary human astrocyte feeder cells were thawed and seeded into poly-L-Lysine coated 10cm^2 tissue culture dishes using Lonza AGM (CC-3186) as growth medium. This is done so cells are ready to serve as feeder cells by day 12. On days 5 and 7 0.5 mL medium B is removed from each well and 0.5 mL fresh medium B is added back to the well. On day 8 primary human astrocytes were dissociated using 0.05% Trypsin-EDTA (ThermoFisher, 25300062) counted in the Nexcelom cellometer and seeded at 10,000 cells/cm^2 (approximately 100,000 cells per well of a 6-well tissue culture plate was used). On day 9 no medium was removed, but 0.5mL medium B was added to each well. On day 10 floating colonies of hematopoietic progenitor cells (HPCs) were visible and collected. To collect the cells, medium from each well was collected and added to a 15 mL conical tube. Cells were briefly mechanically dissociated with a serological pipet and tubes were transferred to the centrifuge and pelleted at 300 x g for five minutes. 0.5 mL fresh medium B was added to each well of the 12-well plate. After pelleting HPCs, 0.5 mL of conditioned medium from each tube was added back to each well of the 12-well plate and the plate was returned to the incubator. Any remaining medium in the conical tube was then aspirated and HPCs were resuspended in microglia differentiation medium (IMDM base medium containing 10% FBS, and 20ng/mL each of GM-CSF (Peprotech, AF-300-03), IL3 (Peprotech, AF-200-03), and M-CSF (Peprotech, AF-30-25). AGM was aspirated from the astrocyte feeder cell plates and HPCs collected from an entire 12-well plate were

seeded evenly across 2, 6-well plates containing astrocyte feeder cells (bring total microglia differentiation medium volume to 2 mL/well). On day 12 the same steps fromdDay 10 were repeated and the cells were seeded evenly across the same 6-well plates already containing HPCs and astrocyte feeder cells (each well now contains 4 mL/well). After one week of culture numerous cells are seen proliferating in suspension with some firmly attaching to astrocyte feeder cells. *Note*: A small fraction of HPCs was collected at day 10 and day 12 to ensure that at least 80% of cells are $CD43^+$ by flow cytometry analysis.

Microglia Differentiation and Maintenance

On day 20 2 mL medium was collected from each well and suspension cells were pelleted at 300 x g for five minutes. Medium was aspirated and pelleted cells were resuspended in 24 mL microglia differentiation medium (see above) and 2 mL of the cell suspension was added back to each well. This process is repeated ~ every 5 days as needed to maintain a consistent pH in the medium (phenol red indicated). By day 42 cells were harvested and seeded in an assay dependent manner.

Small animal PET/MRI

All rodent PET procedures followed an established standardized protocol for radiochemistry, acquisition times and post-processing[24], which was transferred to a novel PET/MRI system[25]. All mice were scanned with a 3T Mediso nanoScan PET/MR scanner (Mediso Ltd, Hungary) with a triple-mouse imaging chamber. Two 2-minute anatomical T1 MR scans were performed prior to tracer injection (head receive coil, matrix size 96 × 96 × 22, voxel size 0.24 × 0.24 × 0.80 mm³, repetition time 677 ms, echo time 28.56 ms, flip angle 90°). Injected dose was 12.3 +/- 2.2 MBq for [^{18}F]GE-180 (TSPO) and 14.5 +/-

3.4 MBq [^{18}F]FDG (glucose) delivered in 200 µl saline via venous injection. PET emission was recorded in a dynamic 0-90 min window for TSPO PET and in a dynamic 0-60 min window for FDG PET. List-mode data within 400-600 keV energy window were reconstructed using a 3D iterative algorithm (Tera-Tomo 3D, Mediso Ltd, Hungary) with the following parameters: matrix size 55 × 62 × 187 mm³, voxel size 0.3 × 0.3 × 0.3 mm³, 8 iterations, 6 subsets. Decay, random, and attenuation correction were applied. The T1 image was used to create a body-air material map for the attenuation correction.

We studied PET images of WT; hTREM2 tg; $TfR^{mu/hu}$ mice and 5xFAD; hTREM2 tg; $TfR^{mu/hu}$ mice (n=6 per antibody and placebo group). Framing was 6x10s, 6x30s, 6x60s, 10x300s for FDG-PET and 6x10, 2x30, 3x60, 5x120, 5x300, 5x600 for TSPO-PET.

Normalization of TSPO PET data was performed by the previously validated myocardium correction method for the previously established 60-90 min time window[26,27], after cross validation against volume of distribution (V_T) images obtained from the full dynamic scan. We generated V_T images with an image derived input function[28,29] using methodology as previously described[30]. The plasma curve was obtained from a standardized bilateral VOI placed in both carotid arteries. A maximum error of 10% and a V_T threshold of 0 were selected for modelling of the full dynamic imaging data. Late static myocardium corrected TSPO-PET data were used and reported due to less methodological variance, which was proven to be beneficial in serial small animal PET imaging[26]. Normalization of FDG-PET was performed by standardized uptake values (SUVs), reflecting the common read-out in clinical setting. Blood-flow adjusted validation of FDG-PET quantification was performed by a simplified reference tissue modeling approach, using the periaqueductal grey as an established reference tissue[31]. The reference tissue was validated by analyzing antibody

and vehicle injected mice using V_T images as described above which confirmed no V_T differences between study groups between groups in the periaqueductal grey. A predefined forebrain volume-of-interest (comprising 19.4 mm^3) was delineated by cortical, striatal and hippocampal regions of the Mirrione atlas[32] and served for extraction of TSPO-PET and FDG-PET values for all mice.

An independent cohort of age-matched 5xFAD; hTREM2 tg; $TfR^{mu/hu}$ mice (n=4) received [^{18}F]florbetaben β-amyloid-PET imaging (13.3 +/- 1.0 MBq, 30-60 min p.i.) to obtain the 3-dimensional pattern of fibrillar amyloidosis for the acute dosing study. Cerebellar scaled images of 5xFAD; hTREM2 tg; $TfR^{mu/hu}$ mice were compared to an age-matched in-house cohort of n=27 C57BL/6 wild type mice with equivalent acquisition and normalization. Regional alterations (%) of the amyloid-PET signal in both mouse models were determined in all regions of interest of the Mirrione atlas. Similarly, regional alterations of TSPO-PET (SUV_H) and FDG-PET (SUV) in ATV:TREM2 versus isotype control treated 5xFAD; hTREM2 tg; $TfR^{mu/hu}$ mice were determined at day 8 and a correlation analysis was performed to test if ATV:TREM2 treatment changes in TSPO-PET and FDG-PET correlate with the regional detection of amyloidosis.

Twelve-week repeat dose study in WT; $TfR^{mu/hu}$ mice

ATV:4D9, 4D9, and vehicle were dosed via intraperitoneal injections once a week for 12 weeks (13 total doses) to WT; $TfR^{mu/hu}$ mice with 10 mg/kg in each group. Necropsy was performed after euthanization by exsanguination from the abdominal aorta after isoflurane anesthesia 1 day after the final dose. Histopathological analyses were performed according to standard procedures.

Biodistribution of ATV:4D9 and ATV:TREM2

To determine the biodistribution of ATV:4D9, WT; $TfR^{mu/hu}$ mice were IV dosed with a single dose of 50 mg/kg of ATV:4D9 or ATV:ISO or 4D9. Plasma, brain, lung, liver, spleen, and bone marrow was collected at 1, 4, and 24 h post-dose to quantify huIgG concentrations. To determine the biodistribution of ATV:TREM2, WT; hTREM2 tg; $TfR^{mu/hu}$ mice were IV dosed with a single, high dose of 50 mg/kg of anti-TREM2 or ATV:TREM2 to maximize signal to noise and tissues were collected at 4 h or 24 h post-dosing for evaluation of huIgG concentrations in brain and capillary depletion fractions and huIgG localization in brain using immunohistochemistry. N=3-4 mice were included per treatment group and time point.

Liquid chromatography-mass spectrometry (LCMS) analysis

Lipid levels were analyzed by liquid chromatography (Shimadzu Nexera X2 system, Shimadzu Scientific Instrument, Columbia, MD, USA) coupled to electrospray mass spectrometry (QTRAP 6500+, Sciex, Framingham, MA, USA). For each analysis, 5 µL of sample was injected on a BEH C18 1.7 µm, 2.1×100 mm column (Waters Corporation, Milford, Massachusetts, USA) using a flow rate of 0.25 mL/min at 55°C. For positive ionization mode, mobile phase A consisted of 60:40 acetonitrile/water (v/v) with 10 mM ammonium formate + 0.1% formic acid; mobile phase B consisted of 90:10 isopropyl alcohol/acetonitrile (v/v) with 10 mM ammonium formate + 0.1% formic acid. For negative ionization mode, mobile phase A consisted of 60:40 acetonitrile/water (v/v) with 10 mM ammonium acetate; mobile phase B consisted of 90:10 isopropyl alcohol/acetonitrile (v/v) with 10 mM ammonium acetate. The gradient was programmed as follows: 0.0–8.0 min from 45% B to 99% B, 8.0–9.0 min at 99% B, 9.0–9.1 min to 45% B, and 9.1–10.0 min at 45% B. Electrospray ionization was performed in either positive or negative ion mode

applying the following settings: curtain gas at 30; collision gas set at medium; ion spray voltage at 5500 (positive mode) or 4500 (negative mode); temperature at 250°C (positive mode) or 600°C (negative mode); ion source Gas 1 at 50; ion source Gas 2 at 60. Data acquisition was performed using Analyst 1.6.3 (Sciex) in multiple reaction monitoring mode (MRM), with the following parameters: dwell time (msec) and collision energy (CE); declustering potential (DP) at 80; entrance potential (EP) at 10 (positive mode) or -10 (negative mode), and collision cell exit potential (CXP) at 12.5 (positive mode) or -12.5 (negative mode). Lipids were quantified using a mixture of non-endogenous internal standards. Lipids were identified based on their retention times and MRM properties of commercially available reference standards (Avanti Polar Lipids, Birmingham, AL, USA).

pSyk activity with lipid ligand (liposome) co-stimulation

Liposome preparation: Liposomes were prepared as follows: 7 mg DOPC (1,2-dioleoyl-*sn*-glycero-3-phosphocholine, Avanti 850375) and 3 mg POPS (1-palmitoyl-2-oleoyl-*sn*-glycero-3- phospho-L-serine, Avanti 840034), were combined in chloroform in a glass vial and dried under a stream of N2 gas for 1h. The lipid mixture was re-suspended in 1 mL PBS (10 mg/mL final lipid concentration) and vortexed for 2-3 min until lipids were in solution, then extruded to form small unilamellar vesicles using an Avanti mini-extruder (Avanti 610023) containing a 100 nm membrane. Cells were plated on PDL-coated 96-well plates. After 24 h at 37ºC, the media was removed and cells were washed once with PBS. Cells were dosed with 100 nM ATV:TREM2 antibody (or isotype control) pre-mixed with liposomes (1 mg/mL final concentration in PBS), or antibody in PBS. Cells were incubated for 5 min at 37ºC, and lysed in CST (Cell Signaling Technology, 9803) lysis

buffer containing 1 mM PMSF (phenylmethylsulfonylfluoride, Sigma Aldrich). Lysates were assessed for pSyk levels using AlphaLisa.

Super-resolution microscopy and quantification of TMRE-stained iMG dosed with ATV:TREM2

iMG (20,000 cells/well) were seeded on PDL-coated 96-well Agilent Seahorse XF Cell Culture microplate in microglia differentiation media. After 24 h, media was changed to fresh microglia differentiation media containing 100 nM ATV:TREM2 or ATV:ISO. Cells were incubated for 3 days, then media was removed and replaced with Live Cell Imaging Solution (Invitrogen, A14291) containing 10 nM TMRE (Abcam, ab113852) for 20 min. Cells were then washed once with Live Cell Imaging Solution, then imaged with a laser scanning confocal microscope (Leica SP8; Leica Microsystems, Inc), acquired with a 40x/1.3 NA oil objective in LIGHTNING super-resolution mode at a pixel size of 36 nm and images were processed using an adaptive processing algorithm. The representative images were generated by three-dimensional reconstruction in Imaris (Bitplane, V9.9.1). To identify different classes of mitochondrial morphology, we segmented individual mitochondrial surfaces by thresholding on the TMRE fluorescence intensity. Morphological classes were defined by using object volume and the long axis of the object-oriented bounding box and assigning classes for punctate, elongated and networked mitochondria.

To calculate the effects of ATV:TREM2 on TMRE intensity, cells were also co-incubated with NucBlue (1 drop/mL) during the TMRE incubation, and cells were washed once with Live Cell Imaging Solution and imaged using high-content microscopy (Opera Phoenix) to obtain intensity measurements per cell on 3000-4000 cells per experimental replicate.

Octet binding of antibodies to Fc gamma receptors

ATV TREM2 and ATV ISO mAbs to human Fc gamma receptors, (ACRO biosystems Fc gamma RI #FCA-H82E8, RIIA #CDA-H82E5, RIIIA #CDA-H82E9, RIIB #CDB-H82E0), binding affinities were determined by Biolayer Interferometry using an OCTET RED 384 instrument. In order to measure ATV TREM2 and ATV ASO binding affinities to each biotinylated Fc gamma receptors, biotinylated human Fc gamma receptors were immobilized onto SA-coated sensor probes (Sartorius, #185019) at a 2ug/mL concentration at a shaking speed of 1000 rpm. Fc gamma receptor-immobilized sensor probes were then plunged into increasing concentrations (0 nM to 4000 nM) of ATV TREM2 or ATV ASO diluted in 1X Kinetics buffer (Sartorius, #18-1105) for 900 seconds followed by 300 second dissociation in a blank 1X Kinetics Buffer. A 1:1 Langmuir model of simultaneous fitting of k_{on} and k_{off} was used for antigen binding kinetics analysis.

Epitope mapping by hydrogen deuterium exchange

The epitope of the recombinant human TREM2 targeted by the anti-TREM2 antibody was determined by HDX-MS at NovaBioAssays (Woburn, MA). Briefly, the recombinant human TREM2 was mixed with anti-TREM2 Fab in a deuterium oxide labeling buffer (50 mM sodium phosphate, 100 mM sodium chloride at pH 7.0) and incubated for 0 s, 60 s, 600 s, and 3600 s at 20ºC. Hydrogen/deuterium exchange was quenched by adding 4 M guanidine hydrochloride, 0.85 M TCEP buffer (final pH at 2.5). Then, the mixture was subjected to on-column pepsin/protease XIII digestion using a pepsin/protease XIII column (2.1 x 30 mm). The resultant peptides were analyzed using an UPLC-MS system comprised of a Waters Acquity UPLC coupled to a Q ExactiveTM HF Hybrid Quadrupole-Orbitrap Mass Spectrometer (Thermo). The peptides were separated on a 50 mm x 1 mm

C8 column. Peptide identification was done by searching MS/MS data against the human TREM2 sequence with Mascot. Raw MS data was processed using HDX WorkBench software for the analysis of H/D exchange MS data (J. Am. Soc. Mass Spectrom. 2012, 23 (9), 1512-1521). The deuterium levels were calculated using the average mass difference between the deuterated peptide and its native form.

Epitope mapping of ATV:TREM2 by peptide tiling

Peptide ELISAs to detect TREM2 antibody binding to tiled stalk region peptides were performed as previously described[33] except using human peptides.

Detection of TREM2 antibody cell binding by flow cytometry

hTREM2-DAP12 HEK293 cells, cTREM2-DAP12 HEK293 cells, and parental HEK293 cells expressing GFP were harvested by 0.05% trypsin and incubated at 37C for 2 hours. After incubation, cells were centrifuged and resuspended in PBS with Trypan Blue for 10 min. After staining, all cells were centrifuged and washed in FACS buffer (PBS + 0.5% BSA) twice. Mixed cells were resuspended in FACS buffer at a density of 10^6 cells/mL per cell line. The mixed cell lines were seeded at 100,000 cells per well in a 96-well v-bottom plate and incubated for 20 min at room temperature. After incubation, the cells were centrifuged and incubated with ATV:TREM2 or ATV:ISO in a dose titration from 0-300 nM for 45 min on ice. After incubation, cells were centrifuged and washed with FACS buffer three times. The cells were then incubated with secondary antibody (Alexa Fluor 647 AffiniPure F(ab')2 Fragment Goat Anti-Human IgG (H+L), Jackson ImmunoResearch Laboratories, Catalog No. 109-606-088, 1:800 dilution) for 30 min on ice without exposure to light. After incubation, the cells were washed with FACS buffer three times, resuspended in 100 µL of FACS buffer, and analyzed by flow cytometry (BD FACSCanto

II, San Jose, CA), for which 50,000 events were obtained for each sample. Mean fluorescence intensity per cells were calculated by FLowJO software and used for generation dose response binding curve.

iMG FACS analysis

50K WT iMG cells per sample were resuspended in 1X FACS buffer containing 1X PBS, 1% BSA, 1 mM EDTA. Cells were then incubated with 5 uL anti-Fc blocker (Biolegend, 422301) per 100 uL sample for 20 min on ice. Cells were then stained with BV421 conjugated CD11b antibody (Biolegend, 101235) and 100 nM biotinylated ATV:TREM2 antibody for 45 min on ice. ATV:TREM2 and isotype biotinylation was performed using EZ-link sulfo-NHS-LS-biotin kit following the manufacturer's instruction (Thermo scientific, A39257). After washing with 1X FACS buffer for 3 times, cells were then stained with APC conjugated Streptavidin (Biolegend, 405207) for 30 min on ice. After washing 3 time with 1X FACS buffer, the stained cells were then analyzed on flow cytometer Canto (BD FACSDiva software V9.0). FCS files were then imported and analyzed in FlowJo software (V10).

Cell-based shedding blocking

One day prior to assay, hTREM2-DAP12 HEK293 were plated at 50,000 cells/well on a 96 well plate coated with Poly-D-Lysine. Antibodies were diluted in HEK293 media (DMEM, 10% FBS, 1% Glutamax, 1% Penicillin-streptomycin) starting from 300 nM and proceeding in a 10-point serial dilution titration with half-log dilutions between points. The cells were dosed with the antibodies or 1uM TACE inhibitor GM6001 (Adooq Bioscience A13320-50) and incubated for 24 hours. After incubation with the antibodies, the plate

was spun down to remove debris and the supernatants collected for soluble TREM2 measurement.

Detection of mouse or human TREM2 protein levels by MSD

Soluble TREM2 was measured as follows. 96 well MSD small spot streptavidin plates (MSD - Meso Scale Discovery) were blocked with MSD Blocker-A (MSD) for 1 hour at RT. Plates were then washed 3x with TBST, then coated with biotinylated anti-TREM2 polyclonal antibody (R&D Systems BAF1828 for human, BAF1729 for mouse) at 1 ug/mL at RT for 1 hour. Standards (recombinant human or mouse TREM2 ECD prepared in house) were prepared in Assay Buffer (25% MSD Blocker-A in TBST) and serially diluted 1:4 in Assay Buffer. For cell-based samples, samples and standards were heated to 95C for 5 min in an SDS-containing buffer. Samples were diluted 1:20 for mouse CSF and 1:10 for cell supernatant. Plates were washed 3x with TBST, then 30 uL of the samples or standards were added to the plates and incubated for one hour. Subsequently, for mouse samples, primary antibody 4D9 at 110ug/mL prepared in house was directly added to the plate (3.3 uL/well), and incubated another hour at RT. Then, plates were washed 3x with TBST, and the primary detection antibody, sulfo-tagged goat anti-human TREM2 (R&D Systems AF1828 sulfo-tagged as per the MSD Gold Sulfo-tag NHS-ester kit protocol (MesoScale, R3122-A) or sulfo-tagged goat anti-human IgG (MSD, R32AJ-1) for mouse TREM2 detection, was diluted to 0.5 ug/mL in Assay Buffer, added to the plates, and incubated for one hour at room temperature. After washing with TBST, the MSD plates were developed using 2x MSD read buffer T, followed by detection using an MSD Sector plate reader (Methodical Mind, V1.0.38). MSD values were converted to absolute concentrations of TREM2.

Liposome response assay (chronic antibody exposure): Cells (30,000 iMG/well) were plated on PDL-coated 96-well plates in full serum media. After 24 hours at 37ºC, the media was exchanged for full serum media containing 100nM ATV:TREM2 antibody or isotype control. After 24 hour incubation at 37ºC, the media was removed and the cells were washed once with PBS. Cells were then dosed with either liposomes (1mg/mL in PBS) or PBS for 5 min at 37ºC, then the cells were lysed in 25uL CST (Cell Signaling Technologies) lysis buffer containing 1mM PMSF (phenylmethylsulfonylfluoride, Sigma Aldrich). Lysate was assessed for pSyk levels using AlphaLisa.

Human macrophage survival assay

Primary human donor blood was obtained from Vitalant (San Francisco, CA, USA) in 10 mL TrimaLeukoReduction chambers. Monocytes were enriched using the RosetteSep Monocyte Isolation Antibody cocktail (Stemcell technologies, 15068) following the manufacture's protocol. For antibody surface immobilization, antibodies were prepared with serial dilutions in PBS and added 50 uL per well in 96-well plates (Thermo scientific, 161093) followed by incubation at 4C overnight. On the next day, the antibody coated plates were washed once with PBS and seeded with 100K cells/well monocytes in RPMI-GlutaMAX media (Gibco, 61870-036) containing 10% FBS (Hyclone, SH30070.03), 1% Sodium Pyruvate (Gibco, 11360-070), 1% Glutamax (Gibco, 35050-061), 1% NEAA (Gibco, 11140-050), 1% Penn/Strep (Gibco, 15140-122), 5 ng/mL M-CSF (Gibco, PHC9504). Cells were maintained at 37C with 5% CO2 for 5 days. Cell viability were measured by CellTiter-Glo Luminescent cell viability assay kit (Promega, G7571) according to the manufacture's protocol. Luminescence was measured by transferring the lysate to opaque 96 well plates (Costar, 3693) and read by plate reader (Bioteck,

NEO2SMALPHA) with 20 ms Integration and 135 gain. Data obtained from a total of 4 donors were shown as biological replicates.

TfR reconstitution and cis/trans model differentiation assay

To remove endogenous TfR expression from the hTREM2-DAP12 HEK293, TfR^{RNAi} cells were generated by transducing the parental cell line with a lentiviral construct expressing dox-inducible shRNA against human TFRC gene (Horizon, V3SH7669-230884572). Stable cell line was obtained after selection with 2 ug/mL puromycin for 14 days. To initiate TFRC knockdown, cells were maintained in DMEM media (Gibco, 11965092) containing 100 ng/mL doxycycline for at least 72 h before use. For TfR reconstitution assay, full-length recombinant TfR was generated as previously described [34]. Recombinant TfR was diluted in PBS starting at 25 nM and proceeding in a 11-point titration with 2-fold dilutions between points. 10 nM TREM2 antibody was then added and the antibody mixture was incubated at 4C overnight. The solution was warmed at 37C for 1 hr on the day of use. Control assay using anti-human IgG Fc antibody (Fisher scientific, NC9915585) was performed the same way. For cis/trans differentiation assay, equal amount of the parental TREM2/DAP12 overexpressing cells were either mixed or seeded separately with the TfR^{RNAi} cells 24 h before experiment. Antibody solution containing 100 nM TREM2 antibodies were prepared in PBS. Antibody treatment was performed at 37C for 5 min, followed by alphalisa measurement of pSYK activity as previously described in Methods.

Surface and total TREM2 measurements by immunofluorescence

For evaluation of surface and total TREM2 receptor levels upon treatment with antibody, hTREM2-DAP12 HEK293 cells were dosed as above with 10nM antibody for 10 min, then

immediately put on ice and stained for surface TREM2 for 45 min with 1:250 goat anti-human TREM2 (RnD clone AF1828) in DMEM including 5% FBS and 25mM HEPES. Cells were washed 1x with PBS, then fixed for 10 min with 4% paraformaldehyde in PBS. Cells were subsequently blocked with 5% BSA in PBS for 30 min at room temperature, then stained at 1:500 Alexa 488 anti-goat (ThermoFisher A11055) for 45 min at room temperature. Cells were then imaged using an Opera Phoenix High Content Imager and images quantified using a spot finding algorithm as described above to obtain surface TREM2 levels. To measure total TREM2, cells were then blocked and permeabilized for 30 min in 0.3% Triton / 5% BSA in PBS, then stained overnight at 4C with 1:250 of goat anti-human TREM2 (RnD clone AF1828) in 0.06% Triton / 1% BSA in PBS. Cells were then washed 3x with PBS and stained at 1:500 with Alexa 488 anti-human IgG (ThermoFisher A11055) in 0.06% Triton / 1% BSA in PBS for 45 min at room temperature. Cells were stained with 1:1000 dilution of 1mg/mL DAPI (ThermoFisher 62248) in PBS for 10 min, then washed 2x with PBS and imaged using an Opera Phoenix High Content Imager and images quantified using a spot finding algorithm as described above to obtain total TREM2 levels.

Human cytokine profiling of culture media from iMG treated with ATV:TREM2

WT iMG cell were treated with 100 nM ATV:TREM2 or isotype control for 96 h. In control group, WT iMG cells was treated with 10 ng/mL LPS or PBS as vehicle control for 24 h. Culture media was then collected and frozen in -80C. Cytokine in culture media was then measured by the Human Cytokine Array/Chemokine Array 42-Plex with IL-18 (Eve Technologies, HD42).

Tissue homogenization

Fresh perfused brain was snap frozen before lysing in 10x v/w 1% NP40 (Thermo 85124) in PBS with protease and phosphatase inhibitors (Roche 04693132001 and 04906837001). Tissue was homogenized using a Tissue Lyser II (Qiagen, 85300) and 3 mm beads (Qiagen 69997) for two three-minute sessions at 27 Hz at 4ºC. Following a 20 minute incubation on ice samples were centrifuged for 20 minutes at 14,000 x g at 4ºC and the supernatant used for PK analyses and western blot.

Capillary depletion

All steps were performed at 4ºC using methods previously described[35]. Briefly, the meninges and choroid plexuses were removed from a piece of fresh perfused brain and the brain then homogenized by ten strokes with a Dounce homogenizer (smaller diameter pestle) in 3.5 mL HBSS. Cells were pelleted by centrifugation for 10 min at 1,000 x g and then resuspended in 2 mL of 17% dextran (MW 60,000; Sigma 31397) prepared in HBSS. Centrifugation for 15 min at 4,122 x g separated the parenchymal cells and myelin into the dextran layer and pelleted the vasculature. The parenchymal cells and myelin were separated into a new tube and the dextran diluted with HBSS to allow pelleting of the cells following a 15 min centrifugation at 4,122 x g. The parenchymal and vascular cell pellets were then lysed in 1% NP40 in PBS with protease and phosphatase inhibitors (Roche 04693159001 and 04906837001) and agitated using the Tissue Lyser II (Qiagen, 85300) for 30 seconds at 27 Hz. Following centrifugation at 12,700 x g for 10 min the supernatant was collected and snap frozen for use in PK, BCA, and western blot assays.

SDS PAGE and Western Blot

Tissue or capillary depletion lysates were prepared in 4X LDS sample buffer (Invitrogen NP0007) and 10X reducing agent (Invitrogen NP0009) and boiled at 95C for 10

minutes. Samples were loaded into NuPAGE 4-12% Bis-Tris Midi Protein Gels (Invitrogen WG1402 or WG1401) along with Precision Plus Protein Dual Color Ladder (Biorad 161-0374) and separated using the XCell4 SureLock system (Invitrogen, WR0100). Proteins were transferred to nitrocellulose blots (Biorad 1704159) using a BioRad Transblot Turbo system. Blots were blocked in 5% nonfat milk powder for 1 h at room temperature, incubated with primary antibodies diluted in blocking buffer (Rockland MB-070-010 TF) at 4ºC overnight, washed in three times in TBST for 15 min each, incubated in secondary antibodies in blocking buffer (Rockland) for 2 h, then washed in TBST three times for 15 min each. Blots were imaged using the Odyssey CLx and bands quantified using Image Studio Lite Software (LI-COR).

Antibody	Source	Cat	Dilution
Transferrin receptor (H68.4)	Thermo	13-6800	1:2000
CD31	CST	77699	1:1000
GAPDH	Abcam	Ab181603	1:5000
CLDN5	Invitrogen	35-2500	1:500

Immunohistochemistry

One fresh, perfused brain hemisphere was immersion fixed for 24 h in 4% PFA before gelatin embedding and sectioning (40 μm) at Neuroscience Associates. Sheets of brain sections were rinsed in PBS then blocked for three hours in 1% BSA with 0.1% fish gelatin in PBS with 0.1% Triton X-100 and 0.1% sodium azide. Sections were then incubated overnight with primary antibodies at 4ºC in in 1% BSA in PBS with 0.3% Triton X-100 (PBSX), washed three times for 15 min each in PBSX, then incubated for

three hours with secondary antibodies and DAPI in 1% BSA in PBSX at room temperature. Sections were washed three times for 15 min each in PBSX, mounted onto slides, and cover slipped using Prolong Glass Antifade Mountant.

Antibody	Source	Cat	Dilution
Iba1	Abcam	ab178847	1:500
Donkey F(ab')$_2$-anti-human IgG AlexaFluor 647	Jackson Immuno Research	709-606-149	1:250

Confocal imaging and quantification of intracellular antibody levels

To quantify the intracellular localization of huIgG, sections were imaged using a scanning confocal microscope (Leica SP8; Leica Microsystems, Inc). operated in super resolution LIGHTNING mode, acquired with a 10X/0.4 NA oil objective at a pixel size of 200 nm and processed using the Adaptive processing algorithm. Confocal z-stacks were obtained at a spacing of 4 µm, final stack size of 40 µm, were acquired for each channel using sequential scan settings from both neocortical and hippocampal brain regions and from 3-4 mice per treatment group. The intramicroglial huIgG signal was masked using an intensity-based segmentation of Iba1-positive pixels, and the resulting sum intensities were normalized to the total microglial volume within a given three-dimensional image field. The calculated mean intensities were determined for each mouse and used to calculate the mean ± SEM for each treatment.

Generation of human Trem2 BAC transgenic mouse model

Human Trem2 BAC transgenic (tg) mouse model was used to evaluate the human specific ATV:TREM2 antibody in vivo. This mouse model was generated at Denali by introduction of engineered BAC DNA CTD-2210D2 into the pronucleus of fertilized mouse

eggs from C57BL/6J mice. The engineered BAC DNA CTD-2210D2 clone covers entire human Trem2 coding region and its regulatory elements with deletion of the exon 1 from TREML1 and exon 3 from TREML2 to abolish the expression of TREML1 and TREML2. Human Trem2 BAC tg mice were backcrossed to C57BL/6J mice for three rounds and maintained as hemizygous and then further bred with $TfR^{mu/hu}$ mice to generate human TREM2 BAC tg; $TfR^{mu/hu}$ mice for in vivo studies. Mice were bred at JAX Laboratories and transferred to Denali at least two weeks before the initiation of the study for acclimation. Mice were housed in standard conditions in Denali's vivarium with ad libitum access to food and water.

SPECT/CT imaging of radiolabeled antibodies

^{125}I and ^{111}In radiolabeling

Anti-TREM2, ATV:TREM2 and isotype (ATV:ISO) were radiolabeled with ^{125}I or ^{111}In. [^{125}I]SIB labeled antibodies were produced by incubating N-Succinimidyl-3-(tri-n-butylstannyl) benzoate (ATE) with $Na^{125}I$. The crude product was purified with [^{125}I]SIB coupled to the appropriate antibody. Separately, antibodies were radiolabeled with ^{111}In once following conjugation with 1,4,7,10-tetraazacyclododecane-1,4,7,10-tetraacetic acid (DOTA). Antibodies were buffer exchanged into 0.1 M sodium bicarbonate pH 8.8 using a 0.5 mL 30 kDa Amicon centrifuge filters, then conjugated with p-SCN-Bn-DOTA prepared in 0.1M HEPES pH 8.3 at a molar ratio of 20:1 (DOTA:antibody). The reaction mixture was heated at 37°C for 90 minutes then left at room temperature overnight. The following day, the reaction solution was transferred to 0.5 mL 30 kDa Amicon tubes for purification, with the DOTA conjugated material stored at 4°C until use. For ^{111}In radiolabeling, the conjugated antibody was added to a vial containing $^{111}InCl$ which had

been neutralized with 3x volume of 0.25 M sodium acetate, pH 5.5. The reaction mixture was then incubated at 37°C for one hour. All radiolabeled antibodies were purified via Amicon filters, with radiochemical purity assessed via HPLC.

In vivo imaging

Antibodies labeled with ^{125}I or ^{111}In were separately administered to WT; hTREM2 tg; $TfR^{mu/hu}$ mice by tail vein injection (100 *μ*Ci, 200*μ*L, 1.5 mg/kg). Mice (n=48, male and female) were separated into six groups (n=4/group). Three mice per group underwent a 30-minute whole body multi-animal SPECT/CT acquisitions at 2, 24, 48, 72, and 96 h post-radiotracer administration, using a 3-bed configuration. One subject per group underwent a 30-minute whole body single-animal SPECT/CT acquisition.

Ex vivo biodistribution

Following SPECT/CT imaging at the final imaging timepoint (96 h), all subjects per group were euthanized and select tissues were resected for further radioanalysis using a gamma counter. Specifically, cerebrospinal fluid (CSF) was collected then the mice were saline-perfused and necropsied. Seven brain regions were collected for gamma counting: cerebral cortex, caudo-putamen, cerebellum, mid-brain, hindbrain, hippocampus, and hypothalamus.

SPECT image processing

Reconstructed images from the NanoScan SPECT/CTTM were generated in units of activity. Namely, the values assigned to the voxels (volume elements) comprising the 3D reconstructed SPECT images were in units of μCi. Images of multi-animal hotels were split into individual animal focused data. Reconstructed SPECT and CT images were co-

registered to one another and resampled to uniform voxel size (0.2 x 0.2 x 0.2 mm^3) using VivoQuant software (Invicro). The imaging bed was removed from the CT images.

Estimating tissue uptake from SPECT data

Regions of interest (ROIs) in the SPECT/CT data were defined using VivoQuant software. Brain regions, including whole brain and sub-regions, were generated using a 3D mouse brain atlas tool in VivoQuant. Heart ROIs were generated by placing fixed volume phantoms within the respective organs based on anatomical location on CT along with SPECT uptake where present.

Group and individual master spreadsheets were generated that include the activity concentration (percent injected dose per gram, %ID/g) at each time point for each ROI generated. SPECT/CT results from multi-animal and single-animal acquisitions were kept separate due to the differences in spatial resolution and sensitivity between the two sets of apertures used for data collection. Results were presented in units of percent injected dose per gram (%ID/g). Brain ROI results were corrected for cerebral blood flow contribution to activity concentration by using the heart ROI %ID/g as a surrogate for activity concentration in the blood pool. Blood pool activity concentration was multiplied by the fractional cerebral blood volume for whole mouse brain[36] and subtracted from the activity concentration value from the whole brain ROI.

TfR MSD assay and quantification

The mouse TfR MSD assay was developed by conjugating TfR capture and detection antibodies provided in the Mouse Transferrin Receptor Antibody ELISA kit (Abcam ab256631) to biotin or MSD Sulfo-tag NHS-Ester, respectively, according to each manufacturer's protocol (Thermo Scientific EZ-Link Sulfo-NHS-LC-Biotin and MSD

GOLD™ SULFO-TAG NHS-Ester). Briefly, using automated robotics systems (Agilent Bravo with a 96LT head and Dynamic Devices Lynx with a 96 VVP (Variable volume pipette) head) and constant plate agitation at 700RPM (Thermo Scientific™ Compact Digital Microplate Shaker plate shaker brand), an MSD Gold Streptavidin 384-well plate was blocked with 5% MSD blocker for 1 hour at room temperature (RT), washed with PBST, incubated with 0.125 μg/mL biotin-capture antibody for 1 hour at RT, washed again with PBST and incubated overnight with brain lysate at 4˚C. Following the overnight incubation, the 384-well plate was washed with PBST, incubated with 0.25μg/mL Sulfo-tag-detection antibody for 1 hour at RT, washed once more, incubated with 2x MSD read buffer for 10-15 minutes at RT and read on an MSD plate reader (Meso Sector S 600) to acquire data. MSD data was analyzed in GraphPad Prism using a Sigmoidal, 4PL standard curve to interpolate mouse TfR concentrations from MSD values. Mouse TfR concentrations were normalized to total protein concentrations (determined by standard BCA quantification) of each respective lysate. Data were subsequently analyzed using unpaired, nonparametric Student's t-test.

References

1. Kober, D.L., *et al.* Neurodegenerative disease mutations in TREM2 reveal a functional surface and distinct loss-of-function mechanisms. *Elife* **5**(2016).
2. Lee, K., Lu, R., Luther, K. & Seung, H.S. Learning and Segmenting Dense Voxel Embeddings for 3D Neuron Reconstruction. *IEEE Transactions on Medical Imaging* **40**, 3801-3811 (2021).
3. van der Walt, S., *et al.* scikit-image: image processing in Python. *PeerJ* **2**, e453 (2014).
4. Nunez-Iglesias, J., Blanch, A.J., Looker, O., Dixon, M.W. & Tilley, L. A new Python library to analyse skeleton images confirms malaria parasite remodelling of the red blood cell membrane skeleton. *PeerJ* **6**, e4312 (2018).
5. Karperien, A., Ahammer, H. & Jelinek, H. Quantitating the subtleties of microglial morphology with fractal analysis. *Frontiers in Cellular Neuroscience* **7**(2013).

6. Pedregosa, F., *et al.* Scikit-learn: Machine Learning in Python. *J. Mach. Learn. Res.* **12**, 2825–2830 (2011).

8. Hao, Y., *et al.* Integrated analysis of multimodal single-cell data. *Cell* **184**, 3573-3587 e3529 (2021).

9. He, D., *et al.* Alevin-fry unlocks rapid, accurate and memory-frugal quantification of single-cell RNA-seq data. *Nature Methods* **19**, 316-322 (2022).

10. Zhu, A., Srivastava, A., Ibrahim, J.G., Patro, R. & Love, M.I. Nonparametric expression analysis using inferential replicate counts. *Nucleic Acids Research* **47**, e105-e105 (2019).

11. Amezquita, R.A., *et al.* Orchestrating single-cell analysis with Bioconductor. *Nat Methods* **17**, 137-145 (2020).

12. Zeisel, A., *et al.* Molecular Architecture of the Mouse Nervous System. *Cell* **174**, 999-1014 e1022 (2018).

13. Aran, D., *et al.* Reference-based analysis of lung single-cell sequencing reveals a transitional profibrotic macrophage. *Nat Immunol* **20**, 163-172 (2019).

14. van den Brink, S.C., *et al.* Single-cell sequencing reveals dissociation-induced gene expression in tissue subpopulations. *Nat Methods* **14**, 935-936 (2017).

15. McCarthy, D.J., Campbell, K.R., Lun, A.T. & Wills, Q.F. Scater: pre-processing, quality control, normalization and visualization of single-cell RNA-seq data in R. *Bioinformatics* **33**, 1179-1186 (2017).

16. Ritchie, M.E., *et al.* limma powers differential expression analyses for RNA-sequencing and microarray studies. *Nucleic Acids Research* **43**, e47-e47 (2015).

17. Korotkevich, G., *et al.* Fast gene set enrichment analysis. *bioRxiv*, 060012 (2021).

18. Liberzon, A., *et al.* The Molecular Signatures Database (MSigDB) hallmark gene set collection. *Cell Syst* **1**, 417-425 (2015).

19. Dobin, A., *et al.* STAR: ultrafast universal RNA-seq aligner. *Bioinformatics* **29**, 15-21 (2013).

20. Liao, Y., Smyth, G.K. & Shi, W. featureCounts: an efficient general purpose program for assigning sequence reads to genomic features. *Bioinformatics* **30**, 923-930 (2014).

21. Nugent, A.A., *et al.* TREM2 Regulates Microglial Cholesterol Metabolism upon Chronic Phagocytic Challenge. *Neuron* **105**, 837-854 e839 (2020).

22. Law, C.W., Chen, Y., Shi, W. & Smyth, G.K. voom: Precision weights unlock linear model analysis tools for RNA-seq read counts. *Genome Biol* **15**, R29 (2014).

23. Huber, W., *et al.* Orchestrating high-throughput genomic analysis with Bioconductor. *Nat Methods* **12**, 115-121 (2015).

24. Overhoff, F., *et al.* Automated Spatial Brain Normalization and Hindbrain White Matter Reference Tissue Give Improved [(18)F]-Florbetaben PET Quantitation in Alzheimer's Model Mice. *Front Neurosci* **10**, 45 (2016).

25. Reifschneider, A., *et al.* Loss of TREM2 rescues hyperactivation of microglia, but not lysosomal deficits and neurotoxicity in models of progranulin deficiency. *EMBO J* **41**, e109108 (2022).

26. Deussing, M., *et al.* Coupling between physiological TSPO expression in brain and myocardium allows stabilization of late-phase cerebral [(18)F]GE180 PET quantification. *Neuroimage* **165**, 83-91 (2018).

27. Biechele, G., *et al.* Pre-therapeutic microglia activation and sex determine therapy effects of chronic immunomodulation. *Theranostics* **11**, 8964-8976 (2021).
28. Schiffer, W.K., Mirrione, M.M. & Dewey, S.L. Optimizing experimental protocols for quantitative behavioral imaging with 18F-FDG in rodents. *J Nucl Med* **48**, 277-287 (2007).
29. Xiang, X., *et al.* Microglial activation states drive glucose uptake and FDG-PET alterations in neurodegenerative diseases. *Sci Transl Med* **13**, eabe5640 (2021).
30. Logan, J., *et al.* Graphical analysis of reversible radioligand binding from time-activity measurements applied to [N-11C-methyl]-(-)-cocaine PET studies in human subjects. *J Cereb Blood Flow Metab* **10**, 740-747 (1990).
31. Sacher, C., *et al.* Longitudinal PET Monitoring of Amyloidosis and Microglial Activation in a Second-Generation Amyloid-beta Mouse Model. *J Nucl Med* **60**, 1787-1793 (2019).
32. Ma, Y., *et al.* A three-dimensional digital atlas database of the adult C57BL/6J mouse brain by magnetic resonance microscopy. *Neuroscience* **135**, 1203-1215 (2005).
33. Schlepckow, K., *et al.* Enhancing protective microglial activities with a dual function TREM2 antibody to the stalk region. *EMBO Mol Med* **12**, e11227 (2020).
34. Arguello, A., *et al.* Molecular architecture determines brain delivery of a transferrin receptor-targeted lysosomal enzyme. *J Exp Med* **219**(2022).
35. Kariolis, M.S., *et al.* Brain delivery of therapeutic proteins using an Fc fragment blood-brain barrier transport vehicle in mice and monkeys. *Sci Transl Med* **12**(2020).
36. Chugh, B.P., *et al.* Measurement of cerebral blood volume in mouse brain regions using micro-computed tomography. *Neuroimage* **47**, 1312-1318 (2009).
