# Supplementary material for: A TREM2-activating antibody with a blood–brain barrier transport vehicle enhances microglial metabolism in Alzheimer’s disease models
Source: Nat Neurosci. 2023 Jan 12;26(3):416–29. doi: 10.1038/s41593-022-01240-0 (PMC9991924; doi:10.1038/s41593-022-01240-0)
Supplement: Supplementary file 2 — Reporting Summary [file 41593_2022_1240_MOESM2_ESM.pdf]

nature portfolio

Corresponding author(s): Kathryn M. Monroe, Pascal E. Sanchez

Last updated by author(s): Sep 8, 2022

Reporting Summary

Nature Portfolio wishes to improve the reproducibility of the work that we publish. This form provides structure for consistency and transparency in reporting. For further information on Nature Portfolio policies, see our Editorial Policies and the Editorial Policy Checklist.

Statistics

For all statistical analyses, confirm that the following items are present in the figure legend, table legend, main text, or Methods section.

n/a	Confirmed	
☐	☒	The exact sample size (*n*) for each experimental group/condition, given as a discrete number and unit of measurement
☐	☒	A statement on whether measurements were taken from distinct samples or whether the same sample was measured repeatedly
☐	☒	The statistical test(s) used AND whether they are one- or two-sided<br>*Only common tests should be described solely by name; describe more complex techniques in the Methods section.*
☒	☐	A description of all covariates tested
☐	☒	A description of any assumptions or corrections, such as tests of normality and adjustment for multiple comparisons
☐	☒	A full description of the statistical parameters including central tendency (e.g. means) or other basic estimates (e.g. regression coefficient) AND variation (e.g. standard deviation) or associated estimates of uncertainty (e.g. confidence intervals)
☐	☒	For null hypothesis testing, the test statistic (e.g. F, t, r) with confidence intervals, effect sizes, degrees of freedom and P value noted<br>*Give P values as exact values whenever suitable.*
☒	☐	For Bayesian analysis, information on the choice of priors and Markov chain Monte Carlo settings
☒	☐	For hierarchical and complex designs, identification of the appropriate level for tests and full reporting of outcomes
☒	☐	Estimates of effect sizes (e.g. Cohen's *d*, Pearson's *r*), indicating how they were calculated

Our web collection on statistics for biologists contains articles on many of the points above.

Software and code

Policy information about availability of computer code

Data collection	ImageStudioLite software (Li-COR, Version 5.2.5), EnVision Workstation software (Perkin Elmer, Version #1.14.3049.528), Opera Phenix High Content Imager Harmony software (Perkin Elmer, Version v5.1.2167.302), MSD software (Methodical Mind, V1.0.38),Zeiss Axioscan.Z1 slide scanner (ZEN blue v3.5.093.00005, Carl Zeiss Microscopy), spinning disc confocal microscope (Zeiss AxioObserver Z1, Carl Zeiss Microscopy, ZEN blue v2.6.18299.3), canning confocal microscope (Leica SP8; Leica Microsystems, Inc, Leica Application Suite X v3.5.7.23225), flow cytometer Canto/Aria III (BD FACSDiva software V9.0), Tera-Tomo 3D (Mediso Ltd, Hungary).
Data analysis	Prism Version 9.3.1 (350), R Studio (Version 1.4.1717), Python (Version 3.10.8), STAR v2.7.1a, scRNAseq Seraut (Version 4),Bulk RNAseq RNAseq: Bioconductor (version 3.13),Cell Ranger (v7.0.0 for WT study; v6.1.1 for AD study), Imaris (Bitplane, V9.9.1), PMOD (V3.5, PMOD technologies, Switzerland). All of the code used to process and analyze these data are made available via GitHub: https://github.com/denalitherapeutics/Lengerich_natneuro_2022. R packages used are listed via Github here: https://github.com/denalitherapeutics/Lengerich_natneuro_2022/blob/main/renv.lock.

For manuscripts utilizing custom algorithms or software that are central to the research but not yet described in published literature, software must be made available to editors and reviewers. We strongly encourage code deposition in a community repository (e.g. GitHub). See the Nature Portfolio guidelines for submitting code & software for further information.

Data

Policy information about availability of data

All manuscripts must include a data availability statement. This statement should provide the following information, where applicable:

- Accession codes, unique identifiers, or web links for publicly available datasets
- A description of any restrictions on data availability
- For clinical datasets or third party data, please ensure that the statement adheres to our policy

Raw and processed single-cell/bulk RNA-seq data have been deposited at the NCBI GEO repository under accession numbers: GSE198987, GSE199154, GSE200275, and GSE209912. The metabolomics data have been uploaded to the MetaboLights repository with study ID MTBLS6543.

Field-specific reporting

Please select the one below that is the best fit for your research. If you are not sure, read the appropriate sections before making your selection.

☒ Life sciences ☐ Behavioural & social sciences ☐ Ecological, evolutionary & environmental sciences

For a reference copy of the document with all sections, see nature.com/documents/nr-reporting-summary-flat.pdf

Life sciences study design

All studies must disclose on these points even when the disclosure is negative.

Sample size	No statistical methods were used to predetermine sample size. Sample sizes were chosen as standard in the field (Schlepckow et al., EMBO 2020). For in vivo study, sample size "n" represents the number of animals used. For in vitro study, sample size "n" represents the number of independent experiments performed.
Data exclusions	No data were excluded from the study.
Replication	The exact number of repetitions (individual data points from each cells and/or animal) are indicated in figures or figure legends. For in vitro experiments, at least 3 independent experiments were performed as biological replication. All replication were successful.
Randomization	For in vivo experiments, animals/samples were randomized before drug treatment to cover sex/genotype/drugs/etc for balancing potential batch effects. No randomization was performed for in vitro studies.
Blinding	The samples were not blinded during initial study planning to ensure that the number of groups of mice were randomized and balanced, while age and sex matched. RNAseq datasets were not blinded for analysis. Image analysis and ex-vivo assays were performed in a blinded fashion. In vitro studies were performed unblinded. The PET imaging analysis was not blinded, however we used an automated pipeline72,73 such that the operator cannot influence/bias the results since the coregistration and the voi extraction is a defined procedure.

Reporting for specific materials, systems and methods

We require information from authors about some types of materials, experimental systems and methods used in many studies. Here, indicate whether each material, system or method listed is relevant to your study. If you are not sure if a list item applies to your research, read the appropriate section before selecting a response.

Materials & experimental systems

n/a	Involved in the study	
☐	☒	Antibodies
☐	☒	Eukaryotic cell lines
☒	☐	Palaeontology and archaeology
☐	☒	Animals and other organisms
☒	☐	Human research participants
☒	☐	Clinical data
☒	☐	Dual use research of concern

Methods

n/a	Involved in the study	
☒	☐	ChIP-seq
☐	☒	Flow cytometry
☒	☐	MRI-based neuroimaging

Antibodies

Antibodies used	ATV:TREM2, anti-TREM2, monovalent versions thereof, ATV:4D9, anti-4D9, and isotype controls were produced at Denali according to Materials and Methods. Commercial Antibody used are listed below: Name Application Dilution Source Cat# Clone<br>mTOR (7C10) WB 1/500 CST 2983T 7C10<br>Phospho-mTOR (Ser2448) WB 1/500 CST 5536T D9C2<br>Phospho-Akt (Ser473) WB 1/250 CST 9271T Polyclonal

AKT WB 1/500 CST 4691T C67E7
Phospho-RPS6 (Ser235/236) WB 1/500 CST 4858T D57.2.2E
Phospho-GSK-3β (Ser9) WB 1/500 CST 5558T D85E12
β-Actin WB 1/1000 CST 58169S E4D9Z
Phospho-4E-BP1 (Thr37/46) WB 1/500 CST 2855T 236B4
Phospho-Erk1/2 (Thr202/Tyr204) WB 1/1000 CST 4370S D13.14.4E
TREM2 WB, IP, ICC 1/500 R and D systems AF1828 Polyclonal
CD31 WB 1/500 CST 77699 D8V9E
GAPDH WB 1/500 Abcam Ab181603 EPR16884
CLDN5 WB 1/500 Invitrogen 35-2500 4C3C2
normal goat IgG (R&D, AB-108-C) IP IgG control 2 ug per 400-500 ug lysate R and D systems AB-108-C Polyclonal
TfR_DNLI_#1 IP NA Denali Therapeutics NA DC0002
TfR_DNLI_#2 TfR blocking NA Denali Therapeutics NA DC0637
TfR ICC, WB 1/250 Thermofisher 13-6800 H68.4
EEA1 ICC 1/250 BD Biosciences 610457 14
phospho-Syk (Tyr525/526) ICC 1/250 CST 2710 C87C1
CD11b-BV421 FACS 1/100 BioLegend 101251, 101235 M1/70
CD45-APC FACS 1/100 BD Biosciences 559864 30-F11
anti-mouse CD16/32 FACS 1/20 BioLegend 101320 93
Axl IHC 1/25 R and D systems AF854 Polyclonal
Iba1_guinea pig IHC 1/500 Synaptic Systems HS-234 308 Gp311H9
Iba1_goat IHC 1/500 Novus NB100-1028 Polyclonal
Iba1_rabbit IHC 1/500 abcam ab178847 monoclonal
CD74 IHC 1/500 abcam ab245692 In-1
donkey anti-human IgG ELISA 1 ug/mL Jackson Immunoresearch 709-006-098 Polyclonal
HRP conjugated goat anti-human IgG ELISA 0.02 ug/mL Jackson Immunoresearch 109-036-098 Polyclonal
biotinylated anti-human TREM2 polyclonal antibody MSD 1 ug/mL R and D systems BAF1828 Polyclonal
biotinylated anti-mouse TREM2 polyclonal antibody (R&D Systems BAF1828 for human, BAF1729 for mouse MSD 1 ug/mL R and D systems BAF1729 Polyclonal
anti-mouse transferrin receptor antibody kit MSD capture.ab=0.125 ug/mL; detector.ab= 0.25 ug/mL Abcam ab256631 monoclonal

Validation

All Denali antibodies were characterized as described in the manuscript for specificity and binding by biacore and FACS-based cell binding using KO cell lines and overexpression cell lines (See Extended data figure 3, Table 1).
Commercial antibodies were validated by suppliers as shown below: Antibody Validation_link
mTOR (7C10) https://www.cellsignal.com/products/primary-antibodies/mtor-7c10-rabbit-mab/2983
Phospho-mTOR (Ser2448) https://www.cellsignal.com/products/primary-antibodies/phospho-mtor-ser2448-d9c2-xp-rabbit-mab/5536?site-search-type=Products&N=4294956287&Ntt=5536t&fromPage=plp&_requestid=3313777
Phospho-Akt (Ser473) https://www.cellsignal.com/products/primary-antibodies/phospho-akt-ser473-antibody/9271?site-search-type=Products&N=4294956287&Ntt=9271t&fromPage=plp&_requestid=3313824
AKT https://www.cellsignal.com/products/primary-antibodies/akt-pan-c67e7-rabbit-mab/4691?site-search-type=Products&N=4294956287&Ntt=4691t&fromPage=plp&_requestid=3325825
Phospho-RPS6 (Ser235/236) https://www.cellsignal.com/products/primary-antibodies/phospho-s6-ribosomal-protein-ser235-236-d57-2-2e-xp-rabbit-mab/4858?site-search-type=Products&N=4294956287&Ntt=4858t&fromPage=plp&_requestid=3314019
Phospho-GSK-3β (Ser9) https://www.cellsignal.com/products/primary-antibodies/phospho-gsk-3b-ser9-d85e12-xp-rabbit-mab/5558?site-search-type=Products&N=4294956287&Ntt=5558t&fromPage=plp&_requestid=3314040
β-Actin https://www.cellsignal.com/products/primary-antibodies/b-actin-e4d9z-mouse-mab/58169?site-search-type=Products&N=4294956287&Ntt=58169s&fromPage=plp&_requestid=3314118
Phospho-4E-BP1 (Thr37/46) https://www.cellsignal.com/products/primary-antibodies/phospho-4e-bp1-thr37-46-236b4-rabbit-mab/2855?site-search-type=Products&N=4294956287&Ntt=2855t&fromPage=plp&_requestid=3314156
Phospho-Erk1/2 (Thr202/Tyr204) https://www.cellsignal.com/products/primary-antibodies/phospho-p44-42-mapk-erk1-2-thr202-tyr204-d13-14-4e-xp-rabbit-mab/4370?site-search-type=Products&N=4294956287&Ntt=4370s&fromPage=plp&_requestid=3318000
TREM2 https://www.rndsystems.com/products/human-trem2-antibody_af1828
CD31 https://www.cellsignal.com/products/primary-antibodies/cd31-pecam-1-d8v9e-xp-rabbit-mab/77699
GAPDH https://www.abcam.com/gapdh-antibody-epr16884-loading-control-ab181603.html
CLDN5 https://www.thermofisher.com/antibody/product/Claudin-5-Antibody-clone-4C3C2-Monoclonal/35-2500
normal goat IgG (R&D, AB-108-C) https://www.rndsystems.com/products/normal-goat-igg-control_ab-108-c
TfR_DNLI_#1 https://www.science.org/doi/10.1126/scitranslmed.aay1359?url_ver=Z39.88-2003&rfr_id=ori:rid:crossref.org&rfr_dat=cr_pub%20%200pubmed
TfR_DNLI_#2 https://www.science.org/doi/10.1126/scitranslmed.aay1359?url_ver=Z39.88-2003&rfr_id=ori:rid:crossref.org&rfr_dat=cr_pub%20%200pubmed
TfR https://www.thermofisher.com/antibody/product/Transferrin-Receptor-Antibody-clone-H68-4-Monoclonal/13-6800
EEA1 https://www.bdbiosciences.com/en-us/products/reagents/microscopy-imaging-reagents/immunofluorescence-reagents/purified-mouse-anti-eea1.610457
phospho-Syk (Tyr525/526) https://www.cellsignal.com/products/primary-antibodies/phospho-syk-tyr525-526-c87c1-rabbit-mab/2710
CD11b-BV421 https://www.citeab.com/antibodies/2082933-101251-brilliant-violet-421-anti-mouse-human-cd11b
CD45-APC https://www.bdbiosciences.com/en-us/products/reagents/flow-cytometry-reagents/research-reagents/single-color-antibodies-ruo/apc-rat-anti-mouse-cd45.559864
anti-mouse CD16/32 https://www.biolegend.com/en-us/punchout/search-results/trustain-fcx-anti-mouse-cd16-32-antibody-5683?GroupID=BLG9237
Axl https://www.rndsystems.com/products/mouse-axl-antibody_af854?gclid=Cj0KCQiA4OybBhCzARIsAIcfn9nmYA1ndIVozmw91Qy2ga_a4D_VN3XomInOEd8lq8SERFV0Y7Ou2PsaAl4eEALw_wcB&gclsrc=aw.ds
Iba1_guinea pig https://www.sysy.com/product/HS-234308
Iba1_goat https://www.novusbio.com/products/aif-1-iba1-antibody_nb100-1028
Iba1_rabbit https://www.abcam.com/iba1-antibody-epr16589-ab178847.html

CD74 https://www.abcam.com/cd74-antibody-in-1-ab245692.html
donkey anti-human IgG https://www.jacksonimmuno.com/catalog/products/709-006-098
HRP conjugated goat anti-human IgG https://www.jacksonimmuno.com/catalog/products/109-036-098
biotinylated anti-human TREM2 polyclonal antibody https://www.rndsystems.com/products/human-trem2-biotinylated-antibody_baf1828
biotinylated anti-mouse TREM2 polyclonal antibody (R&D Systems BAF1828 for human, BAF1729 for mouse https://www.rndsystems.com/products/mouse-trem2-biotinylated-antibody_baf1729
anti-mouse transferrin receptor antibody kit https://www.abcam.com/mouse-transferrin-receptor-antibody-pair-bsa-and-azide-free-ab256631.html

Eukaryotic cell lines

Policy information about cell lines

Cell line source(s)	HEK293 (RRID:CVCL_0045); iPSC1 (RRID:CVCL_D086); Expi293F (RRID:CVCL_D615)
Authentication	Cell lines were not authenticated, but TREM2 expression was regularly validated.
Mycoplasma contamination	Confirmed negative for mycoplasma in house.
Commonly misidentified lines (See ICLAC register)	These cell lines were not listed in the commonly misidentified lines from ICLAC registry.

Animals and other organisms

Policy information about studies involving animals; ARRIVE guidelines recommended for reporting animal research

Laboratory animals	The genotype, age and sex information of mouse models used in this study are summarized in Supplementary Table 2. Mice are maintained on C57BL/6J genetic background. Mouse husbandry and experimental procedures were approved by Denali Institutional Animal Care and Use Committee. All animal experiments at DZNE were performed in accordance with animal-handling laws of the state of Bavaria (Germany). Housing conditions included standard pellet food and water provided ad libitum, a 12-h light–dark cycle at temperature of 22°C with maximal 5 mice per cage and cage replacement once per week with regular health monitoring. Animals used in this study: WT; TfRmu/hu mice, mixed sex, from 2 to 8 months of age; WT;AppSAA; TfRmu/hu mice, male, 8 months; hTREM2 tg; TfRmu/hu mice, mixed sex, from 3 to 6 months of age; 5xFAD; hTREM2 tg; TfRmu/hu, mixed sex, 4.5 months. Female Lewis rats at 8 weeks of age were purchased from Charles River Laboratories (Strain Code 004) for antibody discovery for the TREM2 campaign.
Wild animals	No wild animals were used in the study.
Field-collected samples	No field collected samples were used in the study.
Ethics oversight	Denali Institutional Animal Care and Use Committee

Note that full information on the approval of the study protocol must also be provided in the manuscript.

Flow Cytometry

Plots

Confirm that:

☒ The axis labels state the marker and fluorochrome used (e.g. CD4-FITC).

☒ The axis scales are clearly visible. Include numbers along axes only for bottom left plot of group (a 'group' is an analysis of identical markers).

☒ All plots are contour plots with outliers or pseudocolor plots.

☒ A numerical value for number of cells or percentage (with statistics) is provided.

Methodology

Sample preparation	Ex vivo phagocytosis: Mice were taken down two days post antibody treatment and brain was dissected out after PBS perfusion for single cell dissociation by the Adult Brain Dissociation Kit (Miltenyi Biotec, 130-107-677), according to the manufacturer's protocol. Microglia number was measured by FACS using CountBright Absolute Counting Beads (Invitrogen, C36950) and samples were diluted to 500 microglia/ul in DPBS+0.5%BSA. 100ul of the resulting cell suspension was then mixed with 100ul of pHrodo-green labeled myelin (50ug/ml in DPBS+0.5%BSA) or FAM-Abeta (200 nM in DPBS + 0.5% BSA) and incubated at 37°C for 45min with occasional gentle mixing. Cell suspensions were then washed and stained with the following antibodies in FACS buffer (1% fatty acid-free BSA and 1mM EDTA in PBS) for 25 minutes on ice: CD11b-BV421 (BioLegend, 101251) and Mouse Fc blocker (anti-mouse CD16/32, BioLegend, 101320). Cells were washed with FACS buffer, resuspended in FACS buffer with Propidium Iodide (Miltenyi, 130-93-233) and strained through a 100 µm filter before flow cytometry analysis on a BD FACS ARIA III. The percentage of myelin positive microglia (pHrodo-green+, CD11b+) and Abeta positive microglia (FAM+, CD11b+) in the total CD11b+ microglial population was calculated.

Instrument	BD FACSCanto II and BD FACS ARIA III, San Jose, CA
Software	FlowJo
Cell population abundance	Cell populations evaluated are not rare
Gating strategy	Single cells were separated from debris by FSC and SSC characteristics. Live microglia were identified as a population of CD11b+ and Propidium Iodide-low cells. pHrodo-myelin or FAM-Abeta uptake was then quantified in 20,000 microglia recorded from each sample.

☒ Tick this box to confirm that a figure exemplifying the gating strategy is provided in the Supplementary Information.
