# Supplementary figures and images for: A TREM2-activating antibody with a blood–brain barrier transport vehicle enhances microglial metabolism in Alzheimer’s disease models
Source: Nat Neurosci. 2023 Jan 12;26(3):416–29. doi: 10.1038/s41593-022-01240-0 (PMC9991924; doi:10.1038/s41593-022-01240-0)

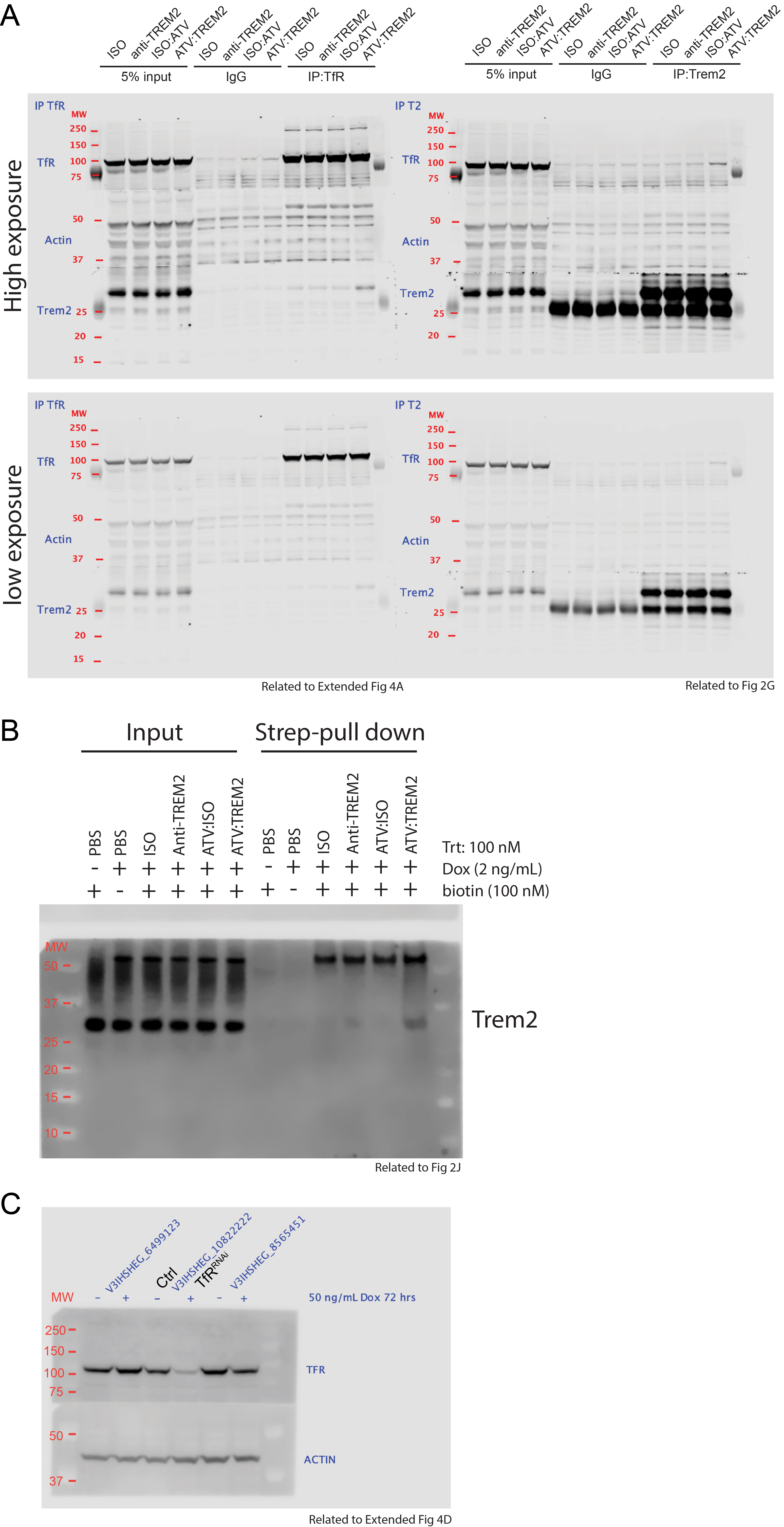

Supplement: Source Data Fig. 1 — Unprocessed western blots [file 41593_2022_1240_MOESM4_ESM.jpg]

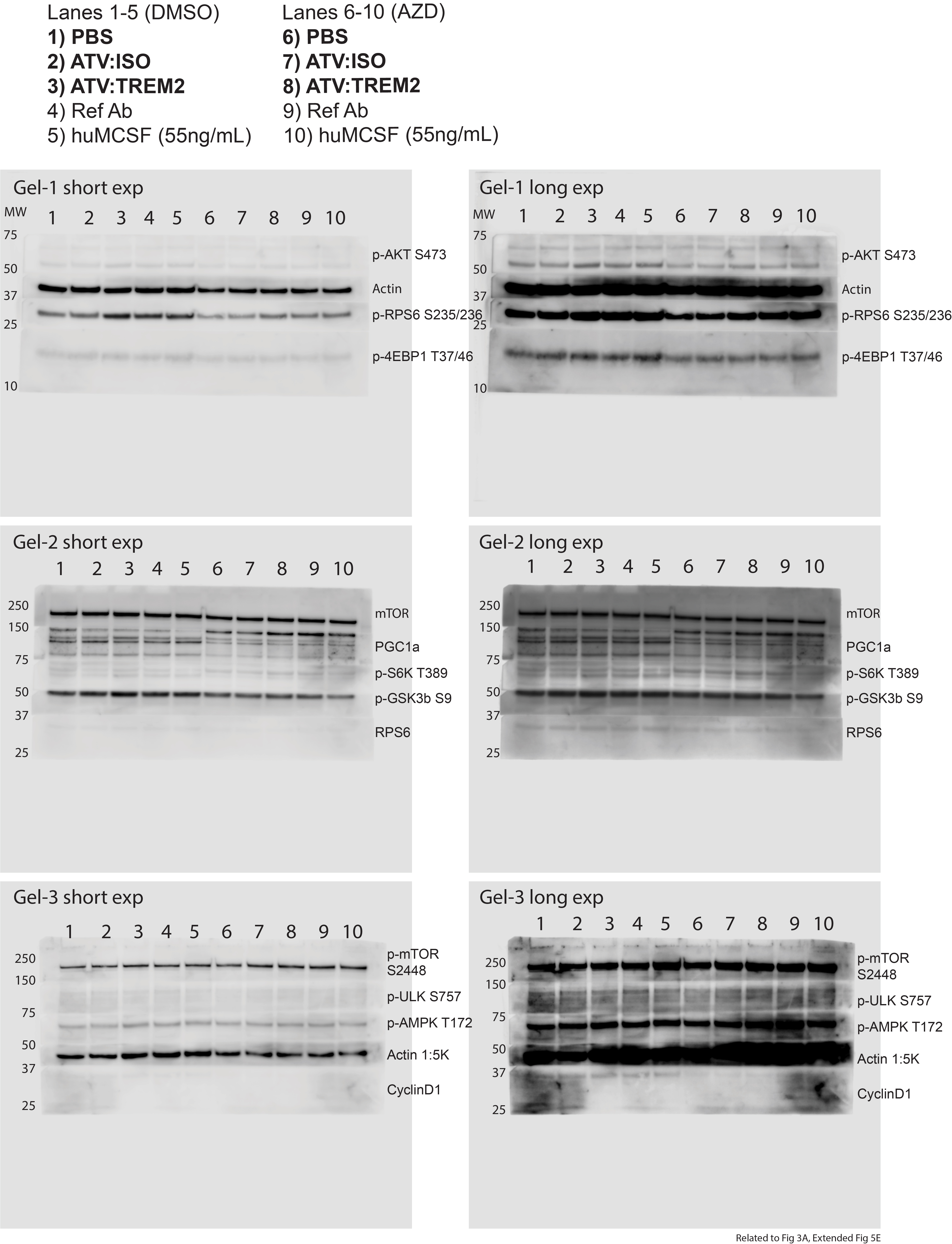

Supplement: Source Data Fig. 2 — Unprocessed western blots [file 41593_2022_1240_MOESM5_ESM.jpg]

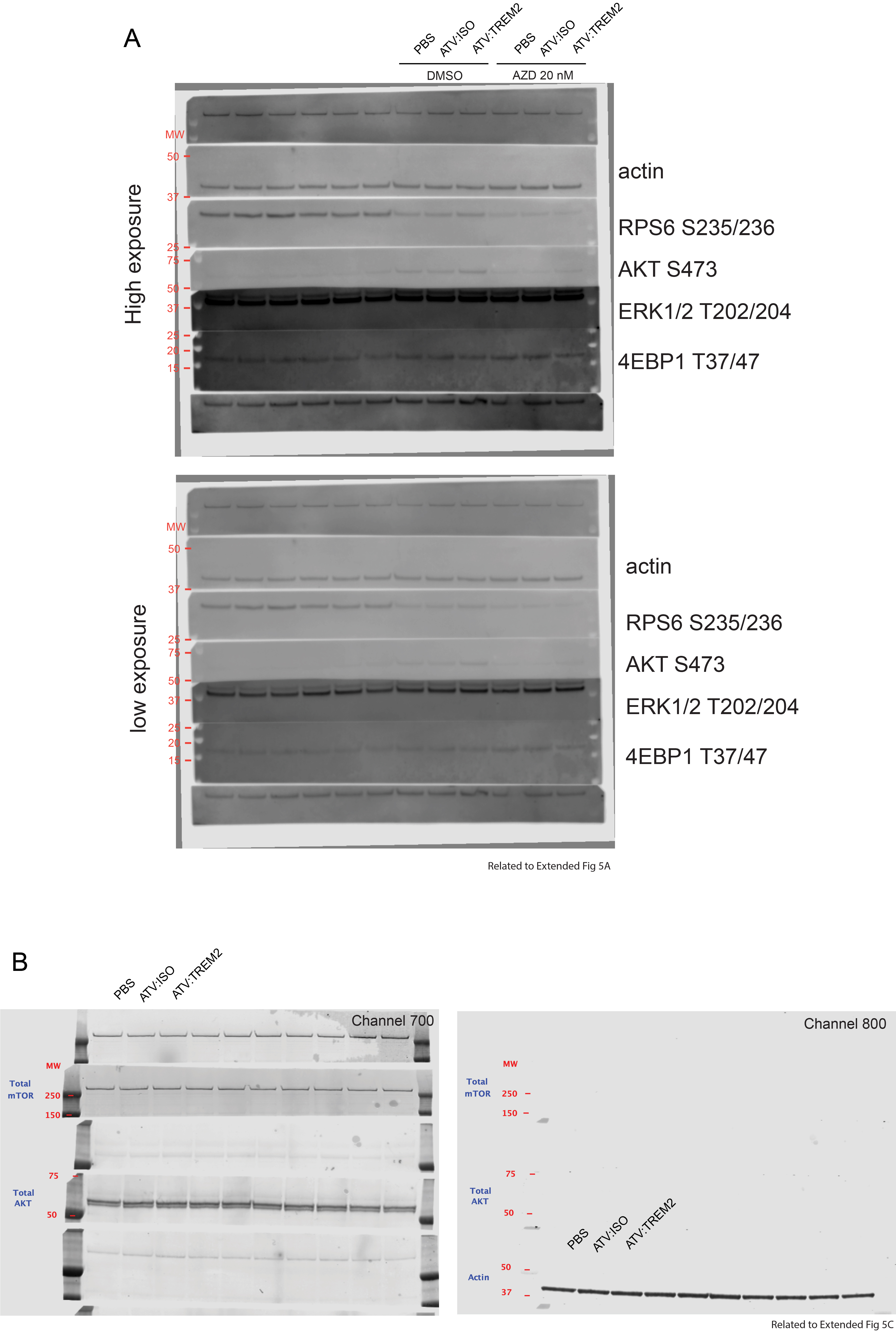

Supplement: Source Data Fig. 3 — Unprocessed western blots [file 41593_2022_1240_MOESM6_ESM.jpg]
